# Recent Advances in Synthetic Strategies and Medicinal Properties of Thienopyridines

**DOI:** 10.3390/ijms27208962

**Published:** 2026-10-09

**Authors:** Anfisa Ermolaeva, Svetlana Baykova, Sergey Baykov, Roman Ivanov, Ekaterina Minskaia

**Affiliations:** 1 Translational Medicine Research Center, Sirius University of Science and Technology, Olympic Ave., 1, Sirius 354340, Russia; ermolaeva.am@talantiuspeh.ru (A.E.); ivanov.ra@talantiuspeh.ru (R.I.); 2 Department of Physical Organic Chemistry, Institute of Chemistry, Saint Petersburg State University, 7/9 Universitetskaya Nab., Saint Petersburg 199034, Russia

**Keywords:** heterocycles, cyclization, condensation, drug discovery, medicinal chemistry, agrochemistry

## Abstract

This review provides an updated overview of recent advances (2024–present) in the synthesis and medicinal applications of thienopyridines and their fused derivatives. Synthetic methodologies are categorized into three strategic approaches: (i) thiophene ring construction onto a preformed pyridine core; (ii) pyridine ring annulation onto a thiophene scaffold—ranging from classical Friedländer and Gould–Jacobs reactions to modern metal-catalyzed, photo-induced, and electrochemical transformations; and (iii) multicomponent reactions for the simultaneous assembly of both heterocyclic rings. Furthermore, the strategic functionalization of preformed thienopyridine scaffolds to access complex tri- and polyheterocyclic systems is discussed. From a biological perspective, the review highlights the scaffold’s expanding therapeutic repertoire, including antibacterial, antifungal, and antiviral activities; potent anticancer applications targeting tumor-associated enzymes and emerging Proteolysis Targeting Chimera (PROTAC)/Deubiquitinase-Targeting Chimera (DUBTAC) technologies; and modulation of neurological, neuropsychiatric, and metabolic disorders. Finally, recent progress in the agrochemical utility of thienopyridines as insecticides and herbicide safeners is examined. Collectively, this work underscores the enduring relevance of the thienopyridine scaffold as a versatile platform for next-generation drug and agrochemical discovery.

## 1. Introduction

**Figure 1:**
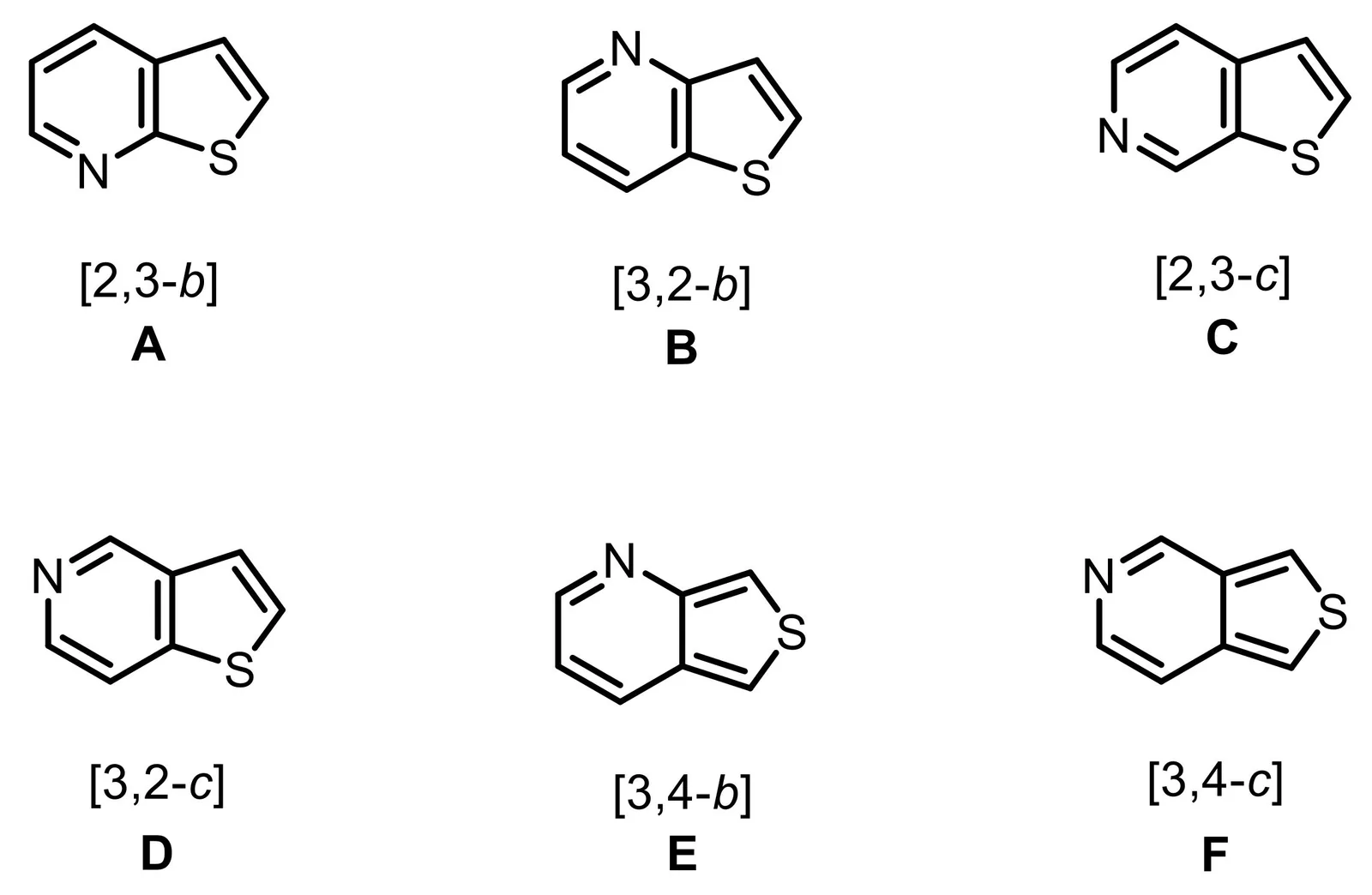
Possible isomers of thienopyridine scaffold: (**A**) [2,3-*b*]; (**B**) [3,2-*b*]; (**C**) [3,2-*c*]; (**D**) [3,2-*c*]; (**E**) [3,4-*b*]; (**F**) [3,4-*c*].

Thienopyridines are bicyclic compounds representing a system of a π-electron-rich thiophene ring fused via a C–C bond to a π-electron-deficient pyridine core. Thienopyridines exist in six different isomeric forms, A–F (Figure 1) [1]. The first thienopyridine compound was obtained in 1913 [2]. To date, synthetic methods and applications of thieno [2,3-*b*]pyridines (Figure 1, form A) and thieno [3,2-*b*]pyridines (Figure 1, form B) have been extensively studied. Isomers [2,3-*c*] (Figure 1, form C), [3,2-*c*] (Figure 1, form D), [3,4-*b*] (Figure 1, form E), and [3,4-*c*] (Figure 1, form F) have been obtained synthetically; however, studies of thieno [3,2-*c*]pyridines and thieno [2,3-*c*]pyridines are significantly limited due to more complicated synthesis. In particular, they were first obtained much later than the other isomers [3].

**Figure 2:**
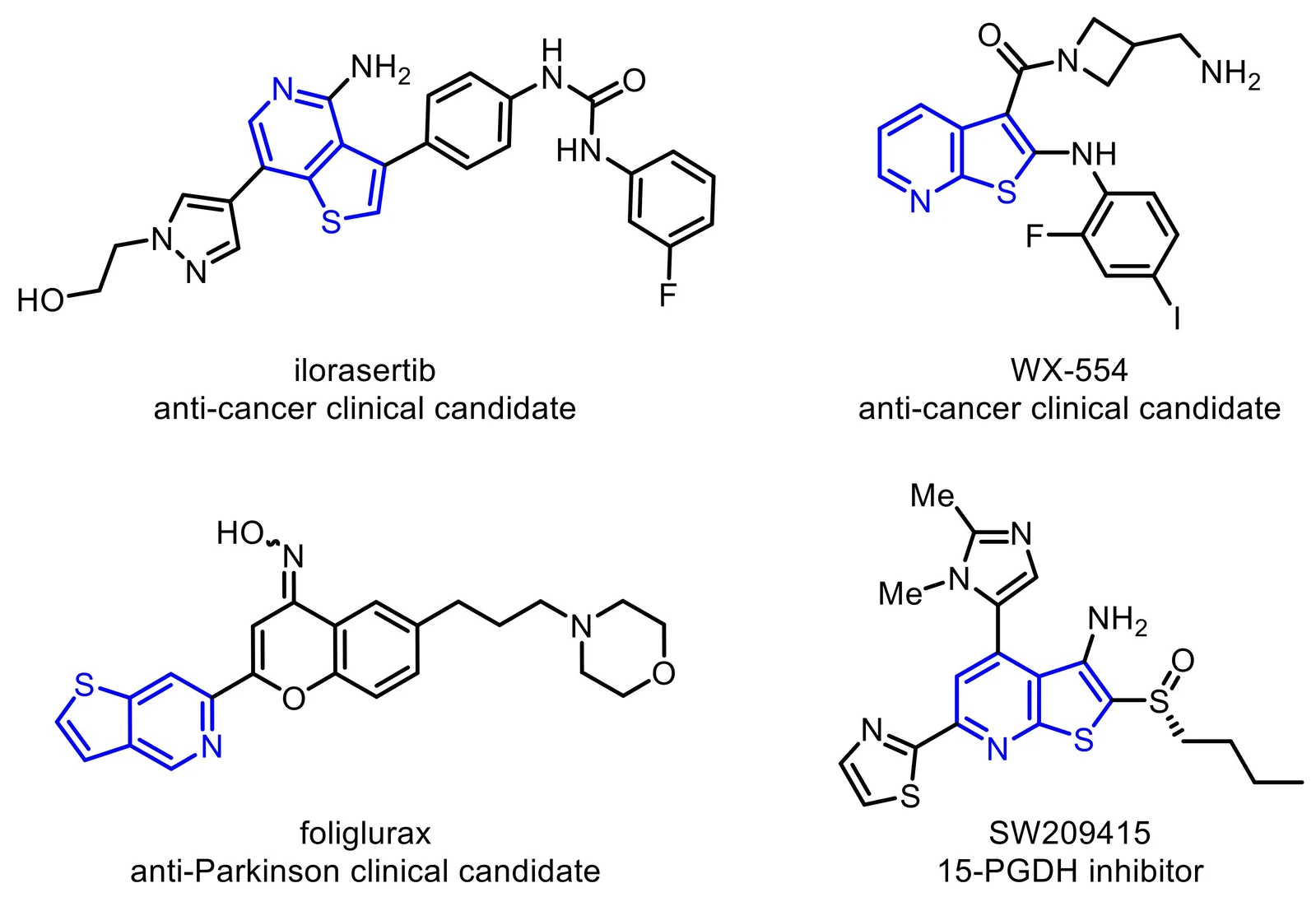
Examples of thienopyridines demonstrating therapeutic potential.

The unique π-electronic structure of the thienopyridine framework makes it an attractive scaffold for the development of active pharmaceutical ingredients and agrochemicals [4]. This attractiveness arises from its extended conjugation and planar geometry, which facilitate strong π∙∙∙π stacking interactions with aromatic amino acid residues in protein-binding sites [5,6]. In addition, the thienopyridine ring system shows a structural analogy and an isoelectronic relationship to purine bases [7]. There are many reports for medicinal potential of thienopyridine derivatives in diverse therapeutical fields, including oncology [8,9,10,11,12], cardiology [13,14], metabolism and endocrinology [15,16], neurology [17,18,19,20], nephrology [21,22], gastroenterology [23], and infectious diseases [24,25,26] (Figure 2). From the agrochemistry viewpoint, thienopyridine derivatives are essential insecticides [27,28]. These works described both the discovery of novel active molecules and the continued investigation of known agents. Moreover, some pharmacologically recognized compounds, such as Ilorasertib and SNX-631, were used for the investigation of pathological processes and the identification of new targets [29,30,31].

In the last decade the synthesis and medicinal application of the thienopyridine core has been the topic of several reviews [32,33,34,35,36,37,38,39,40,41]. However, the most recent review covering all six isomeric forms was published in early 2024. Since that time, considerable advances have been made in both organic and medicinal chemistry of this scaffold, with numerous important contributions reported in the literature. Given our continued focus on pyridine-containing molecular platforms [42,43,44,45,46,47,48], and in line with the current drug discovery program, we herein provide an updated overview of the research landscape in this field. This review highlights the continued interest of the chemical community in thienopyridine and its derivatives incorporated into bioactive molecules, including thienopyridinones and related fused systems such as thienoquinolines, thienoisoquinolines, pyridothienopyrimidines, and others, covering the period from 2024 to the present. The literature search was performed in SciFinder and Reaxys using the substructures of all six isomeric thienopyridines discussed in this review, covering the period from 1 January 2024 to the final search date of 20 August 2026. This search retrieved more than 140 publications, of which approx. 110 were considered. The review is organized into two main parts. The first part is structured according to the synthetic strategy employed: (1) ring construction from various positions and (2) functionalization of preformed thienopyridine rings, for instance, via condensation with pyrimidine. The second part addresses the therapeutic applications of this scaffold, covering infectious diseases (antibacterial, antifungal, and antiviral), cancer therapy, neurological disorders, and metabolic diseases, along with its exploration in agrochemistry.

## 2. Approaches for the Assembly of the Thienopyridine Moiety

**Scheme 1:**
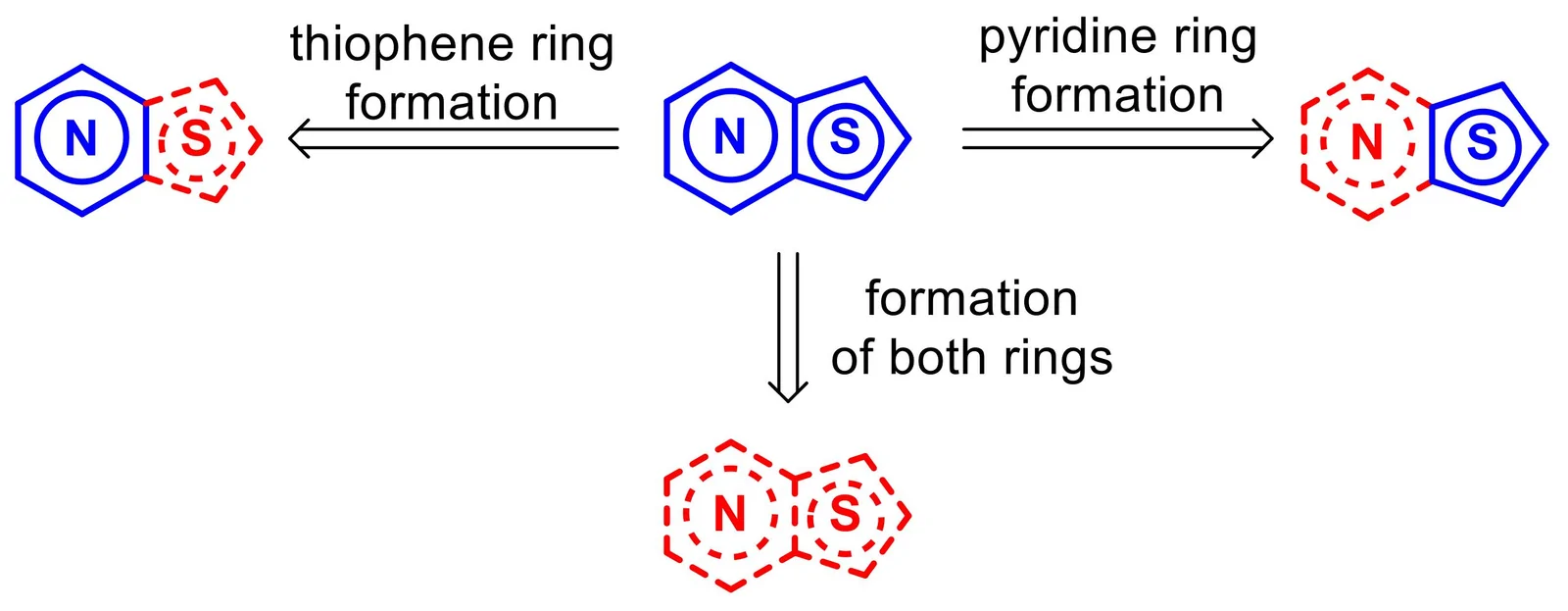
General routes for the assembly of the thienopyridine system.

In general, reactions for the construction of the thienopyridine system are divided into methods based on the closure of the thiophene ring onto a preformed pyridine core (e.g., Thorpe–Ziegler cyclization), methods based on the construction of the pyridine ring onto a preformed thiophene core (Friedländer and Gould–Jacobs-type reactions), and, finally, multicomponent one-step methods that allow the formation of both rings within a single synthetic operation (Scheme 1). A number of recent works demonstrate that all three approaches are currently used.

### 2.1. Thiophene Ring Formation

**Scheme 2:**
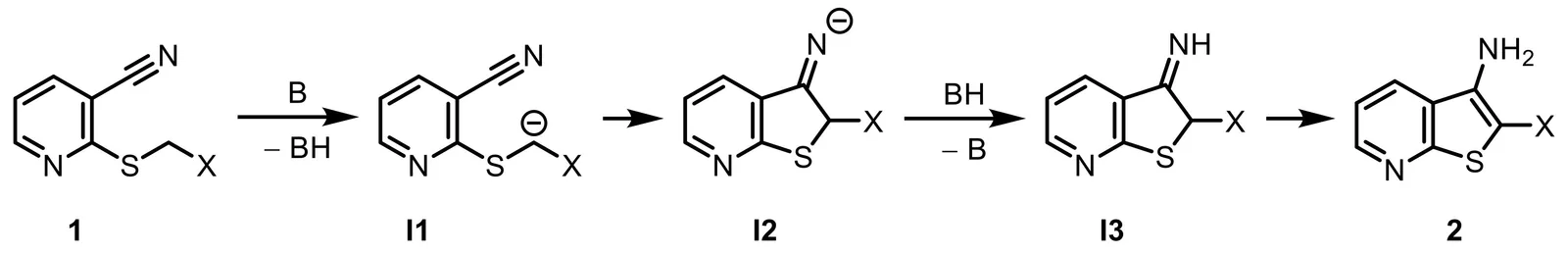
General mechanism of the Thorpe–Ziegler cyclization.

The Thorpe–Ziegler cyclization is the classical and most extensively studied approach for the construction of the thienopyridine system via annulation of a thiophene ring onto a pyridine core. Particularly, the reaction provides common access to thieno [2,3-*b*]pyridine moiety, and the majority of thienopyridine syntheses reported over recent years have employed this transformation. The mechanism of this process (Scheme 2) has been discussed several times and is generally accepted [32,33]. No conceptual innovations in the methodology of this reaction have been introduced during the period under review. The reported examples are mainly associated with the expansion of the substrate scope and the implementation of one-pot protocols that do not require isolation of the S-substituted pyridine-2-thiones **1**. Nevertheless, certain interesting features in the behavior of specific substrates have been described.

**Scheme 3:**
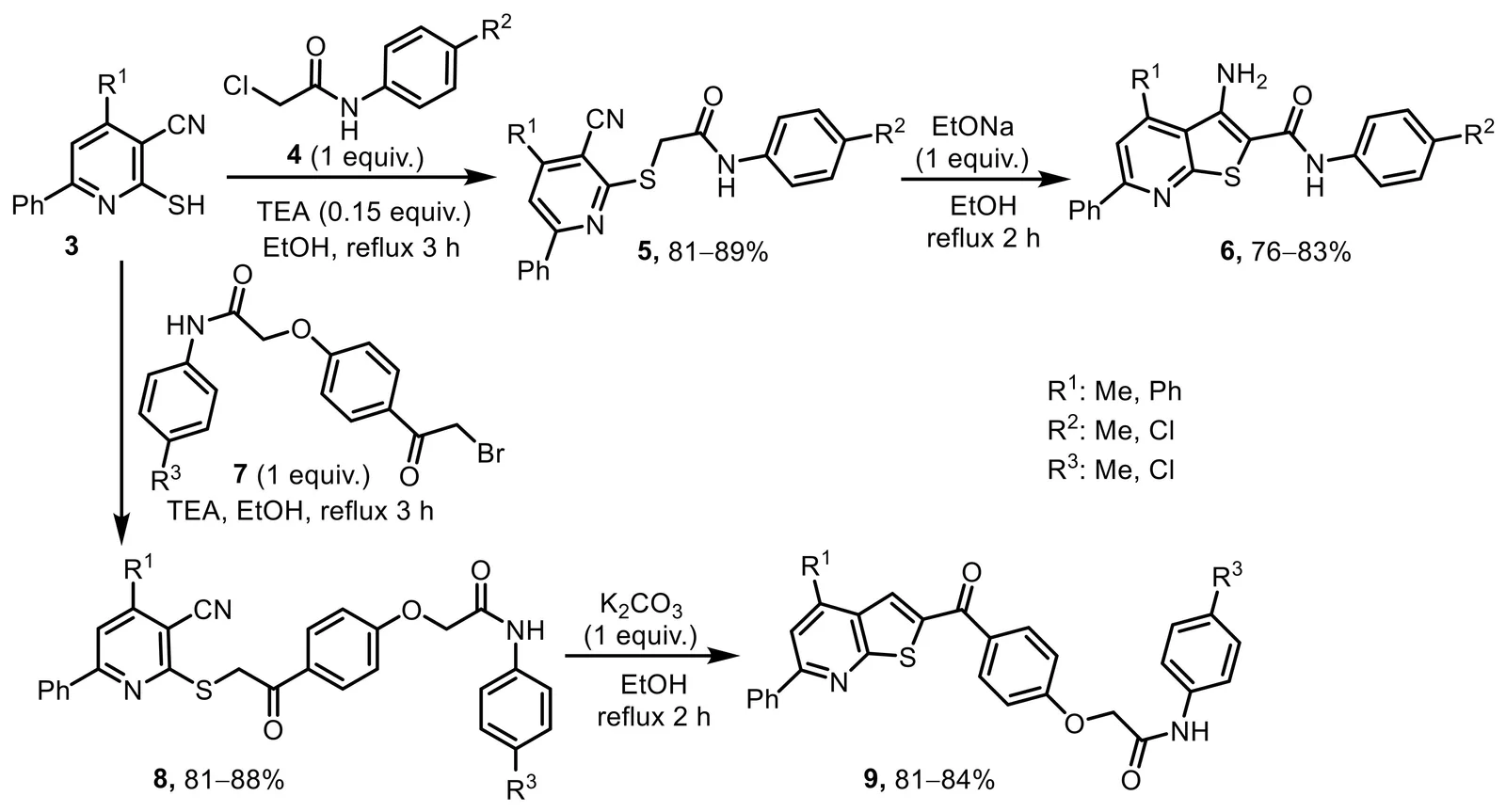
Synthesis of thieno [2,3-*b*]pyridines via S–alkylation followed by Thorpe–Ziegler cyclization.

The typical illustration of the synthesis of thienopyridines via Thorpe–Ziegler heterocyclization is the work of Salem et al. [49]. Readily available 6-phenyl-2-thioxo-1,2-dihydropyridine-3-carbonitriles **3** were employed as key synthetic precursors. They underwent S-alkylation using 2-chloro-*N*-arylacetamides **4** or α-bromoketones **7**. In the first stage, proceeding in the presence of triethylamine (TEA), S-alkylated nicotinonitriles **5** and **8** are formed, which are then cyclized into the target thieno [2,3-*b*]pyridine-2-carboxamides **6** and 2-acylthienopyridines **9** under the action of sodium ethoxide or K_2_CO_3_ (Scheme 3).

**Scheme 4:**
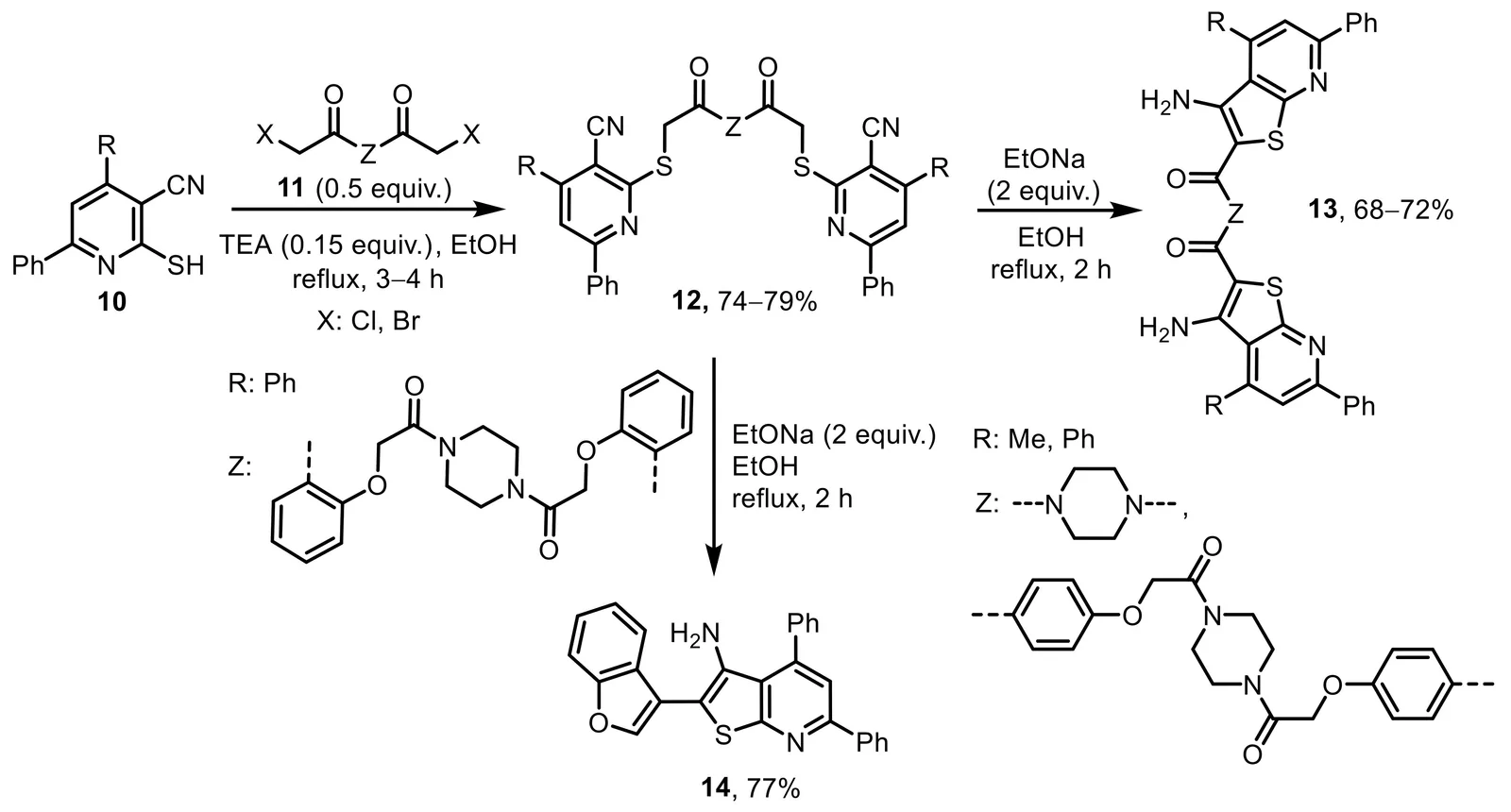
Synthesis of bis-thienopyridine systems.

In this way, not only mono- but also bis-thienopyridines linked through piperazine and phenoxy-acetyl linkers (**13**) were obtained, demonstrating the applicability of the method for the construction of hybrid molecules with two pharmacophoric thienopyridine fragments (Scheme 4). An attempt to obtain bis-thienopyridine by cyclization of bis-sulfide **12** was unsuccessful; instead of the expected product, 2-((benzofuran-3-ylmethyl)thio)-4,6-diphenylnicotinonitrile (**14**) was isolated. The authors explain this result by the competing intramolecular condensation of the methylene group with the ketone followed by hydrolysis and decarboxylation.

**Scheme 5:**
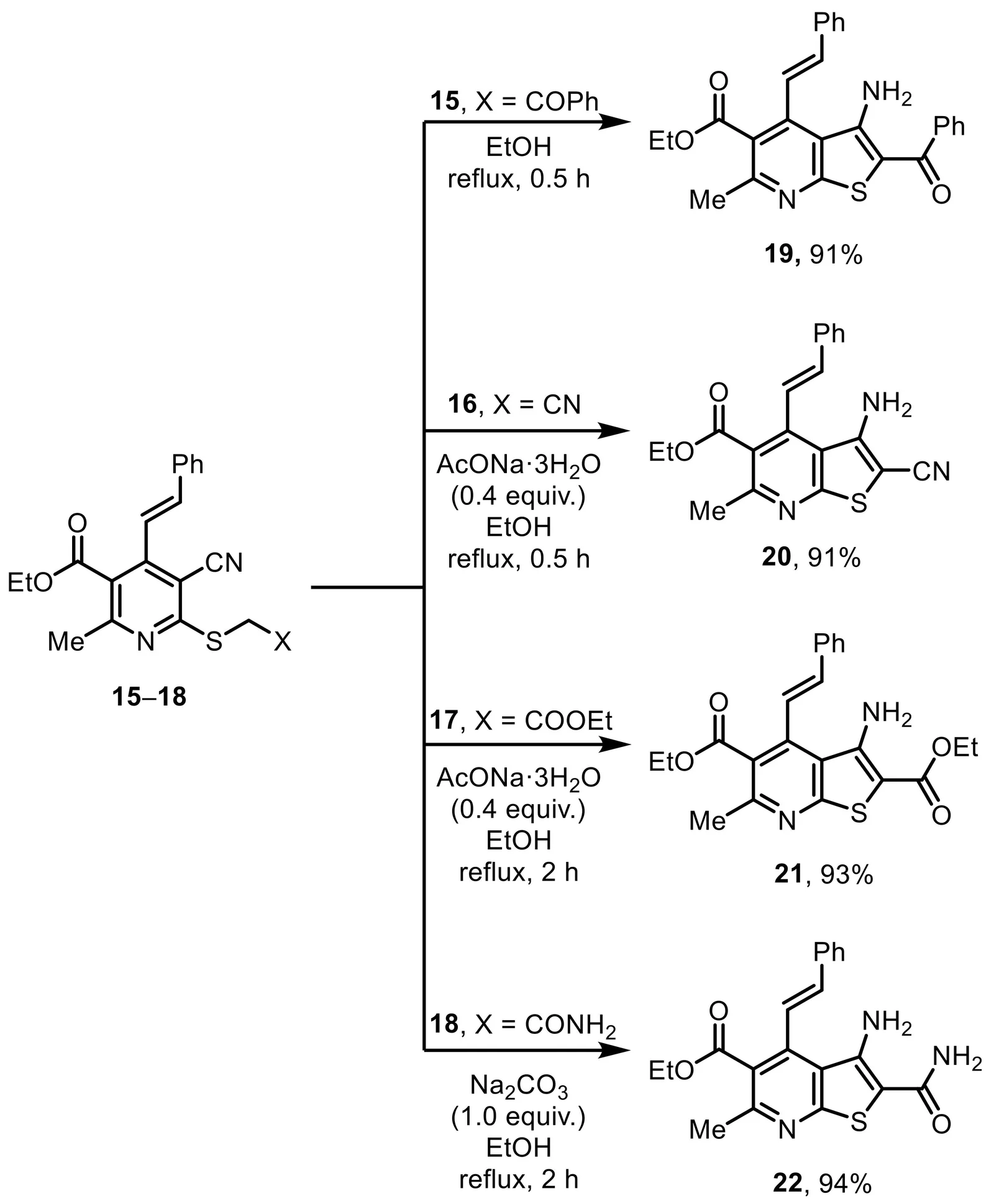
Effect of the electronic nature of the substituent on the Thorpe–Ziegler cyclization.

Al-Waleedy and colleagues [50] demonstrated that the acidity of the CH_2_ moiety in the S-substituted substrate has a significant impact on the rate and conditions of Thorpe–Ziegler cyclization. Particularly, ketone **15** cyclized into thienopyridine **19** upon simple refluxing in ethanol without a catalyst; nitrile **16** and ester **17** required refluxing with a catalytic amount of AcONa (30 min and 2.5 h, respectively); whereas amide **18** cyclized into thienopyridine **22** in the presence of a stronger base–anhydrous Na_2_CO_3_ (Scheme 5).

**Scheme 6:**
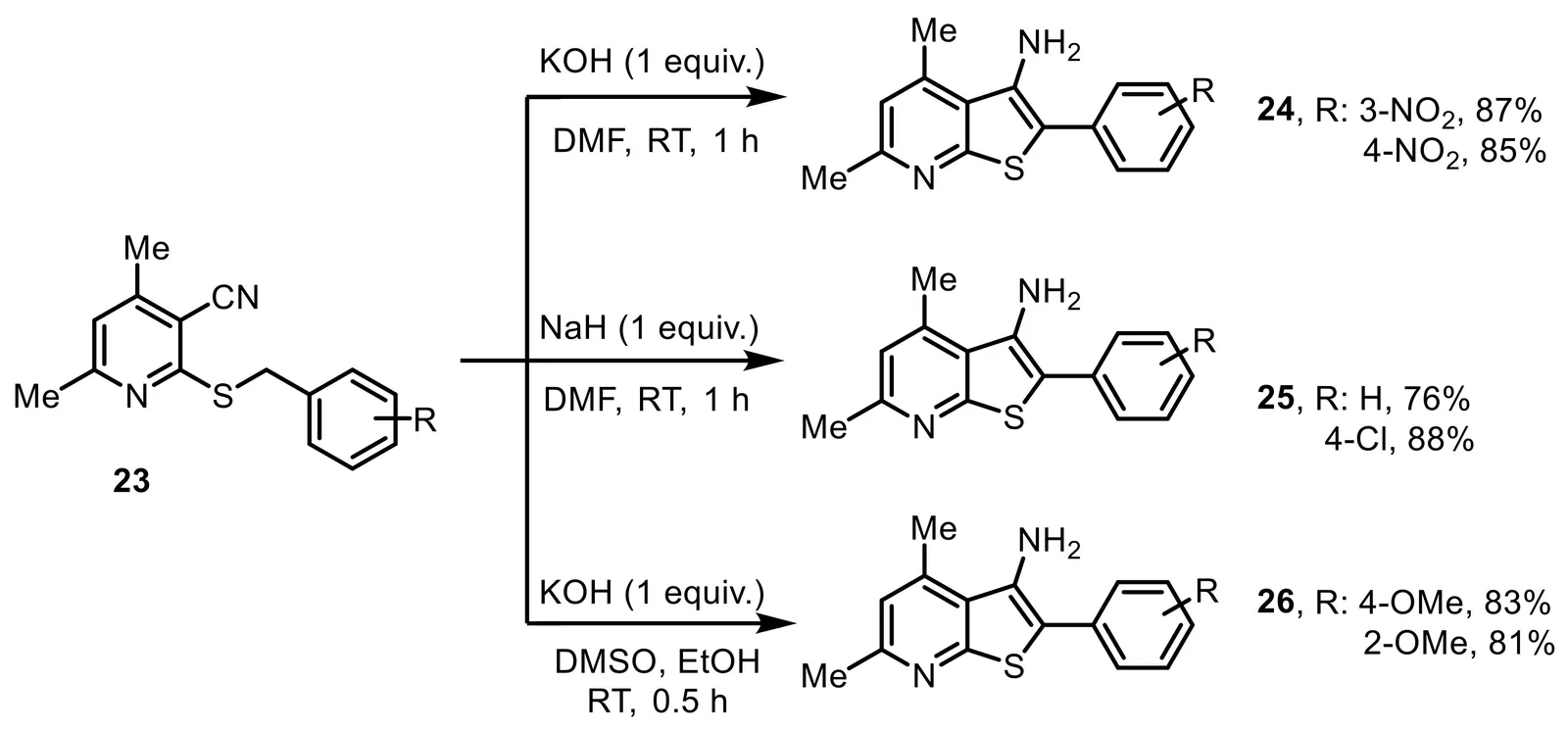
Dependence of Thorpe–Ziegler cyclization conditions on the substituent electronic effects in 2-(benzylthio)-4,6-dimethylnicotinonitriles **23**.

Stroganova and colleagues reported the synthesis of a series of 2-aryl-3-aminothieno [2,3-*b*]pyridines **24**–**26** (Scheme 6) [51]. In this case, cyclization conditions also critically depended on the electronic nature of the substituent in the benzyl fragment. For NO_2_-substituted derivatives (**24**), cyclization proceeded smoothly under the action of aqueous KOH in DMF, whereas unsubstituted and 4-chloro-substituted compounds required the use of NaH in DMF. Attempts to close substrates bearing a strong electron-donating substituent (OMe group) with NaOEt and NaH were unsuccessful, and only the use of the KOH–DMSO system made it possible to obtain the target thienopyridines **26**.

**Scheme 7:**
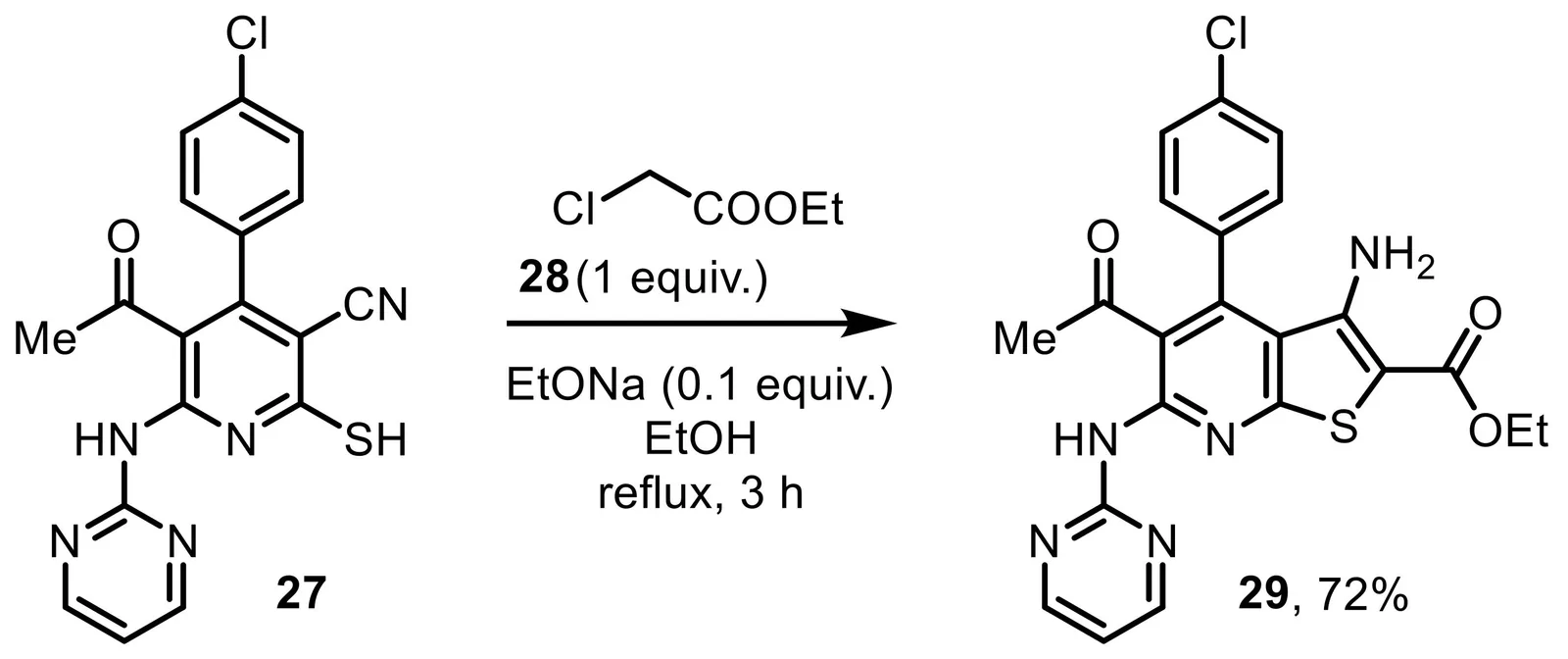
Synthesis of 6-aminopyrimidine-substituted thieno [2,3-*b*]pyridine **29**.

Hussein and colleagues reported the synthesis of 6-aminopyrimidine-substituted thieno [2,3-*b*]pyridine **27** via refluxing S-substituted pyridine-2-thione precursor **28** in NaOEt/EtOH [52] (Scheme 7). The authors suggest that this compound is a potential corrosion inhibitor.

**Scheme 8:**
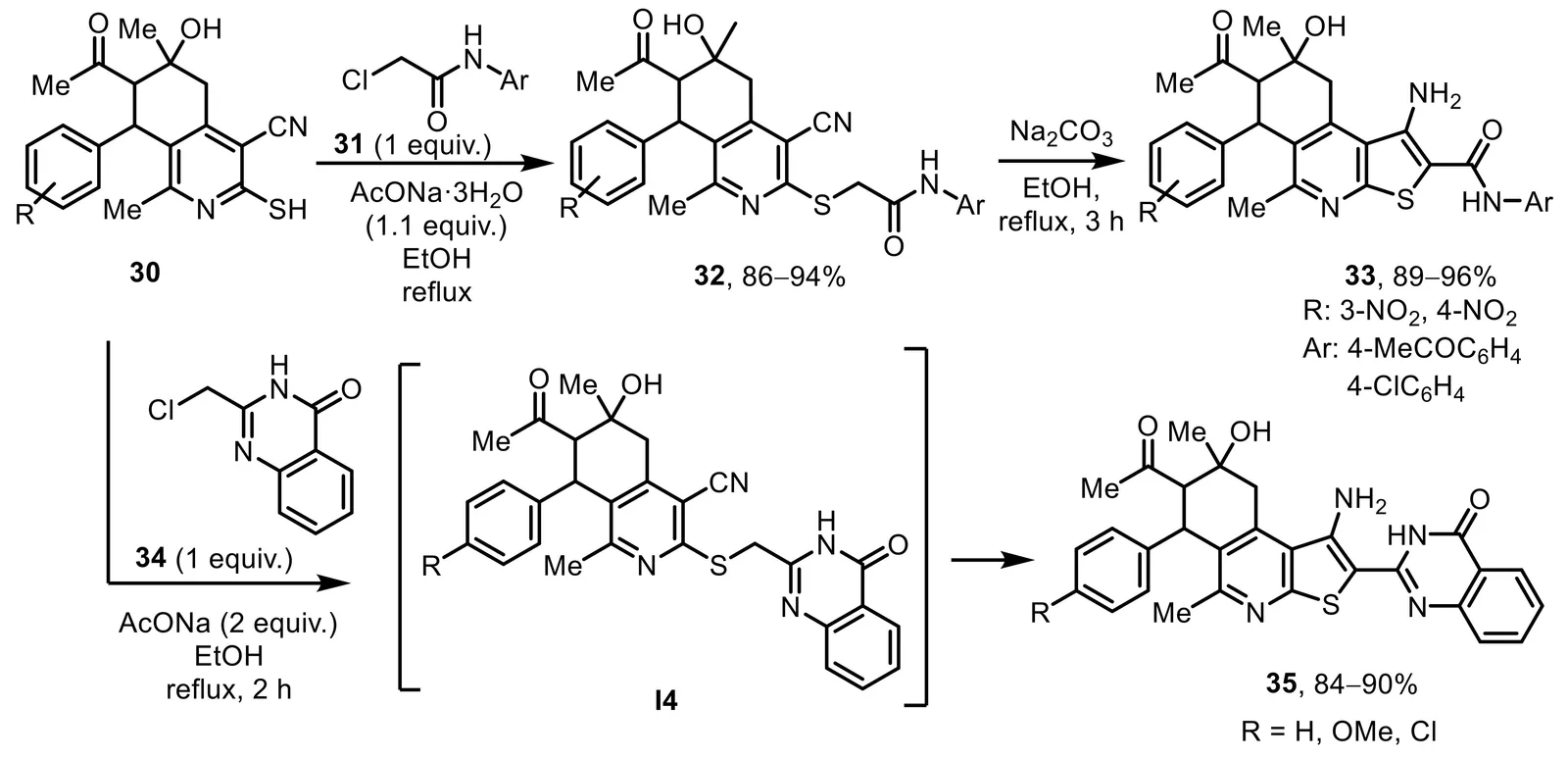
Synthesis of thieno [2,3-*c*]isoquinolines **33** and **35**.

The Thorpe–Ziegler reaction is applicable not only to monocyclic pyridine-2-thiones but also to their isoquinoline analogues, which expands the synthetic availability of thieno [2,3-*c*]isoquinoline systems. For example, Bakhite and colleagues [53,54] synthesized 6,7,8,9-tetrahydrothieno [2,3-*c*]isoquinolines (**33**, **35**) using thiones **30** as substrates for S-alkylation followed by Thorpe–Ziegler cyclization in the presence of sodium carbonate or acetate (Scheme 8).

**Scheme 9:**
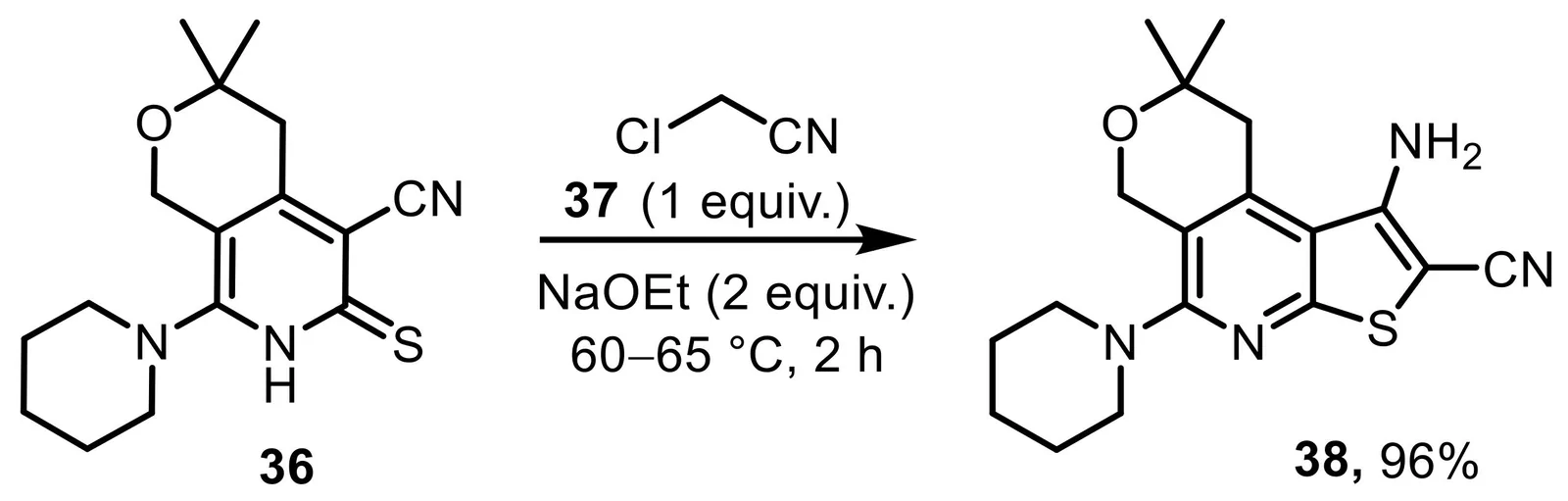
Synthesis of pyrano-fused thieno [2,3-*b*]pyridine **38**.

Another example of the synthesis of polycyclic compounds based on Thorpe–Ziegler cyclization is described by Dashyan et al. [55]. The starting thioxo-pyridinecarbonitrile **36** was reacted with chloroacetonitrile (**37**) in the presence of sodium ethoxide at 60–65 °C to afford the pyrano-fused thieno [2,3-*b*]pyridine **38** in 96% yield via a one-pot Thorpe–Ziegler cyclization without isolation of the S-alkylated intermediate (Scheme 9).

**Scheme 10:**
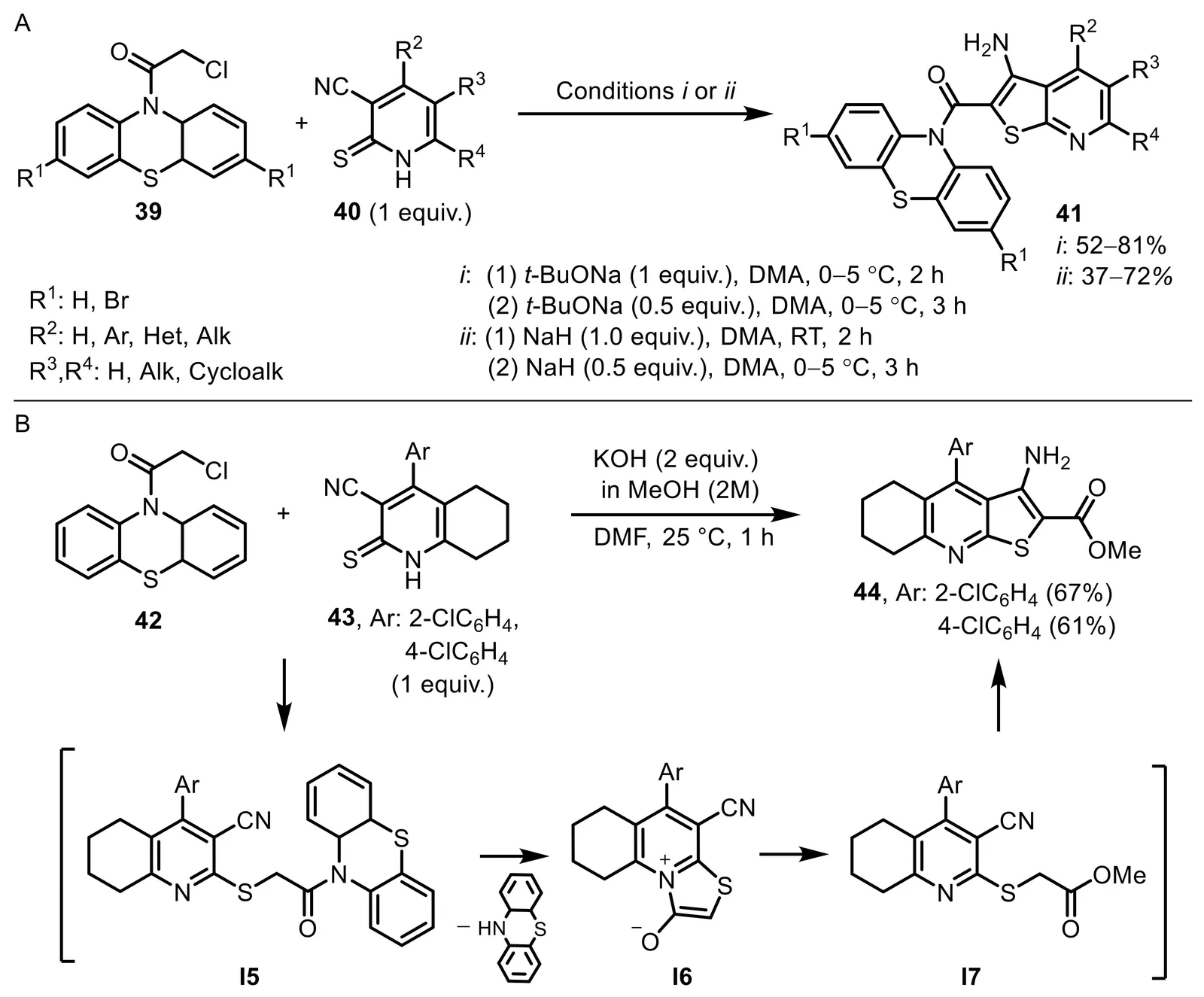
Synthesis of phenothiazine–thienoquinoline heterodimers (**A**,**B**) formation of methyl 3-amino-4-aryl-5,6,7,8-tetrahydrothieno[2,3-*b*]quinoline-2-carboxylates.

Dotsenko and colleagues [56,57] developed a method for the synthesis of phenothiazine/3-aminothieno [2,3-*b*]pyridine heterodimers **41** via the reaction of 10-(chloroacetyl)phenothiazines (**39**) with 2-thiopyridine-3-carbonitriles (**40**) (Scheme 10A). Sodium *tert*-butoxide and NaH were compared as the more suitable bases for the Thorpe–Ziegler cyclization, and in the case of *t*-BuONa the desired products were obtained in higher yields (52–81% for *t*-BuONa vs. 37–72% for NaH). In addition, it was found that the use of KOH/MeOH in DMF for the Thorpe–Ziegler cyclization afforded unsubstituted phenothiazine and methyl 3-amino-4-aryl-5,6,7,8-tetrahydrothieno[2,3-*b*]quinoline-2-carboxylates (**44**) in 61–67% yields instead of the expected heterodimers (Scheme 10B). The authors rationalized this outcome by the formation of a mesoionic intermediate **I6**, wherein the phenothiazine moiety acts as a good leaving group, followed by solvolysis and subsequent Thorpe–Ziegler cyclization of the resulting quinoline intermediates **I7**.

**Scheme 11:**
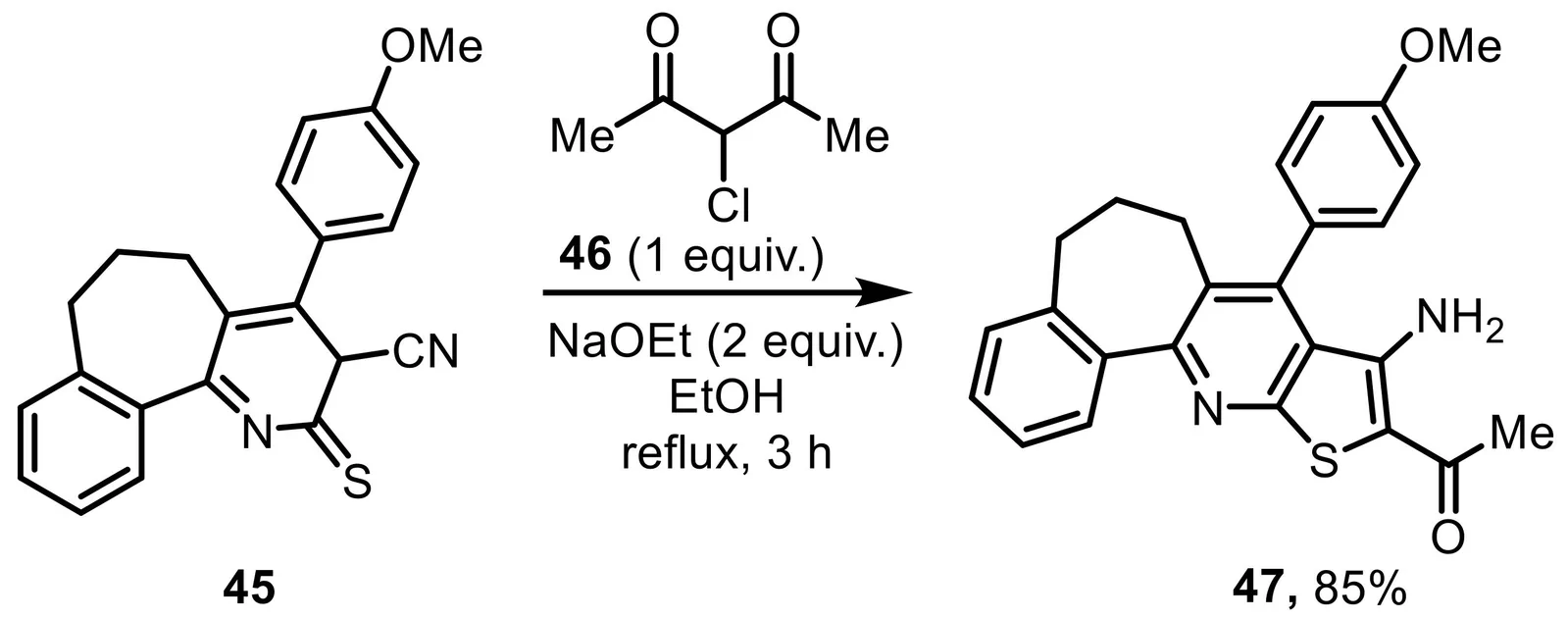
Synthesis of thienopyridine derivative 47 using 3-chloroacetylacetone.

Abdallah et al. [58] described the synthesis of thienopyridine **47** via the reaction of the corresponding pyridine-3-carbonitriles **45** with 3-chloroacetylacetone **46**, followed by the Thorpe–Ziegler cyclization in NaOH/EtOH under reflux (Scheme 11).

**Scheme 12:**
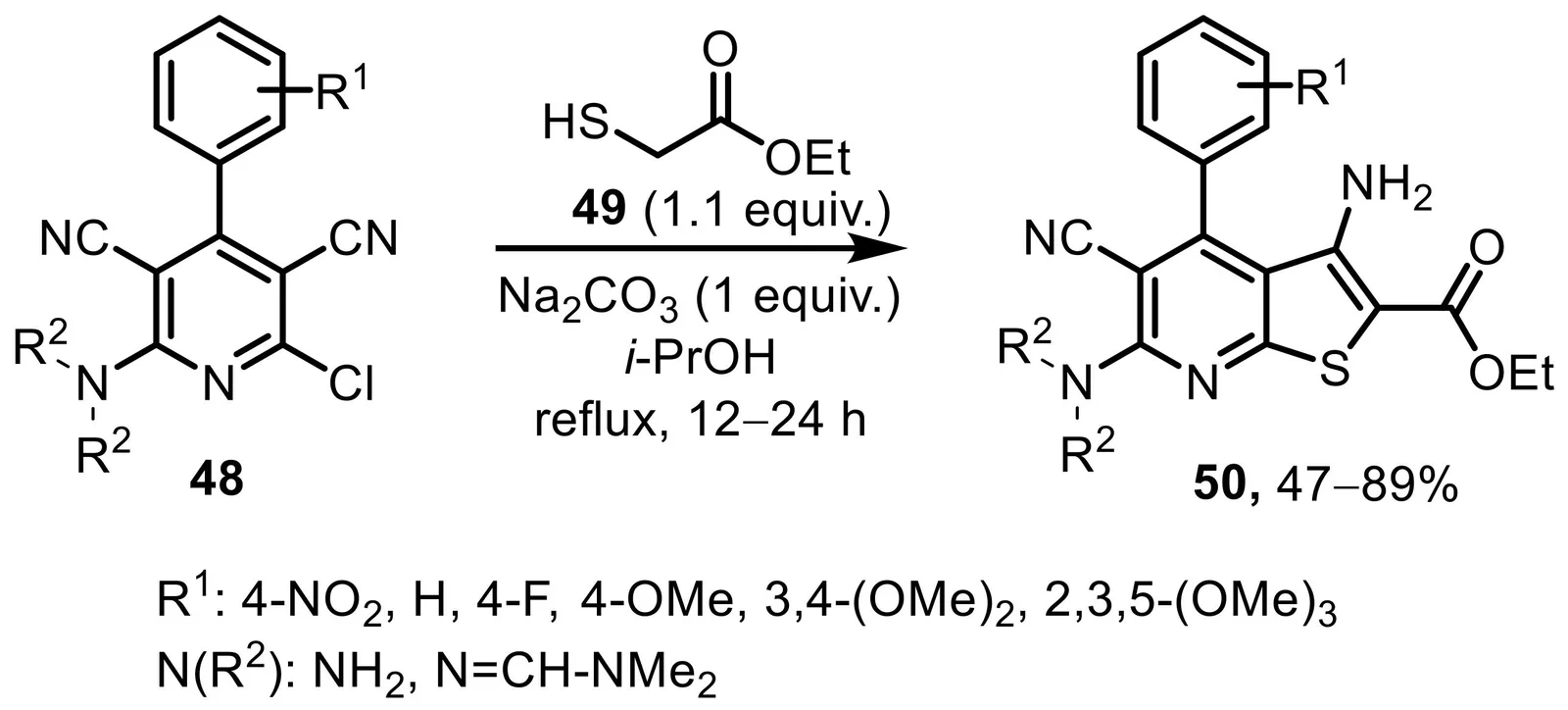
Synthesis of ethyl 3-amino-4-aryl-5-cyanothieno [2,3-*b*]pyridine-2-carboxylates **50**.

Tuktin and colleagues [59,60] reported an alternative synthetic route to thieno [2,3-*b*]pyridines based on nucleophilic aromatic substitution (S_N_A_r_). 4-Aryl-2-amino-6-chloropyridine-3,5-dicarbonitriles **48** were treated with ethyl mercaptoacetate (**49**) in the presence of K_2_CO_3_ in refluxing isopropanol. The reaction proceeded via displacement of the chlorine atom by the thiolate anion, followed by spontaneous intramolecular Thorpe–Ziegler cyclization of the resulting thioether intermediate, directly affording ethyl 3-amino-4-aryl-5-cyanothieno [2,3-*b*]pyridine-2-carboxylates **50** in 47–89% yields (Scheme 12). Beyond 2-chloropyridine-3-carbonitriles, 2-chloropyridine-3-carbaldehydes can used as starting materials [61].

**Scheme 13:**
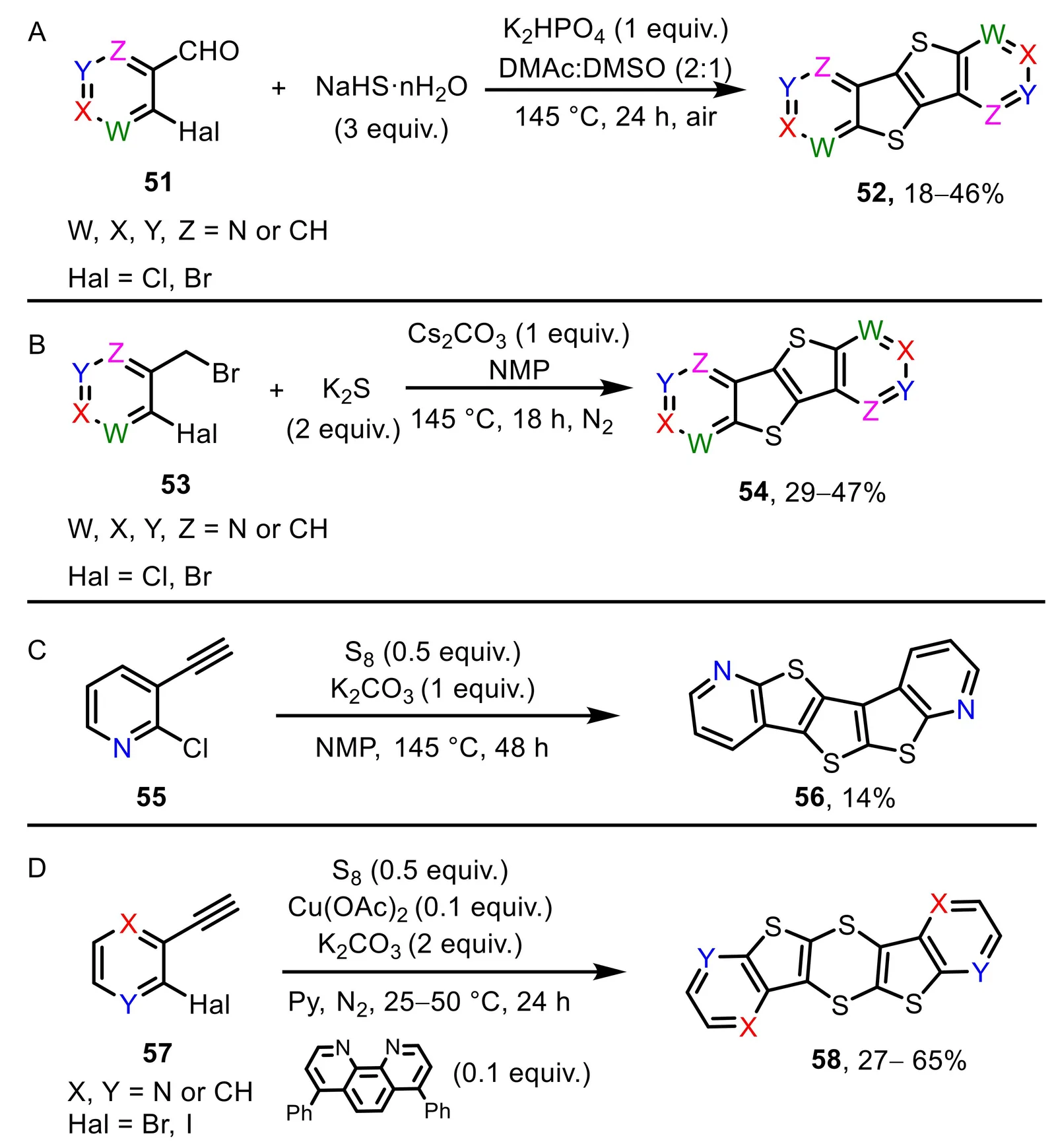
Condensation of halopyridines bearing a functional group at the *ortho* position: (**A**) using NaHS; (**B**) usingh K_2_S; (**C**) using S_8_ in metal-free conditions; (**D**) using S_8_ by Cu(II)-catalyzed reaction.

Although the Thorpe–Ziegler cyclization is the dominant approach, there are some alternative methods of thiophene ring annulation in the course of thienopyridine scaffold synthesis. The most common among these alternatives, and widely investigated in recent years, is the condensation of halopyridines bearing a functional group at the *ortho* position—such as formyl [62], halomethyl [63] or terminal acetylene [64,65]—with an appropriate sulfur atom source (e.g., K_2_S [63], NaHS [62], S_8_ [64,65]). Depending on the starting materials used, either tetra- or pentacyclic products can be achieved. Particularly, in the case of *o*-halonicotinaldehydes **51** or *o*-bromomethylhalopyridines **53**, pyridine analogues of [1]benzothieno [3,2-*b*][1]benzothiophene were formed **52** and **54** (Scheme 13A,B) [62,63], whereas *o*-halopyridylacetylenes **55** and **57** provide the pentacyclic core (Scheme 13C,D). The structure of the latter is controlled by the condensation conditions: metal-free prolonged heating at 145 °C gives the thieno [2,3-*b*;4,5-*b*′]dithiophene moiety **56** [65], while the Cu(II)-catalyzed reaction furnishes thiophene-fused 1,4-dithiin derivatives **58** [64].

**Scheme 14:**
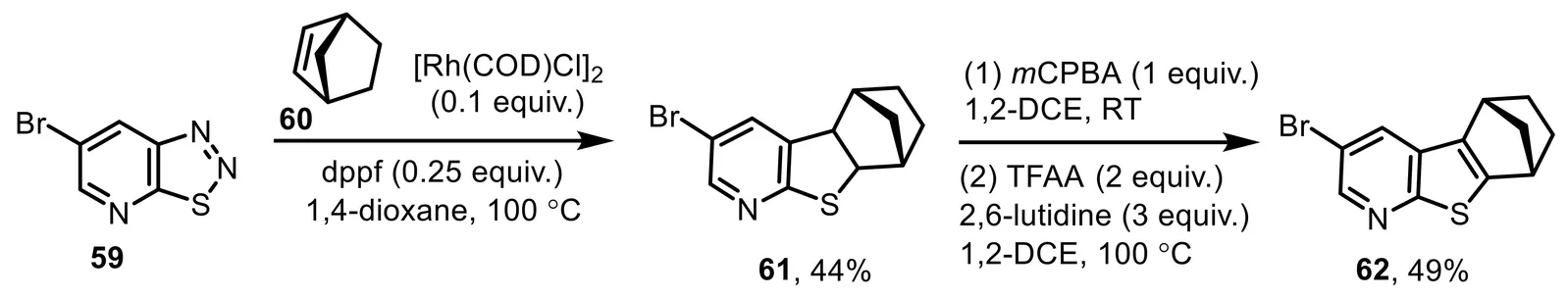
Synthesis of norbornane-fused thienopyridine **62**.

An interesting protocol for the synthesis of thienopyridines **62** fused with a pharmaceutically privileged norbornane moiety was reported by Wappes and colleagues [66]. The reaction proceeds vi a Rh(I)-catalyzed cycloaddition of fused 1,2,3-thiadiazoles **59** with norbornene **60** followed by oxidation using one-pot Pummerer procedure (Scheme 14).

**Scheme 15:**
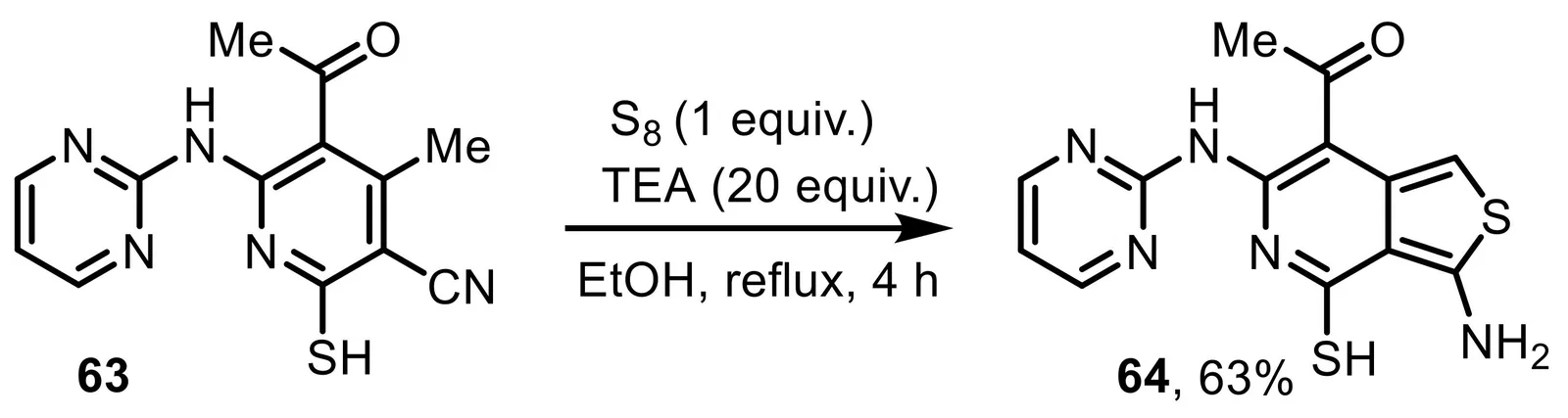
Synthesis of thieno [3,4-*c*]pyridinone **64**.

Moreover, Hussein and colleagues used the Gewald reaction for the synthesis of 1-(3-amino-4-mercapto-6-(pyrimidin-2-ylamino)thieno [3,4-*c*]pyridin-7-yl)ethan-1-one (**64**) (Scheme 15) [52].

### 2.2. Pyridine Ring Formation

In contrast to thiophene ring closure strategies, which have seen little methodological progress in recent years, approaches based on pyridine ring annulation have advanced considerably (for comparison with earlier works on pyridine ring annulation, see the review [32]). In addition to classical methods such as the Friedländer- and Gould–Jacobs-type reactions, many new metal-catalyzed, electrochemical, and photo-induced cyclizations have been developed. Importantly, in most cases, these new methods are directed towards the construction of pyridine-fused heterocycles such as quinolones, isoquinolines, acridines, and phenanthridines, whereas the thienopyridine moiety is considered only as a minor part of the reaction scope.

**Scheme 16:**
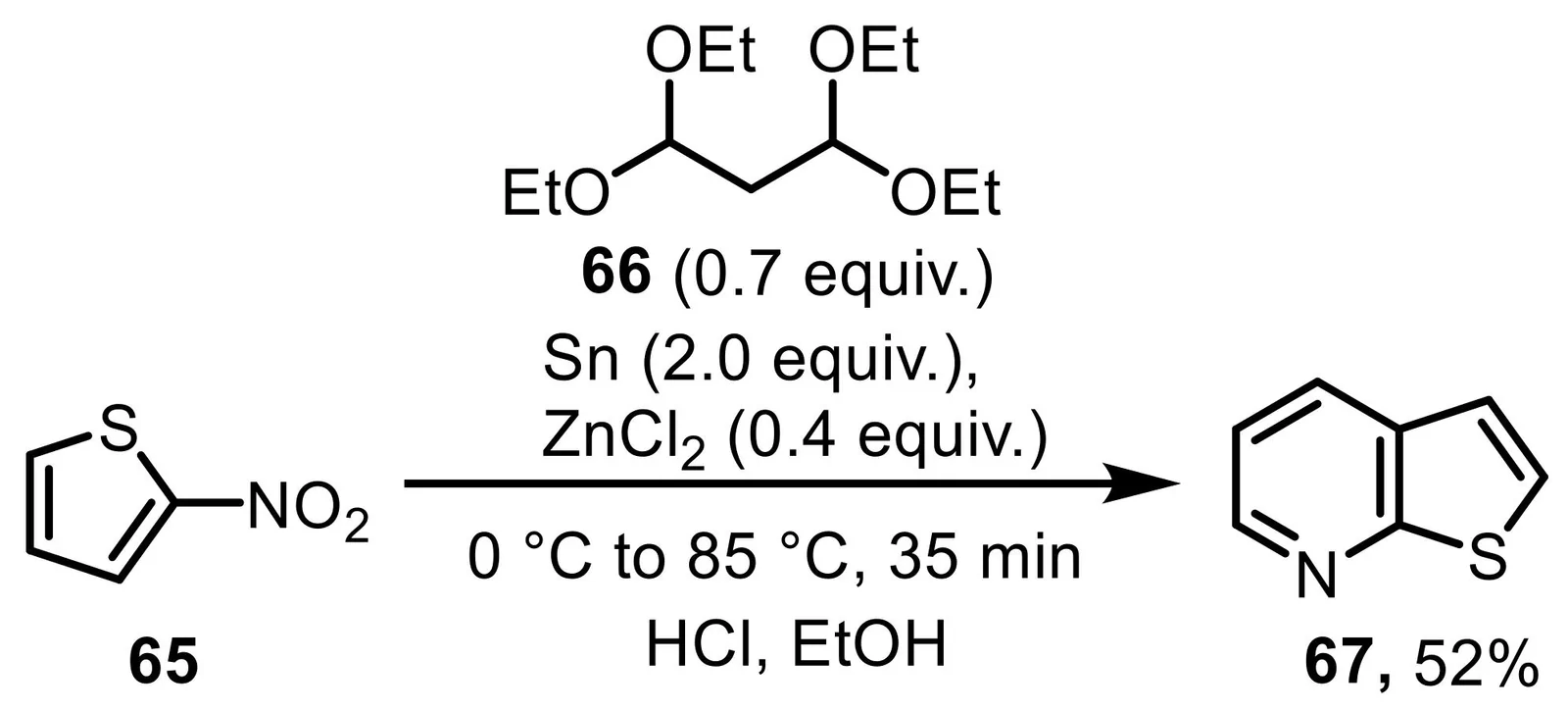
Synthesis of thieno [2,3-*b*]pyridine **67** by the Gould–Jacobs reaction.

A recent example of the application of the Gould–Jacobs reaction for the construction of thieno [2,3-*b*]pyridine core was provided in the work of Xie and colleagues [67]: 2-aminothiophene, obtained in situ from 2-nitrothiophene **65**, was involved in condensation with malonaldehyde diethyl acetal (**66**). The electrophilic attack of the iminium intermediate, generated in acidic medium, on the amino group of thiophene was accompanied by intramolecular 6π-electrocyclization followed by aromatization, which directly led to unsubstituted thieno [2,3-*b*]pyridine (**67**) in 52% yield (Scheme 16).

**Scheme 17:**
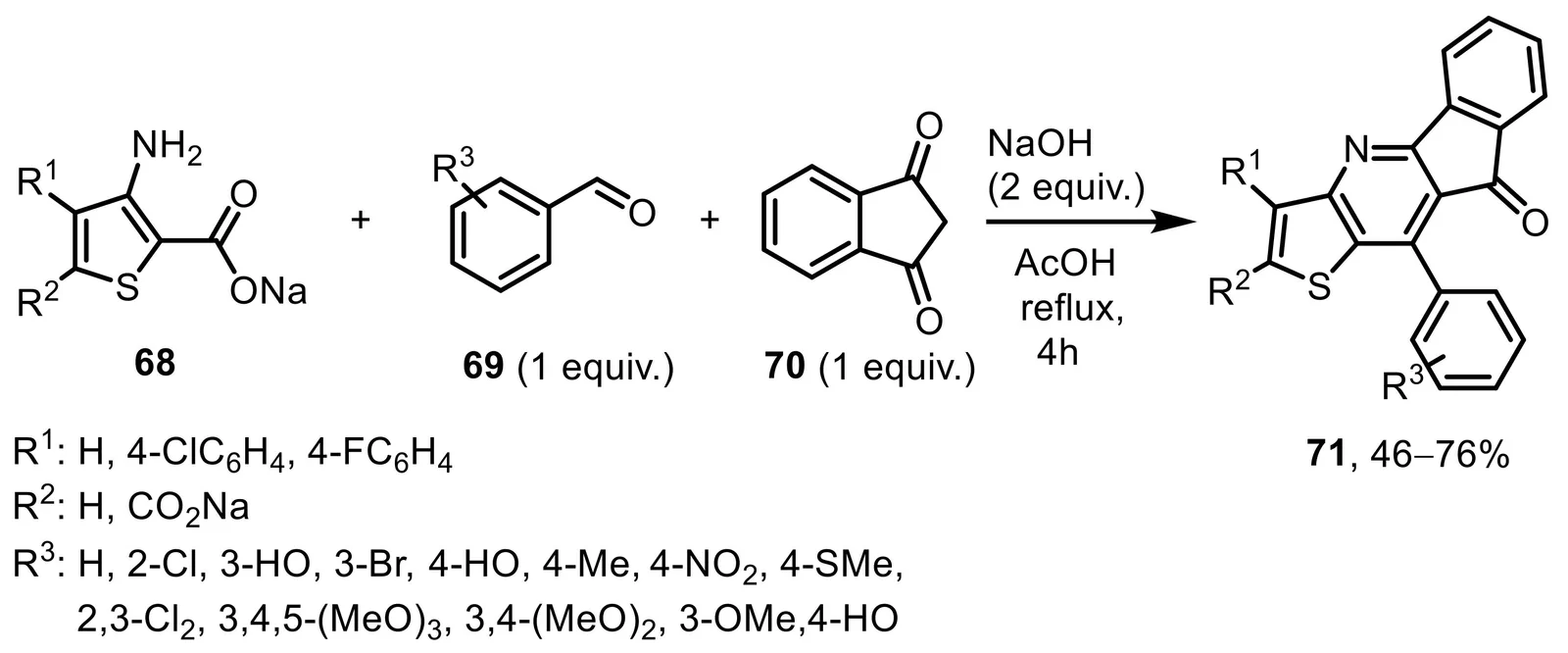
One-pot three-component condensation of aminothiophenes **68**, aromatic aldehydes **69**, and 1,3-indandione **70**.

One more example of the Gould–Jacobs reaction for the construction of the thienopyridine (in this case, for a [3,2-*b*]-isomer) system was reported by Kudryavtseva and colleagues [68]. They developed a protocol for a one-pot three-component condensation of aminothiophenes **68** with various aromatic aldehydes **69** and 1,3-indandione **70** in acetic acid at reflux (Scheme 17). The authors used 3-aminothiophene-2-carboxylates as precursors for the in situ generation of 2-unsubstituted 3-aminothiophene. The reaction scope includes 21 new 9*H*-indeno [1,2-*b*]thieno [2,3-*e*]pyridin-9-ones **71** in yields of 46–76%.

**Scheme 18:**
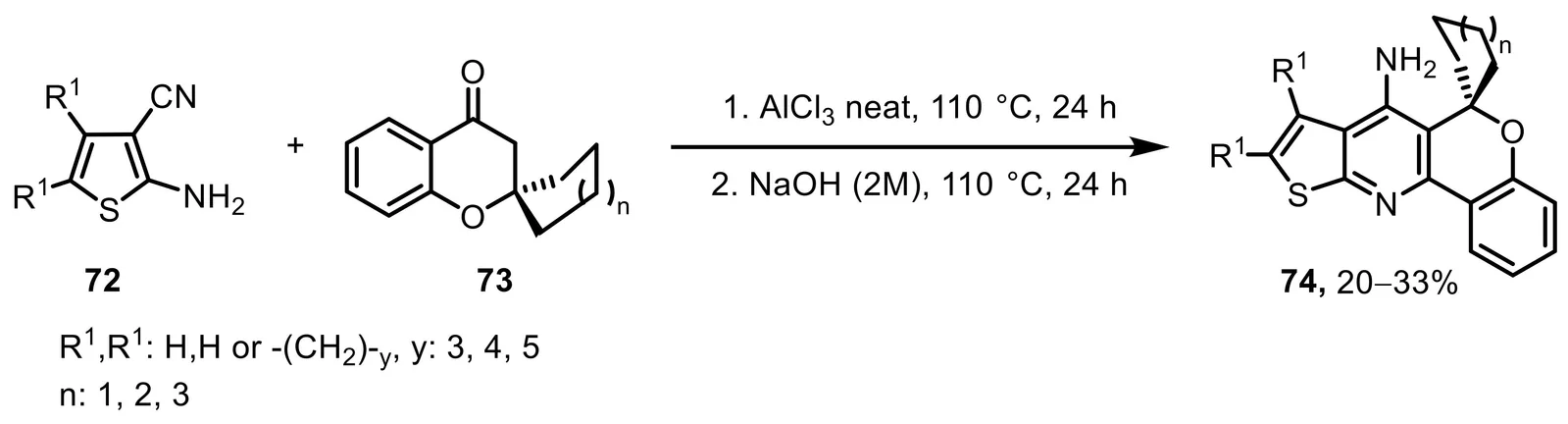
Synthesis of spiro[chromeno [4,3-*b*]thieno [3,2-*e*]pyridine]-7-amines.

The formation of the thieno [2,3-*b*]pyridine system based on the Friedländer reaction was reported by Kappenberg et al. [69]. They synthesized spiro[chromeno [4,3-*b*]thieno [3,2-*e*]pyridine-6,1′-cycloalkane]-7-amines **74** from carbonitriles **72** and spiro[chroman-2,1′-cycloalkane]-4-ones **73** in the pretense of AlCl_3_ as catalyst (Scheme 18).

**Scheme 19:**
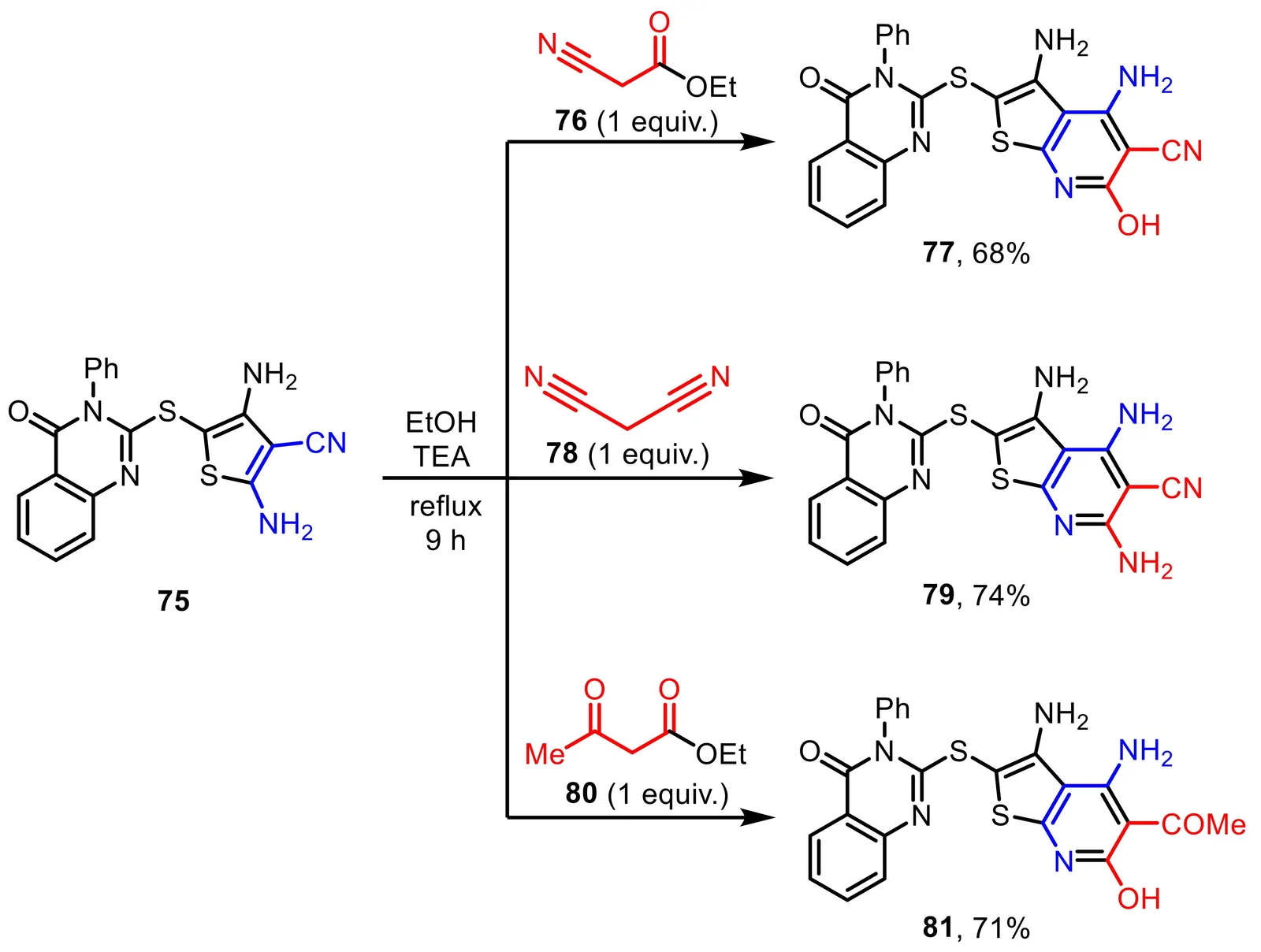
Synthesis of potential p38α MAPK inhibitors via Friedländer-type cyclocondensation.

Alsfouk and colleagues [70] employed a Friedländer-type cyclocondensation for the synthesis of thieno [2,3-*b*]pyridine derivatives as potential p38α mitogen-activated protein kinase (MAPK) inhibitors. The 2,4-diaminothiophene-3-carbonitrile **75** was treated with ethyl cyanoacetate (**76**), malononitrile (**78**), or ethyl acetoacetate (**80**) in ethanol with triethylamine as a catalyst, giving thieno [2,3-*b*]pyridine-5-carbonitriles **77**, **79**, **81** in 68–74% yields (Scheme 19). However, these derivatives showed only weak antiproliferative activity and were not pursued for further biological evaluation.

**Scheme 20:**
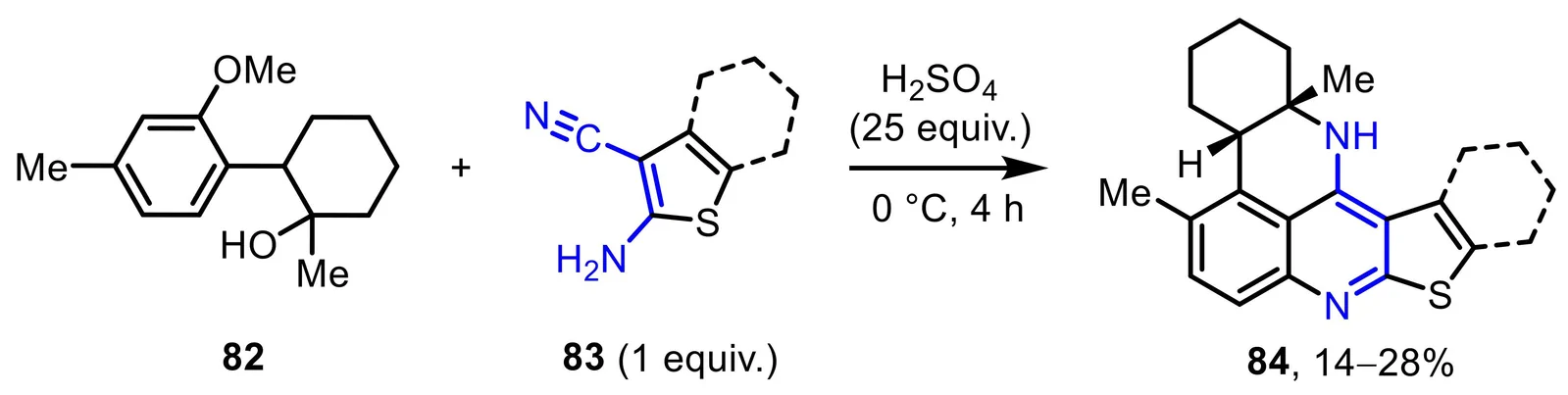
Acid-catalyzed condensation of 1-methylcyclohexan-1-ol **82** with 2-aminothiophene-3-carbonitriles **83**.

Another approach to the assembly of the thieno [2,3-*b*]pyridine system from 2-aminothiophenes was described by Rozhkova and colleagues [71]. The authors developed a protocol for acid-catalyzed condensation between 2-(2-methoxyphenyl)-substituted cycloalkanols **82** with *ortho*-aminonitriles **83**, which enabled the diastereoselective formation of polycyclic heterocycles, including thieno [2,3-*b*]pyridine derivatives **84** (Scheme 20). However, the latter were obtained in poor yields (14–28%).

**Scheme 21:**
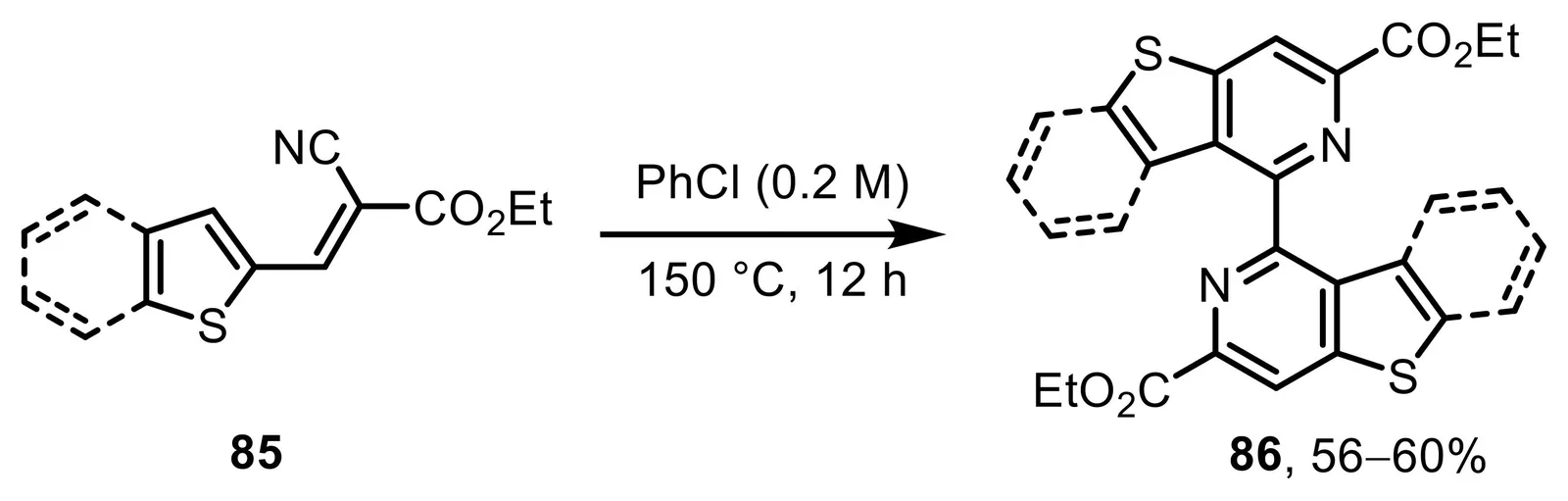
Synthesis of thienopyridine derivatives via thermal dimerization of vinyl isonitriles.

Liu and colleagues [72] reported a metal-free thermal dimerization of vinyl isonitriles **85**, which proceeds via a “head-to-head” cross-coupling followed by 6π-electrocyclization (Scheme 21). Although this methodology was primarily developed for the synthesis of indole derivatives, it was also successfully applied to the construction of two thieno [3,2-*c*]pyridines (**86**), which were obtained in moderate yields. Other examples of the pyridine ring annulation using isocyanide precursor were described in works [73,74,75,76].

**Scheme 22:**
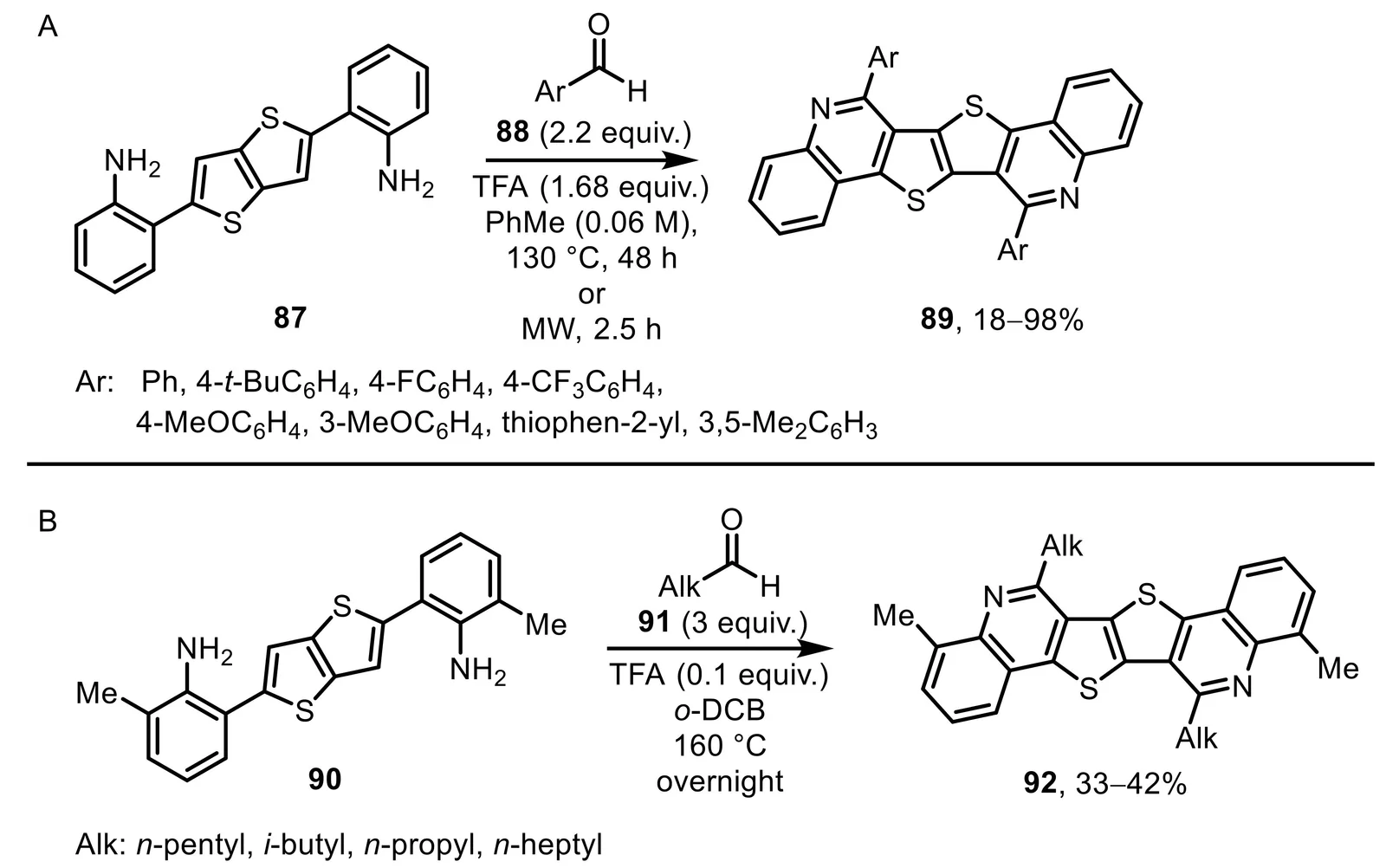
Reaction of bis(2-aminoaryl)thieno [3,2-b]thiophenes with aromatic (**A**) and aliphatic (**B**) aldehydes.

Gao and colleagues [77] reported a two-step synthesis of thieno [3,2-*b*]thiophene-fused bisquinolines **89** via Pictet–Spengler reaction of bis(2-aminoaryl)thieno [3,2-*b*]thiophenes **87** with aldehydes **88** (Scheme 22A). While aromatic aldehydes gave the desired hexacyclic products **89** in moderate to good yields independent of the thieno [3,2-*b*]thiophene substrate structure, aliphatic aldehydes **91** required blocking of the α-position of the aminoaryl moiety to obtain the cyclized products **92** (Scheme 22B). Notably, a similar cyclization for the synthesis of benzobisthio [3,2-*c*]quinolines was reported by Hüßler and colleagues [78].

**Scheme 23:**
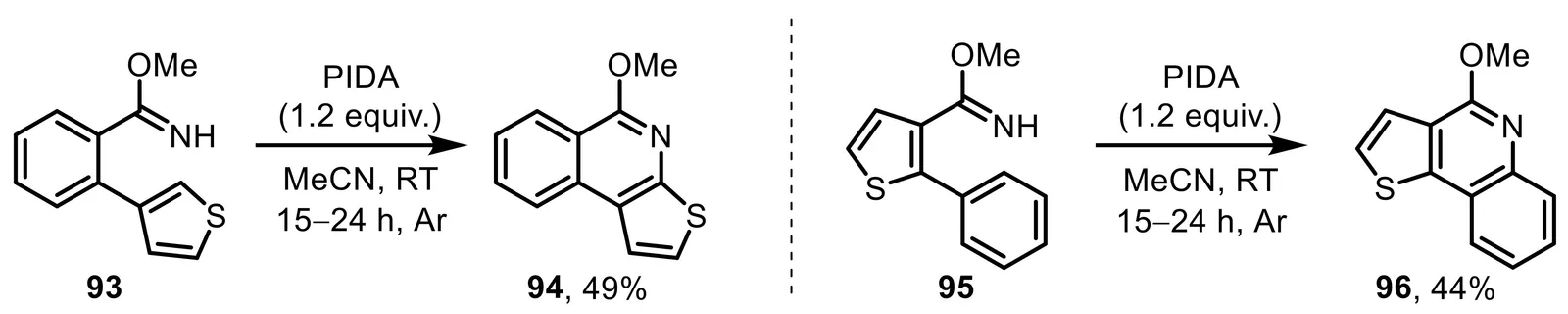
Synthesis of thienopyridine derivatives via PIDA-mediated intramolecular cyclization of 2-substituted-arylcarbimidates **93**, **95**.

Pathare and Selvakumar developed a metal-free method for the synthesis of *N*-heterocycles via PIDA-mediated intramolecular cyclization of 2-substituted-arylcarbimidates into phenanthridines, isoquinolines, quinolines, and pyridines at room temperature (Scheme 23) [79]. The protocol was successfully extended to thiophene-containing substrates **93** and **95**, delivering thieno [2,3-*c*]pyridine **94** (53%) and thieno [3,2-*c*]pyridine **96** (44%) as representatives of two different isomeric thienopyridines. The similar PIDA-mediated cyclizations were also described for 2--alkynyl arylimidates [80] and benzenesulfonamides [81].

**Scheme 24:**
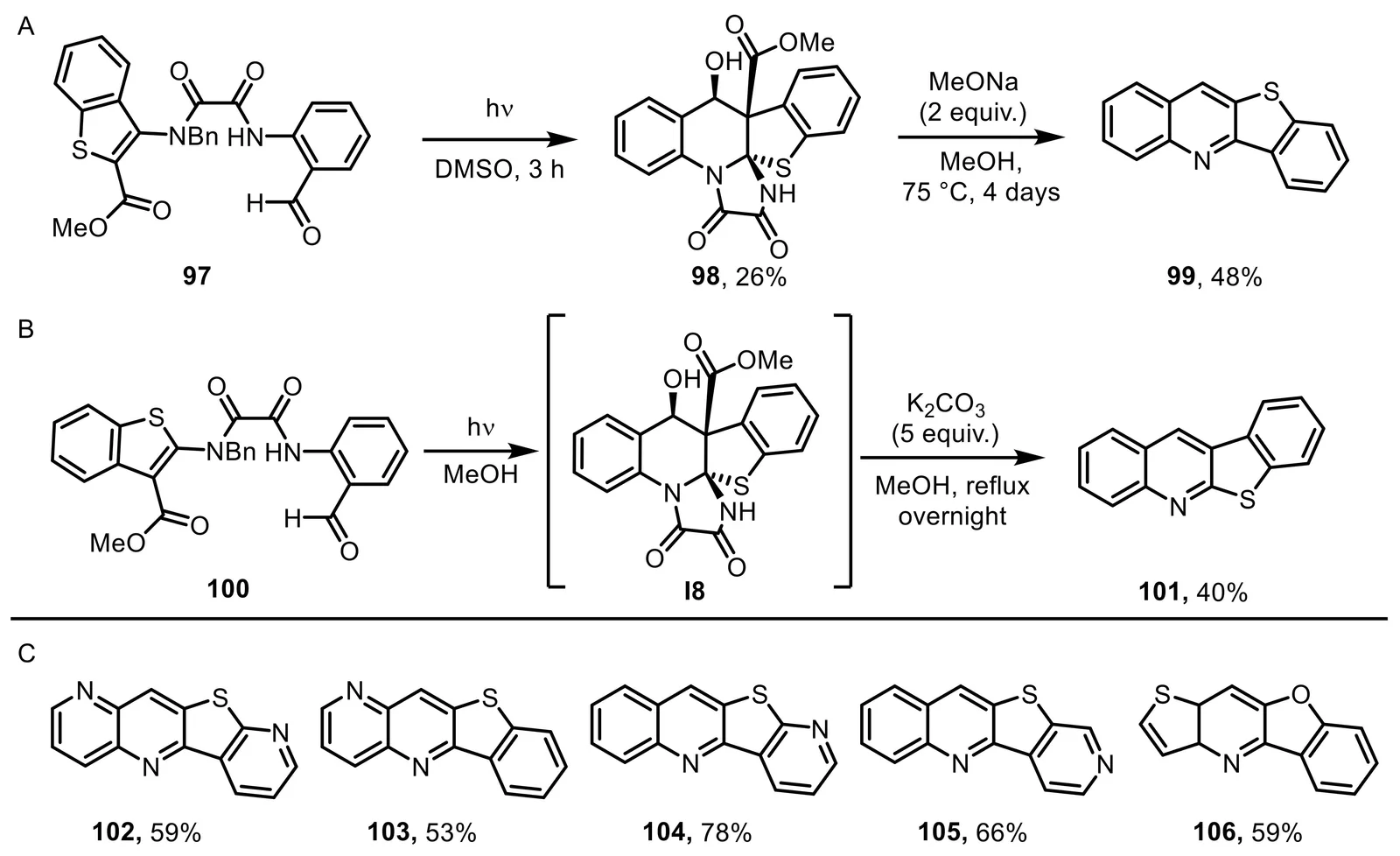
Photochemical intramolecular heterocyclization of diaryl oxalyldiamides in: thieno [3,2-*b*]pyridine (**A**) or thieno [2,3-*b*]pyridine (**B**) derivatives; scope of obtained thienopyridines (**C**).

Not only thermo-initiated heterocyclizations are utilized for the thienopyridine moiety synthesis, photoinduced transformations are also known. For example, the Kutateladze group [82] reported a photochemical intramolecular heterocyclization of diaryl oxalyldiamides followed by the decarboxylative aromatization, which provides access to diverse *N*-polyheterocycles (Scheme 24). Using 2- or 3-aminothiophene bearing substrates **97** and **100**, authors successfully accessed thieno [3,2-*b*]pyridine (Scheme 24A) or thieno [2,3-*b*]pyridine (Scheme 24B) derivatives **99** and **101**. Unlike classical thermal methods, the regioselectivity of this approach is governed exclusively by the geometry of the linker rather than by the electronic properties of the substrates, enabling predictable access to both regioisomeric products. Five polyheterocycles (**102**–**106**) bearing thienopyridine system were successfully synthesized by this method (Scheme 24C).

**Scheme 25:**
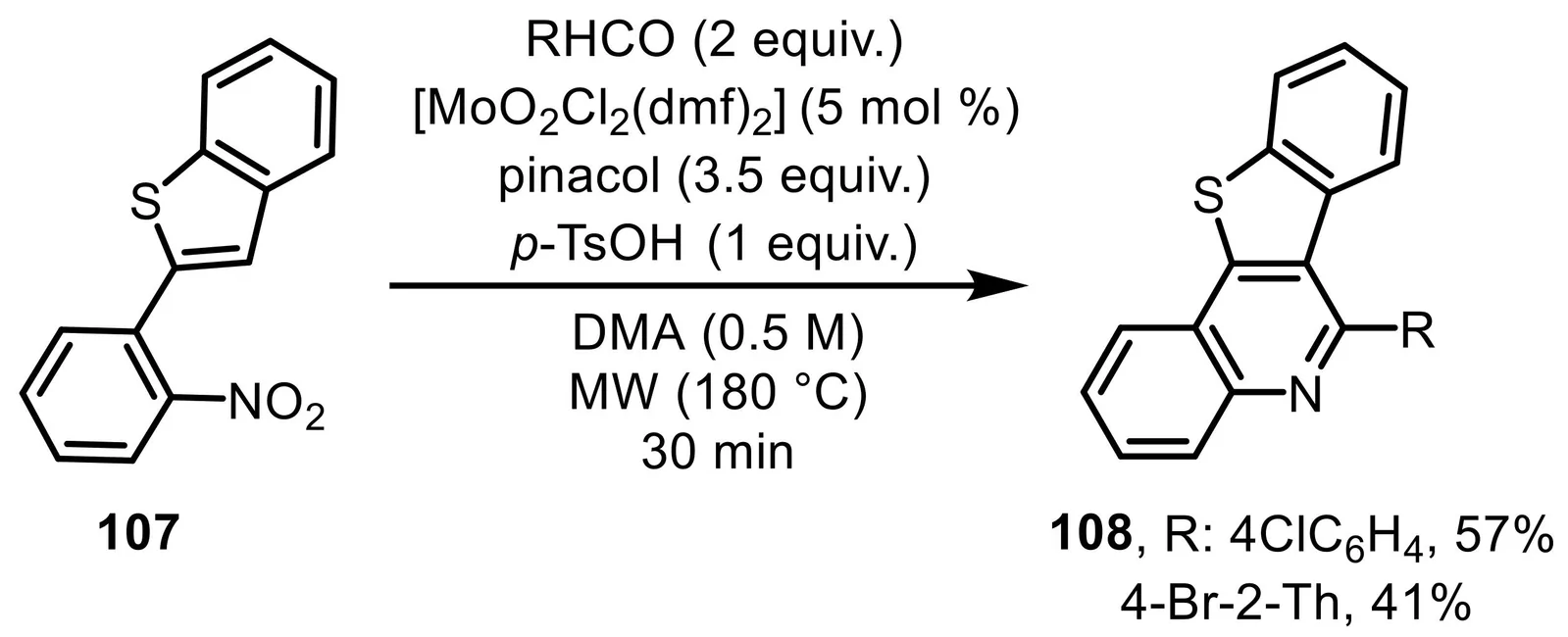
Synthesis of thienopyridine derivatives 106 via Mo(VI)-catalyzed condensation of nitroarenes and aldehydes.

Besides metal-free approaches, many protocols for the synthesis of thienopyridine systems by the metal-catalyzed pyridine ring assembly have been described in the past three years. The major part of them are reactions of metal-catalyzed C–H activation of functionalized aromatic compounds with alkynes [83,84,85,86,87,88,89,90], alkenes [91,92,93,94,95], allenes [96], cyclic 1,3-diones [97], sulfoxonium [98,99] and iodonium ylides [100]. In addition, there are several examples of metal-catalyzed condensations of *o*-substituted anilines with diverse partners [101,102,103]. Catalysts employed include Rh(III) [83,87,91,96], Ir(III) [84,86], Co(III) [85,88,92], Ru(II) [94,99], Ni(II) [102], and Mo(VI) [103] derivatives. For example, Martínez-González and colleagues developed a catalyzed by [MoO_2_Cl_2_(dmf)_2_] tandem process for the synthesis of fused N-polyheterocycles from functionalized nitroarenes and aldehydes [103]. The reaction includes reduction of the nitro group by pinacol as a green reductant, imine formation, and intramolecular cyclization. The protocol was successfully extended to the synthesis of benzothieno [3,2-*c*]quinolines **108** from 2-(2-nitroaryl)benzo[*b*]thiophenes **107** (Scheme 25).

**Scheme 26:**
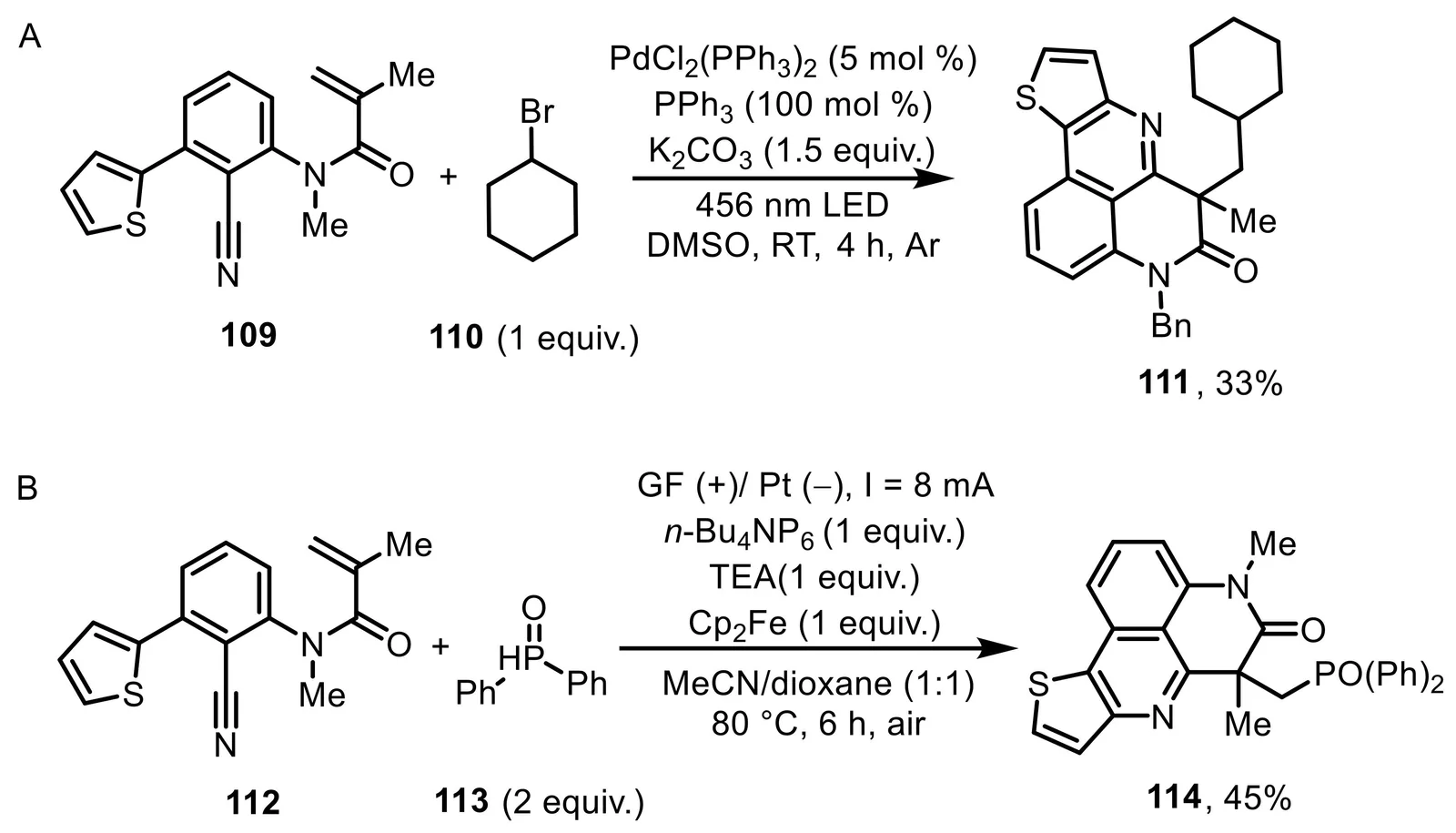
Synthesis of thienopyridine derivatives via cyclization of methacrylamides of *o*-cyanoanilines by (**A**) photoinduced, palladium-catalyzed reaction and (**B**) electrochemical variant.

Khanna and colleagues [104] developed a photoinduced, palladium-catalyzed alkylative cascade cyclization of *o*-cyano methacrylamides with unactivated alkyl halides affording phenanthridines via a radical addition/cyclization pathway under mild conditions. This methodology was successfully applied to the synthesis of thiophene-fused phenanthridine **111**, which was obtained in 33% yield from the corresponding thiophene-containing methacrylamide precursor **109** (Scheme 26A). Furthermore, Ran and colleagues developed an electrochemical variant of such cyclization, employing Cp_2_Fe as a redox mediator and disubstituted phosphine oxides **113** as radical precursors, and obtained thienopyridine derivative **114** in 68% yield (Scheme 26B) [105].

**Scheme 27:**
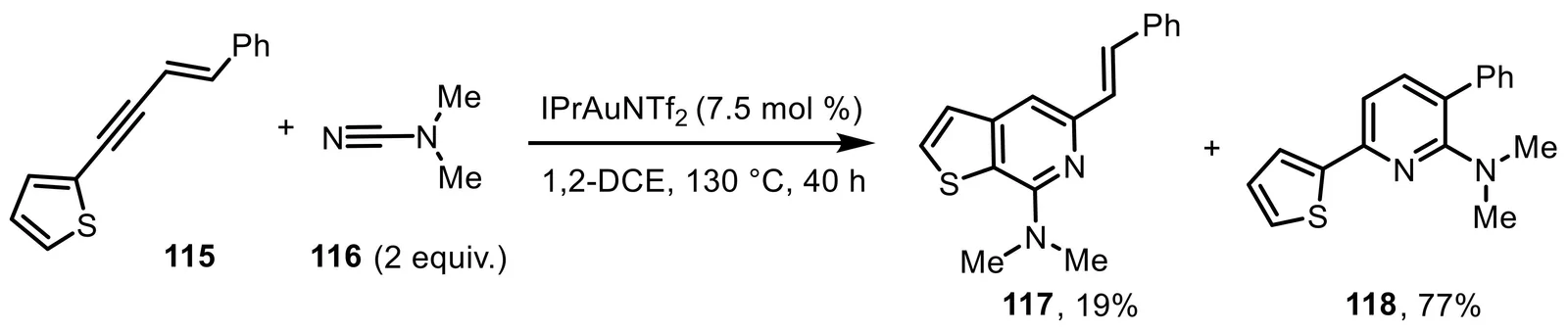
Gold(I)-catalyzed cycloaddition of 2-(4-phenylbut-3-en-1-yn-1-yl)thiophene with dimethylcyanamide.

Moreover, Dubovtsev and colleagues [106] described the formation of *N*,*N*-dimethyl-6-styrylthieno [3,2-c]pyridin-4-amine (**117**) during gold(I)-catalyzed cycloaddition of 2-(4-phenylbut-3-en-1-yn-1-yl)thiophene **115** with dimethylcyanamide **116** (Scheme 27). However, it was isolated in only 14% yield, whereas the main product of this reaction was *N,N*-dimethyl-3-phenyl-6-(thiophen-2-yl)pyridine-2-amine **118**.

**Scheme 28:**
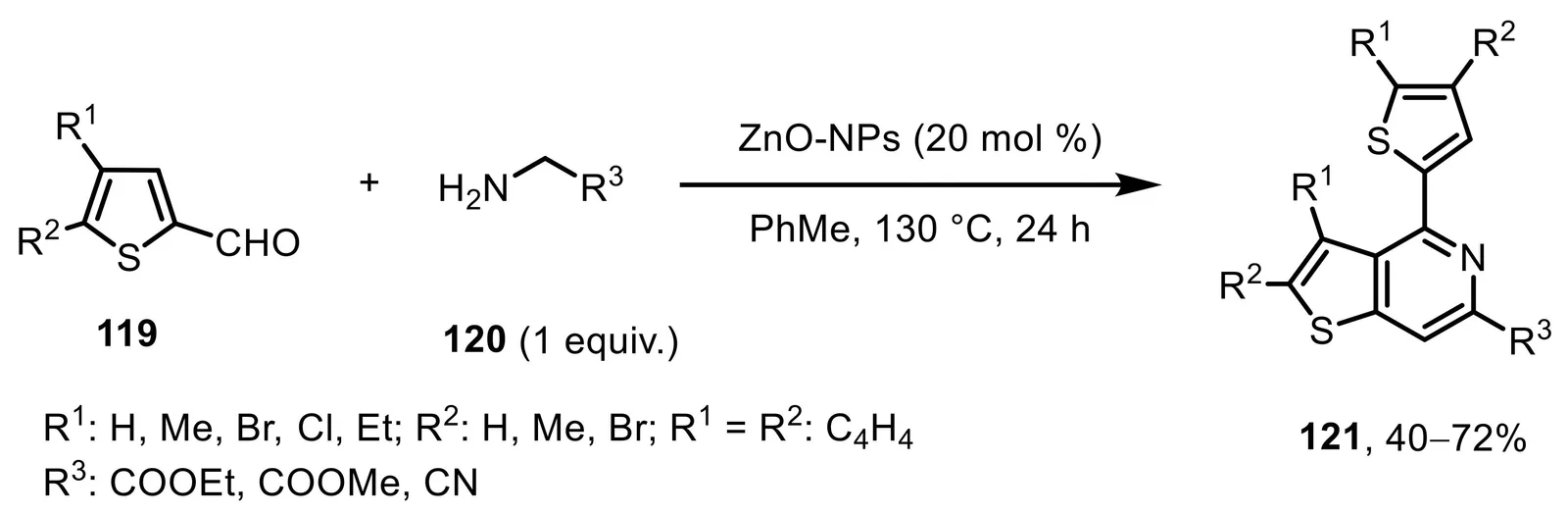
Synthesis of thieno [3,2-*c*]pyridines **121** catalyzed by zinc oxide nanoparticles.

In addition to homogenous catalysts, heterogeneous systems are also utilized. For example, Kumar et al. described a new one-step ZnO nanoparticle-catalyzed (ZnO-NPs) double condensation of substituted thiophene-2-carbaldehydes **119** with glycine esters **120** (Scheme 28) [107]. The reaction proceeds through a mechanism similar to the Hantzsch synthesis, with ZnO-NPs acting as bifunctional Lewis acid–base catalyst. Notably, neither bulk ZnO nor homogeneous zinc salts provided comparable activity, highlighting the crucial role of the nanosized morphology. The substrate scope included various alkyl- and halogen-substituted thiophene-2-carbaldehydes and glycine esters.

### 2.3. Simultaneous Formation of Both Heterocycles

This section covers reactions in which both heterocyclic rings—the pyridine and the thiophene—are formed simultaneously during the reaction, with neither ring being present in the starting materials.

**Scheme 29:**
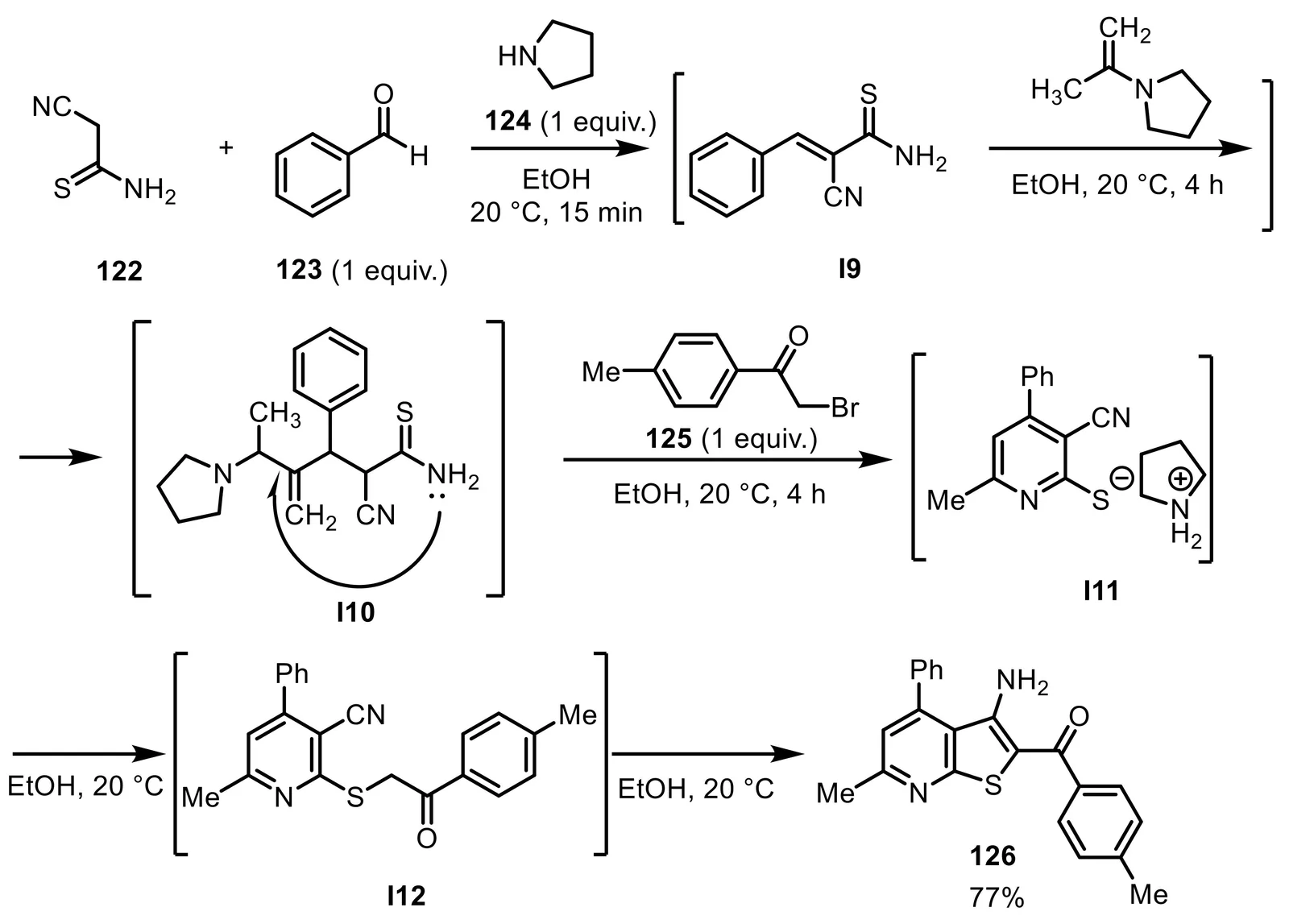
Multi-component synthesis of thieno [2,3-*b*]pyridine **126**.

The group of Dyachenko [108] described the one-pot synthesis of (3-amino-6-methyl-4-phenylthieno [2,3-*b*]pyridin-2-yl)(*p*-tolyl)methanone (**126**) via the multicomponent condensation of cyanothioacetamide (**122**) with benzaldehyde (**123**) and pyrrolidine **124**, followed by S-alkylation with 2-bromo-1-(*p*-tolyl)ethan-1-one (**125**) and Thorpe–Ziegler cyclization. The product **126** was obtained in 77% yield from the starting materials (Scheme 29).

**Scheme 30:**
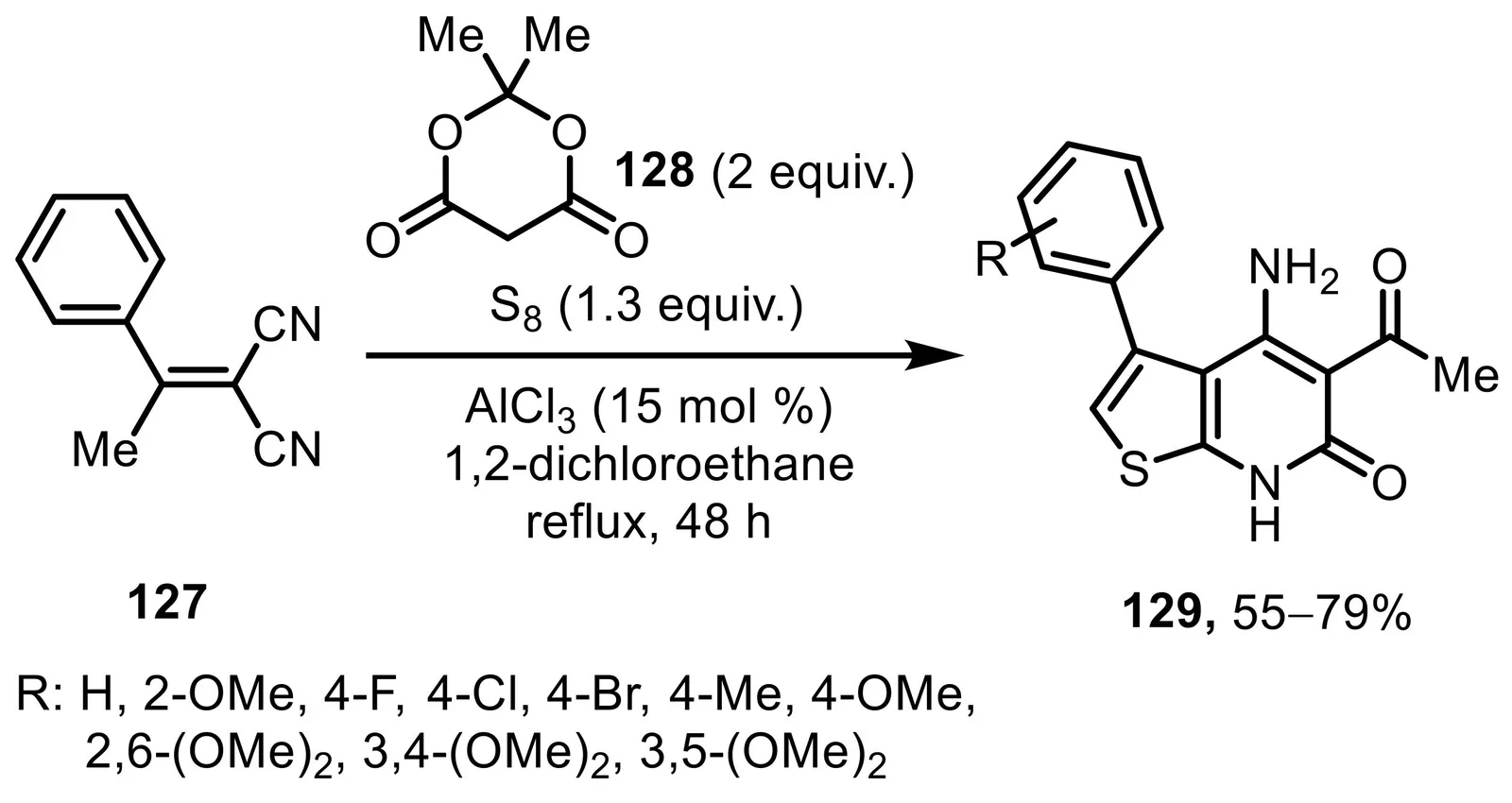
Multicomponent reaction of 2-(1-arylethylidene)malononitriles **127** with elemental sulfur and dimedone **128**.

Mehrabi and colleagues [109] developed a four-component synthesis of 5-acetyl-4-amino-3-arylthieno [2,3-*b*]pyridin-6(7*H*)-ones **129** via a tandem Gewald reaction/pyridine ring annulation, starting from 2-(1-arylethylidene)malononitriles **127**, elemental sulfur, and dimedone (**128**) (Scheme 30). The use of dimedone as a CH-acid enabled the formation of the pyridinone ring through intramolecular cyclization, with electron-withdrawing aryl substituents favoring higher yields (55–79%).

**Scheme 31:**
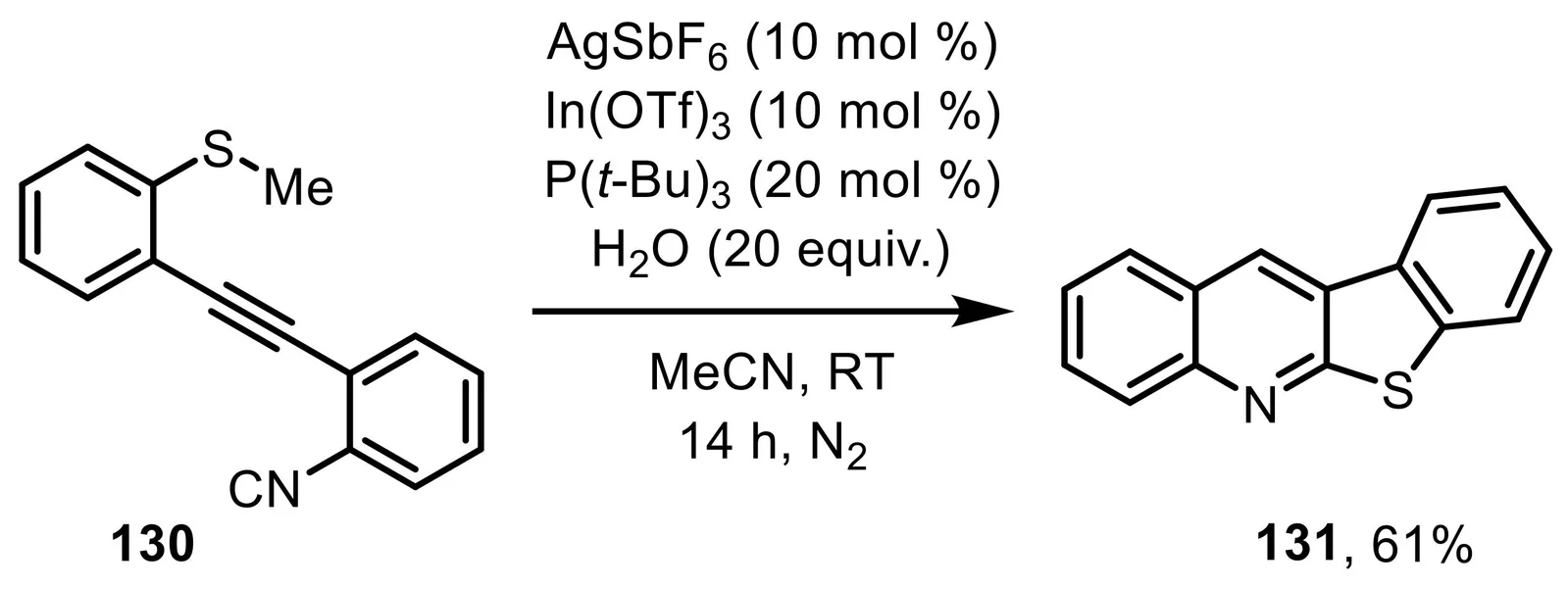
Synthesis of benzo [4,5]thieno [2,3-*b*]quinoline (**131**) via AgSbF_6_/In(OTf)_3_-catalyzed reaction of (2-((2-isocyanophenyl)ethynyl)phenyl)(methyl)sulfane (**130**) with acetonitrile.

The construction of the thienopyrimidinone system through the simultaneous formation of both heterocyclic rings can be achieved not only via multicomponent reactions but also through the heterocyclization of a suitable substrate. For example, Liu and colleagues demonstrated that using (2-((2-isocyanophenyl)ethynyl)phenyl)(methyl)sulfane (**130**) as a substrate for AgSbF_6_/In(OTf)_3_-catalyzed reaction with acetonitrile led to the formation of an intramolecular tetracyclization product, namely, benzo [4,5]thieno [2,3-*b*]quinoline (**131**) (Scheme 31) [110]. In this transformation, both the pyridine and thiophene rings are constructed simultaneously in a single operation.

**Scheme 32:**
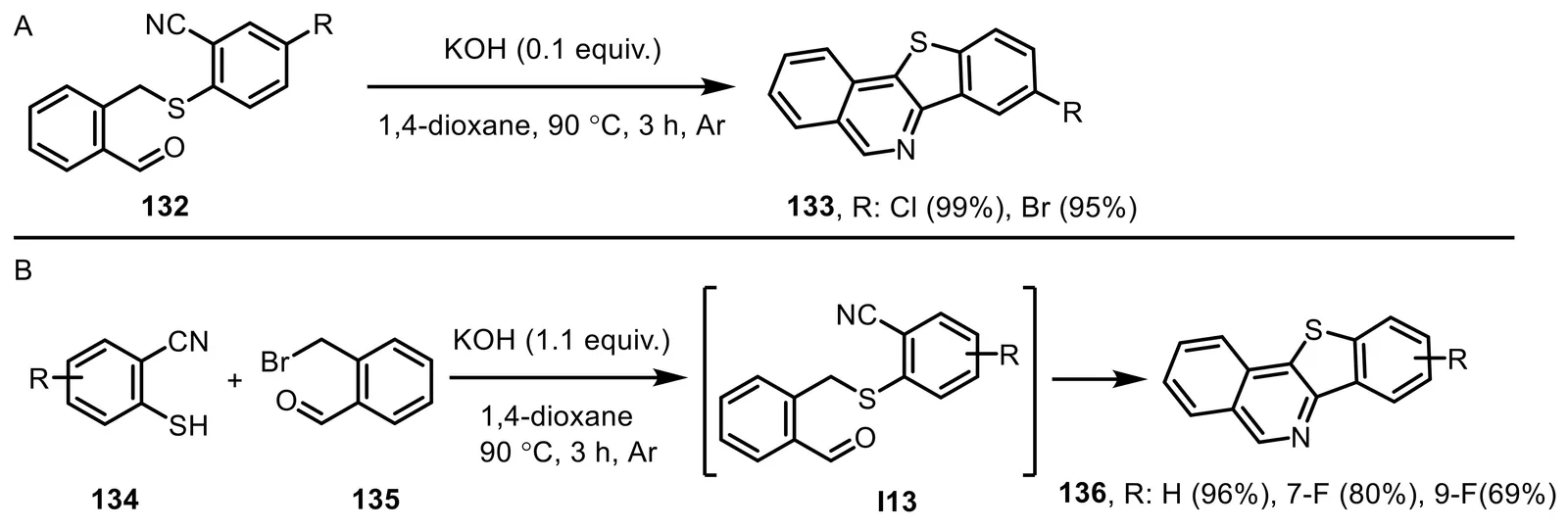
One-pot Synthesis of thienopyridines from nonheterocyclic compounds: (**A**) cyclization of 2-((2-formylbenzyl)thio)benzonitriles and (**B**) condensation of 2-mercaptobenzonitriles and 2-(bromomethyl)benzaldehyde.

Another example of the cyclization with the formation of both heterocycles was provided by Yang and colleagues [111]. They carried out the cyclization of 2-((2-formylbenzyl)thio)benzonitriles **132** into the corresponding benzo [4,5]thieno [3,2-*c*]isoquinolines **133** by treating the starting substrate with KOH in dry 1,4-dioxane under an inert atmosphere (Scheme 32A). Moreover, it was demonstrated that 2-((2-formylbenzyl)thio)benzonitriles **136** can be synthesized in situ from the corresponding 2-mercaptobenzonitriles **134** and 2-(bromomethyl)benzaldehyde (**135**) (Scheme 32B).

### 2.4. Functionalization of the Thienopyridine Scaffold with the Formation of Fused Systems

In many cases, thienopyridines bearing peripheral functional groups, predominantly at the thiophene ring, can undergo annulation reactions to afford tri- and polyheterocyclic systems. These transformations expand the structural diversity of thienopyridine-based compounds and provide access to more complex fused scaffolds with potential biological relevance. Thus, such transformations were listed in this section.

**Scheme 33:**
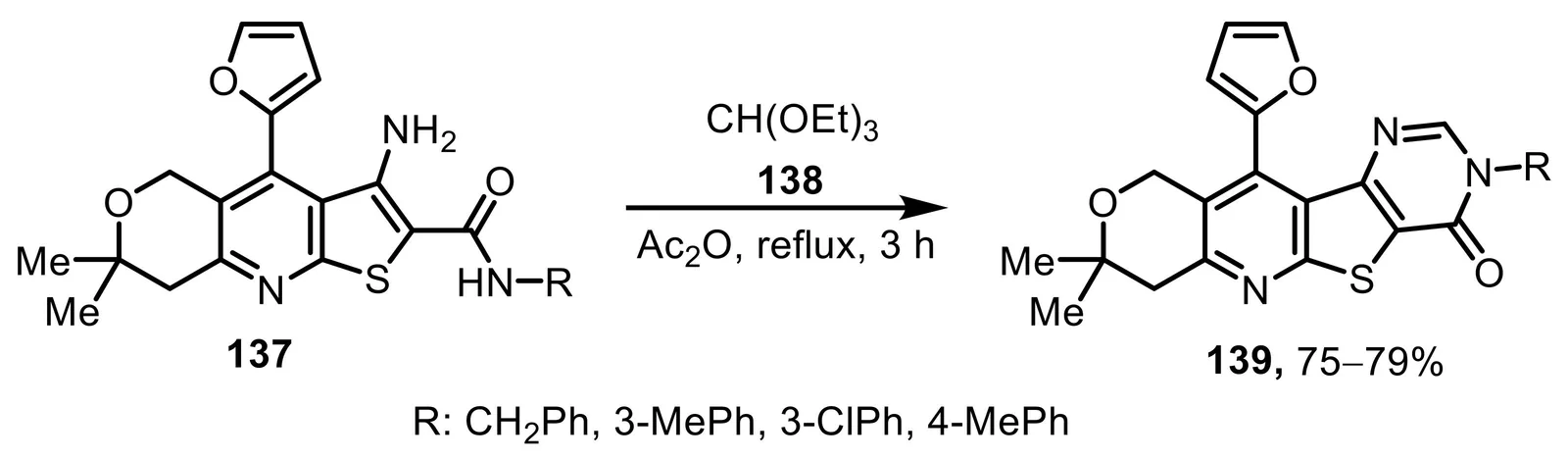
Condensation of 3-aminothieno [2,3-*b*]pyridine-2-carboxamides **137** with triethyl orthoformate (**138**).

One of the most common transformations in this area is the reaction of 3-aminothieno [2,3-*b*]pyridine-2-carboxamides with carbonyl compounds, leading to pyrido [3′,2′:4,5]thieno [3,2-*d*]pyrimidin-4(3*H*)-one and related fused systems. An example of such a reaction was reported by Dabayeva and colleagues [112]. The condensation of the amino and carboxamide groups of thienopyridines **137** with triethyl orthoformate (**138**) furnished the corresponding pyrido [3′,2′:4,5]thieno [3,2-*d*]pyrimidin-4(3*H*)-ones **139** (Scheme 33). The same reaction was also described in the study of Mohamed et al. [113].

**Scheme 34:**
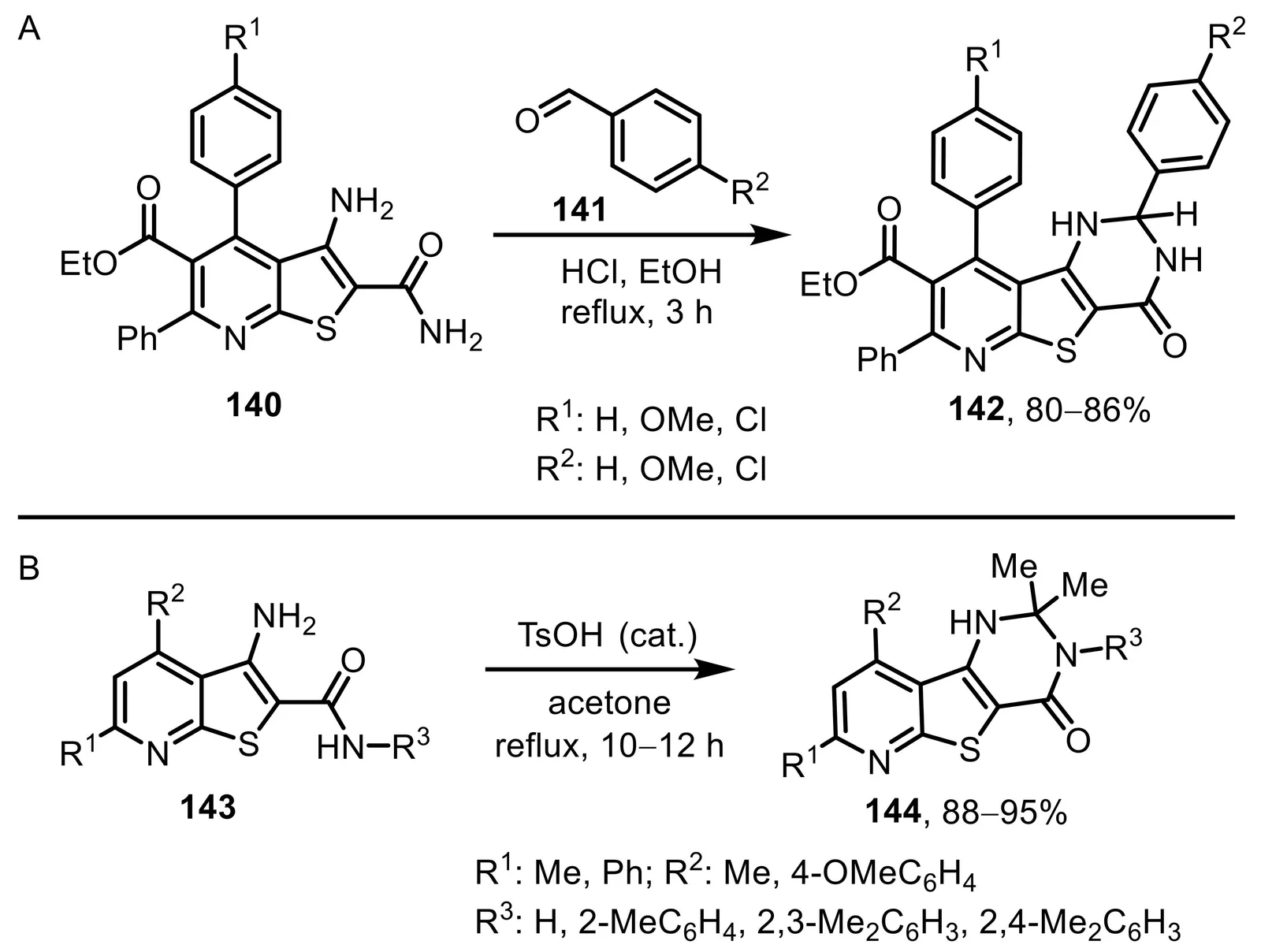
Condensation of 3-aminothieno [2,3-*b*]pyridine-2-carboxamides with carbonyl compounds: (**A**) benzaldehydes and (**B**) acetone.

Al-Waleedy and colleagues [114] synthesized a series of pyrido [3′,2′:4,5]thieno [3,2-*d*]pyrimidinone derivatives **142** via reaction of 3-amino-2-carbamoyl-6-phenylthieno [2,3-*b*]pyridine-5-carboxylates **140** with various aromatic aldehydes **141** under acidic conditions (Scheme 34A). A similar process, however, involving acetone as the carbonyl component, was reported by Dotsenko and colleagues (Scheme 34B) [115].

**Scheme 35:**
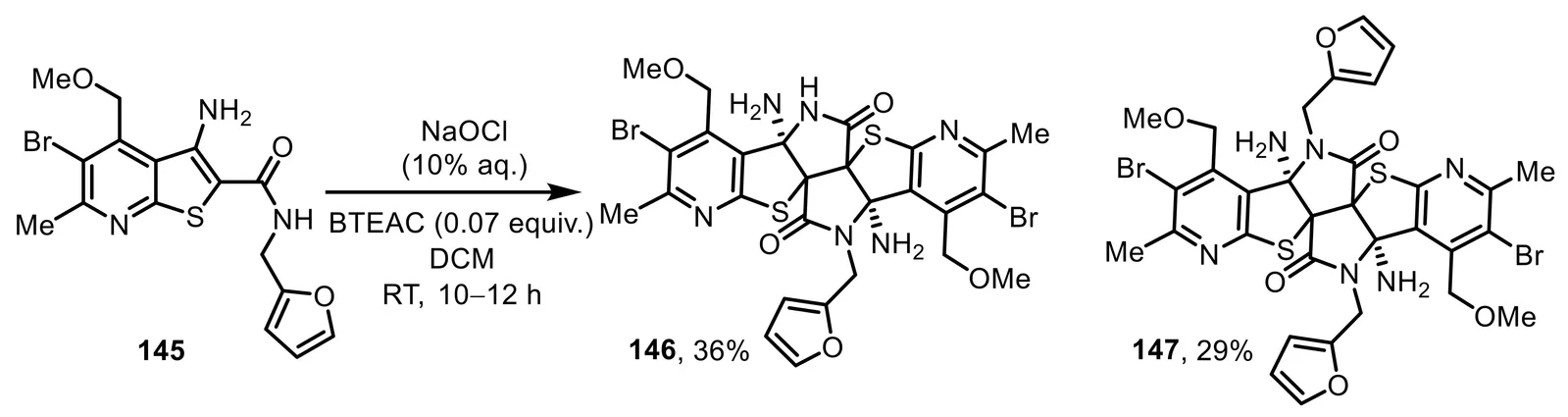
Dimerization of 3-aminothieno [2,3-*b*]pyridine-2-carboxamide **145** in presence of sodium hypochlorite.

Stroganova and colleagues [116] reported an unusual oxidative dimerization of 3-aminothieno [2,3-*b*]pyridine-2-carboxamide **145** upon treatment with sodium hypochlorite, giving *N’,N’*-diaryl-substituted bislactams **146**. The reaction proceeds via electrophilic chlorination of the thiophene ring followed by nucleophilic attack of a second molecule and subsequent intramolecular cyclization (**147**) (Scheme 35).

**Scheme 36:**
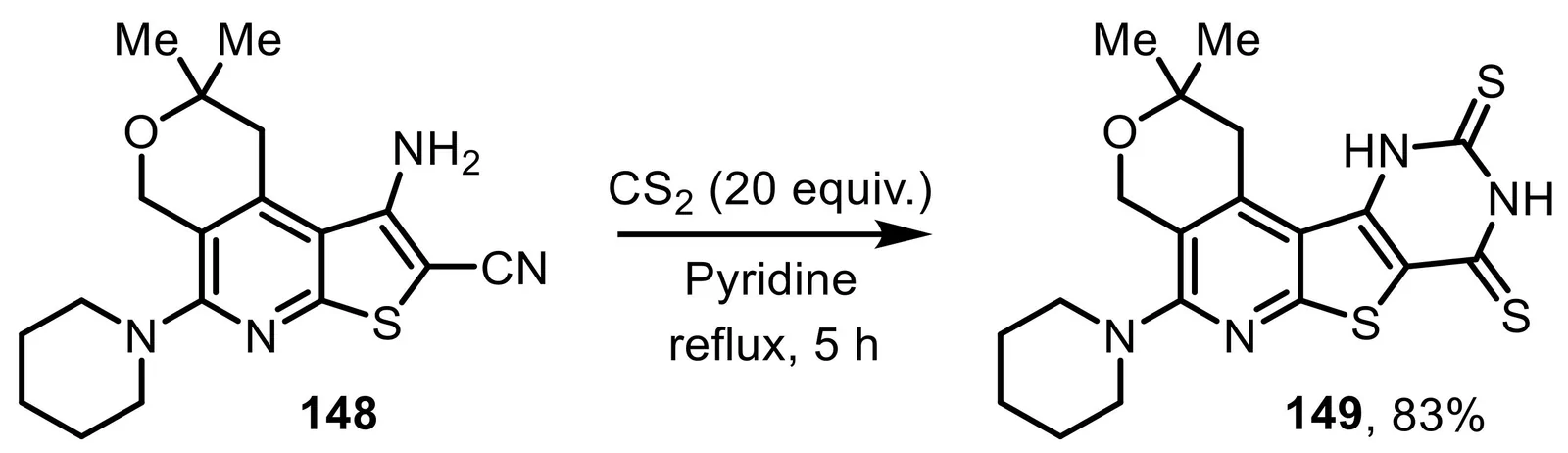
Reaction of 3-amino-thieno [2,3-b]pyridine-2-carbonitrile **148** with carbon disulfide.

The annulation of 3-aminothieno [2,3-*b*]pyridines with carbonyl electrophiles is not restricted to carboxamide derivatives; other functional groups, such as cyano, carboxy, and alkoxycarbonyl functionalities at the 2-position, also enable this transformation. For example, Dashyan et al. [55] reported that treatment of aminonitrile **148** with carbon disulfide in pyridine at reflux afforded tetracyclic pyrimidinedithione **149** in 83% yield instead of the expected thiazine derivative (Scheme 36). The reaction proceeds via the initial formation of a six-membered thiazine ring followed by its rearrangement into the thermodynamically more favorable pyrimidine ring.

**Scheme 37:**
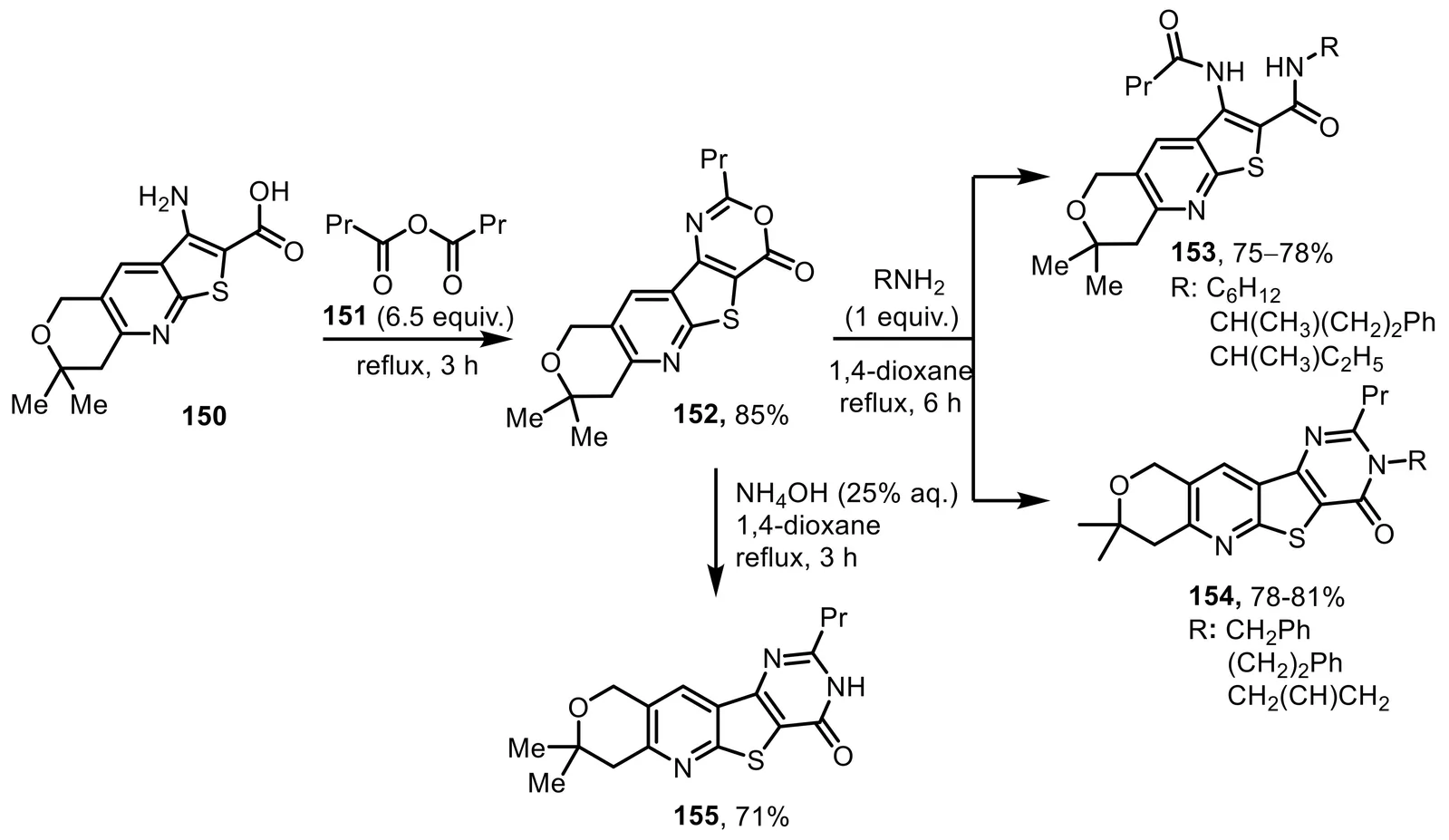
Synthesis of thieno [3,2-*d*][1,3]oxazin-4-one **152** and its reactions with N-nucleophiles.

Dabaeva and colleagues [117] reported the annulation of a 1,3-oxazine ring onto a preformed thienopyridine scaffold by reacting vicinal amino-carboxyl acid **150** with butyric anhydride **151** under reflux, providing oxazinone **152** in 85% yield (Scheme 37). This compound can be further treated with various N-nucleophiles, giving acyclic carboxamides **153** or tetracyclic pyrimidinones **154** or **155** depending on the steric hindrance of the amine.

**Scheme 38:**
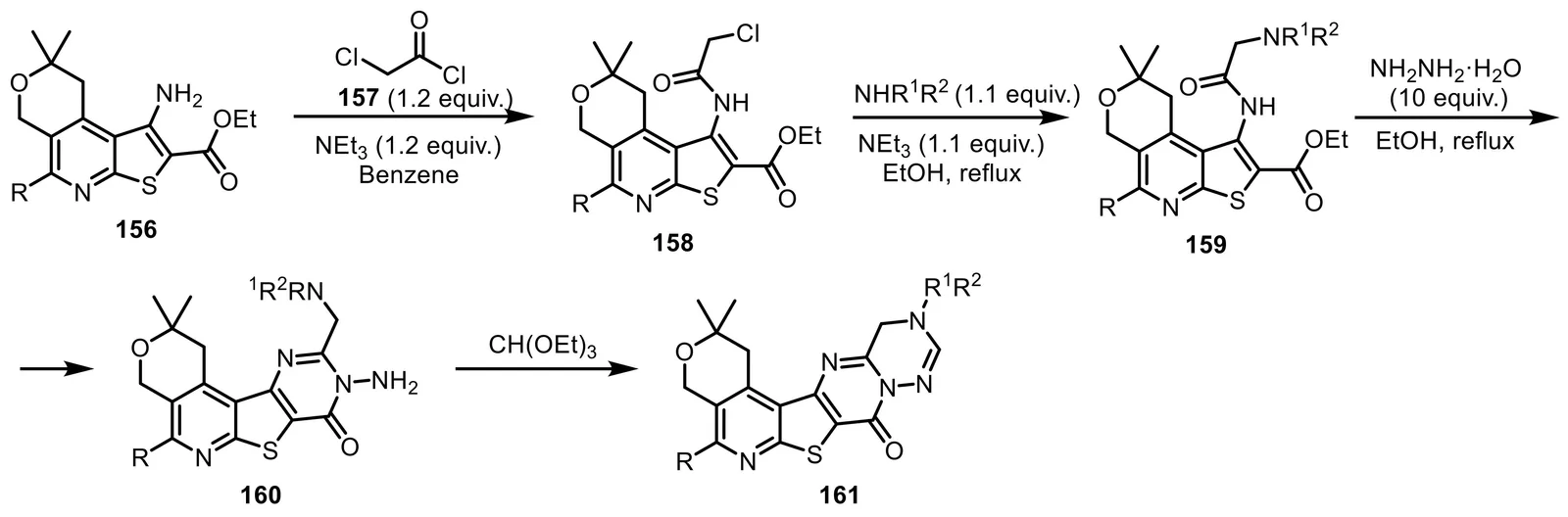
Universal method for completing the thieno [2,3-*b*]pyridine scaffold with pyrimidine and 1,2,4-triazine rings to obtain pentacyclic pyrimido [2,1-*f*][1,2,4]triazines **161**.

Sirakanyan et al. [118] developed an approach for the sequential annulation of pyrimidine and 1,2,4-triazine rings onto a preformed thieno [2,3-*b*]pyridine scaffold (Scheme 38). The sequence begins with acylation of vicinal amino-esters **156** with chloroacetyl chloride (**157**), followed by substitution of the chlorine atom with secondary amines to give compounds **158**. Their treatment with hydrazine hydrate triggers cyclization involving both the amide and ester groups, affording tetracyclic pyrimidinones **160**. Subsequent condensation of the free NH group in **160** with triethyl orthoformate closes the 1,2,4-triazine ring, forming pentacyclic pyrimido [2,1-*f*][1,2,4]triazines **161** in 75–84% yield.

**Scheme 39:**
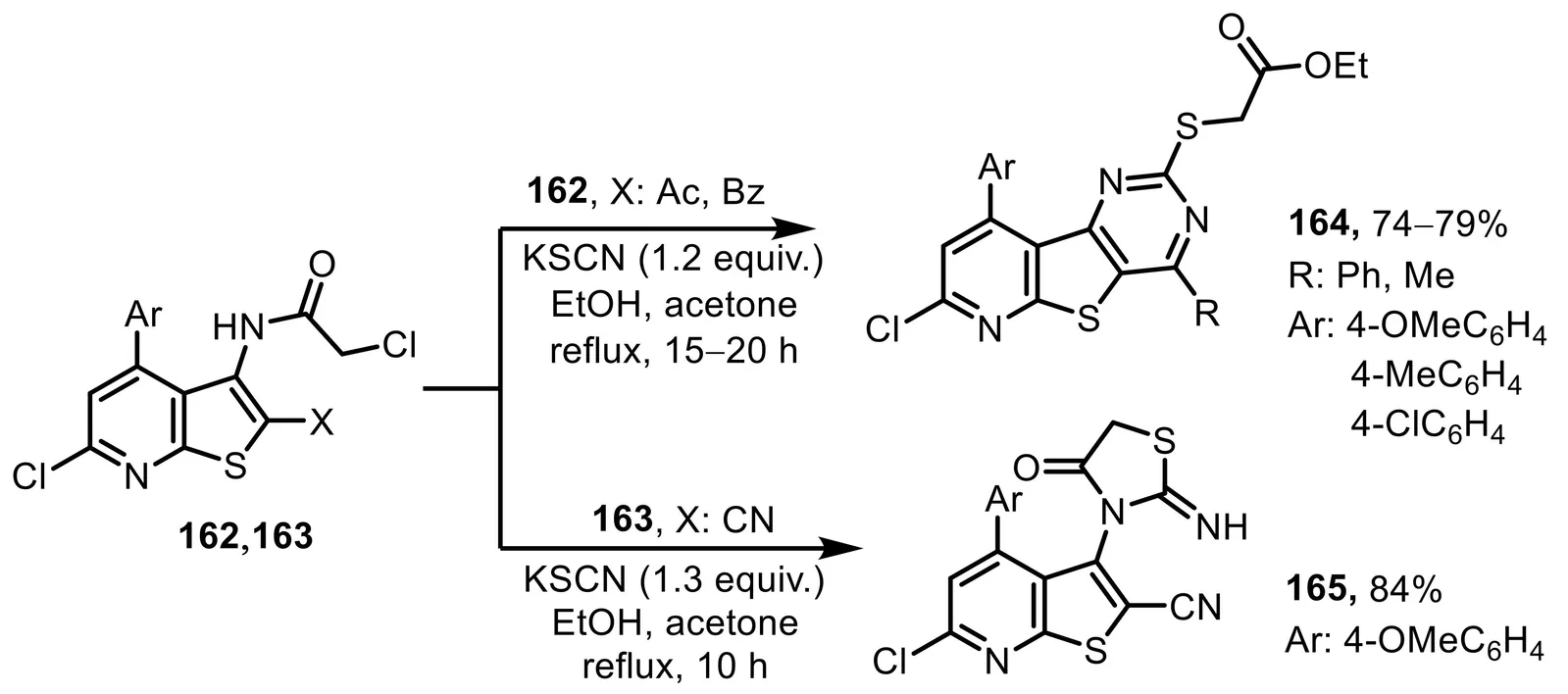
Synthesis of pyrido [3′,2′:4,5]thieno [3,2-*d*]pyrimidines **164a** and **165**.

In the work of Rudenko et al. [119], 3-amino-4-aryl-6-phenylthieno [2,3-*b*]pyridines **162** and **163** bearing a free amino group at position 3 served as starting compounds for the synthesis of pyrido [3′,2′:4,5]thieno [3,2-*d*]pyrimidines **164** via an annulation with chloro-acetyl chloride and KSCN (Scheme 39). Notably, the annulation occurs only for thienopyridines bearing an acyl substituent at C2 of the thiophene ring, whereas reaction of 2-cyano derivative with KSCN gave a thiazole moiety **165**.

**Scheme 40:**
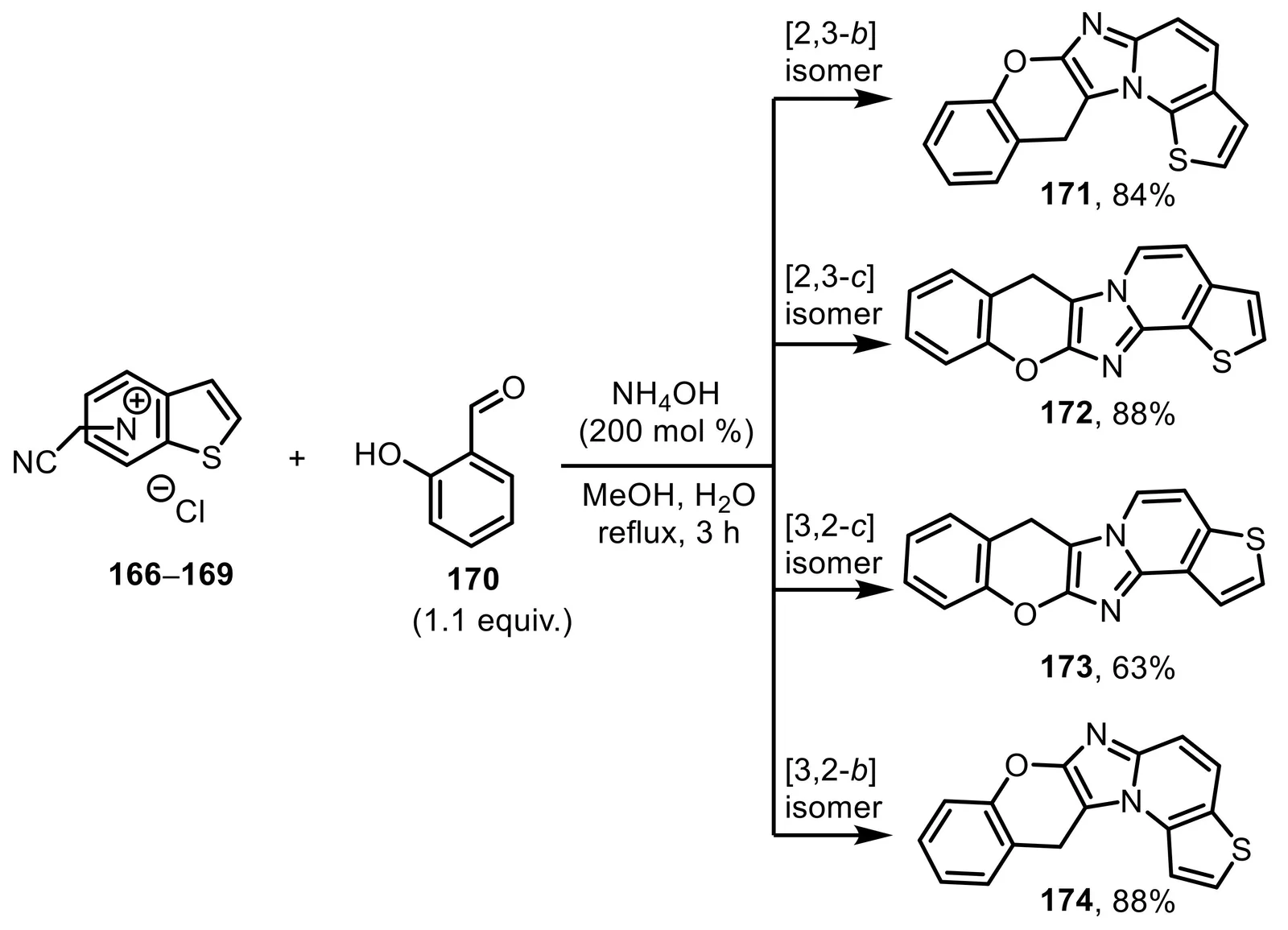
Synthesis of chromeno-imidazo-thienopyridines **171–174** from *N*-(cyanomethyl)pyridinium salts of all four isomeric thienopyridines and *o*-hydroxybenzaldehydes **166–169**.

Storozhenko and colleagues [120] described the metal-free domino annulation of imidazole and chromene rings onto the pyridine nitrogen of any of the four isomeric thienopyridines. The sequence involves conversion of thienopyridines to *N*-(cyanomethyl)pyridinium chlorides **166**–**169** (77–90%), followed by cascade reaction with salicylaldehyde **170** and ammonium acetate, which proceeds via Knoevenagel condensation, intramolecular nucleophilic cyclization, and 1,3-hydride shift to afford pentacyclic chromeno [2′,3′:4,5]imidazo [1,2-*a*]thienopyridines **171**–**174** in moderate to good yields (Scheme 40).

**Scheme 41:**
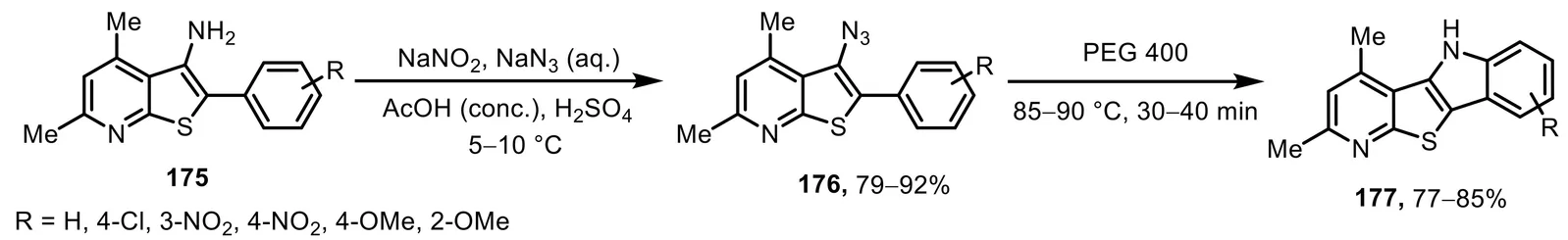
Annulation of the indole ring onto the thieno [2,3-*b*]pyridine scaffold.

Stroganova and colleagues [51] reported the annulation of an indole ring onto the thieno [2,3-*b*]pyridine scaffold via intermediate nitrene formation. Diazotization of 3-aminothienopyridines **175** followed by treatment with sodium azide afforded 3-azidothieno [2,3-*b*]pyridines **176**. Heating these azides in PEG-400 at 85–90 °C induced nitrogen elimination with generation of a nitrene intermediate, which underwent intramolecular C–H insertion into the neighboring aryl substituent at position 2, furnishing tetracyclic pyrido [3′,2′:4,5]thieno [3,2-*b*]indoles **177** in 77–89% yields (Scheme 41). The ratio of regioisomeric indoles from nitro-substituted precursor was found to be solvent-dependent (1,4-dioxane vs. PEG-400), attributed to the influence of the medium on the regioselectivity of C–H insertion.

**Scheme 42:**
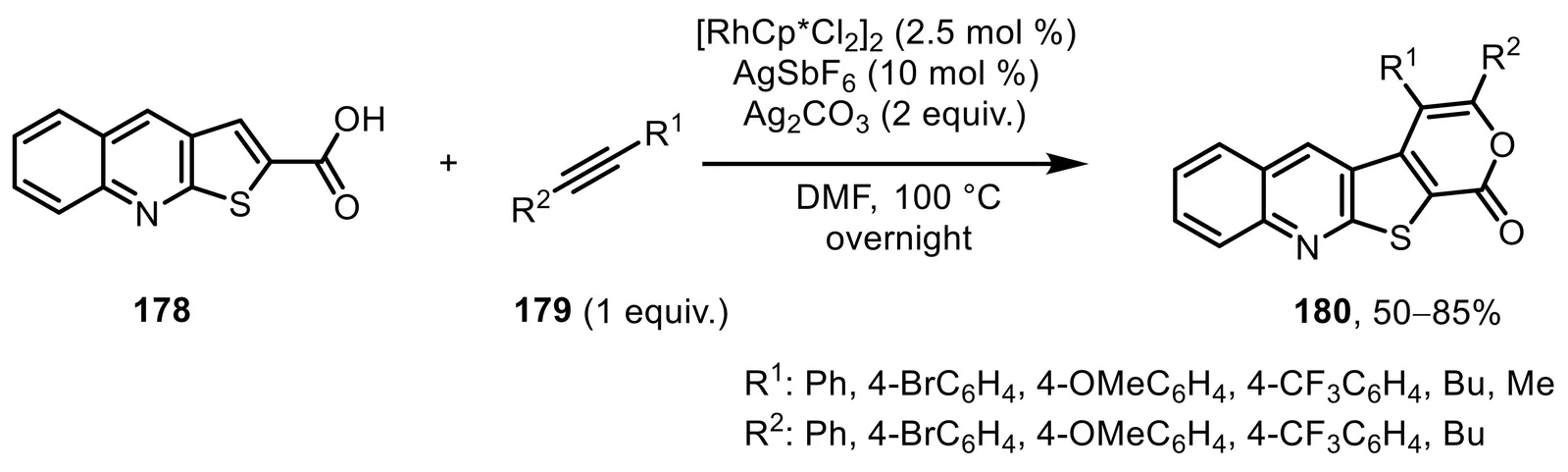
Rh(III)-catalyzed reaction of thieno [2,3-*b*]quinoline-2-carboxylic acid **178** with alkynes **179**.

Martins and colleagues [121] synthesized tetracyclic lactones fused to thieno [2,3-*b*]quinoline **180** via Rh(III)-catalyzed C–H activation with alkynes **179** in DMF at 100 °C, wherein the carboxyl group acts as a directing group (Scheme 42).

## 3. Medicinal Application of Thienopyridines

### 3.1. Utility of Thienopyridines in Medication of Infectious Disease

Compounds containing the thienopyridine scaffold are widely investigated for the treatment of infectious disease [122]. In the reviewed period this trend has remained.

**Figure 3:**
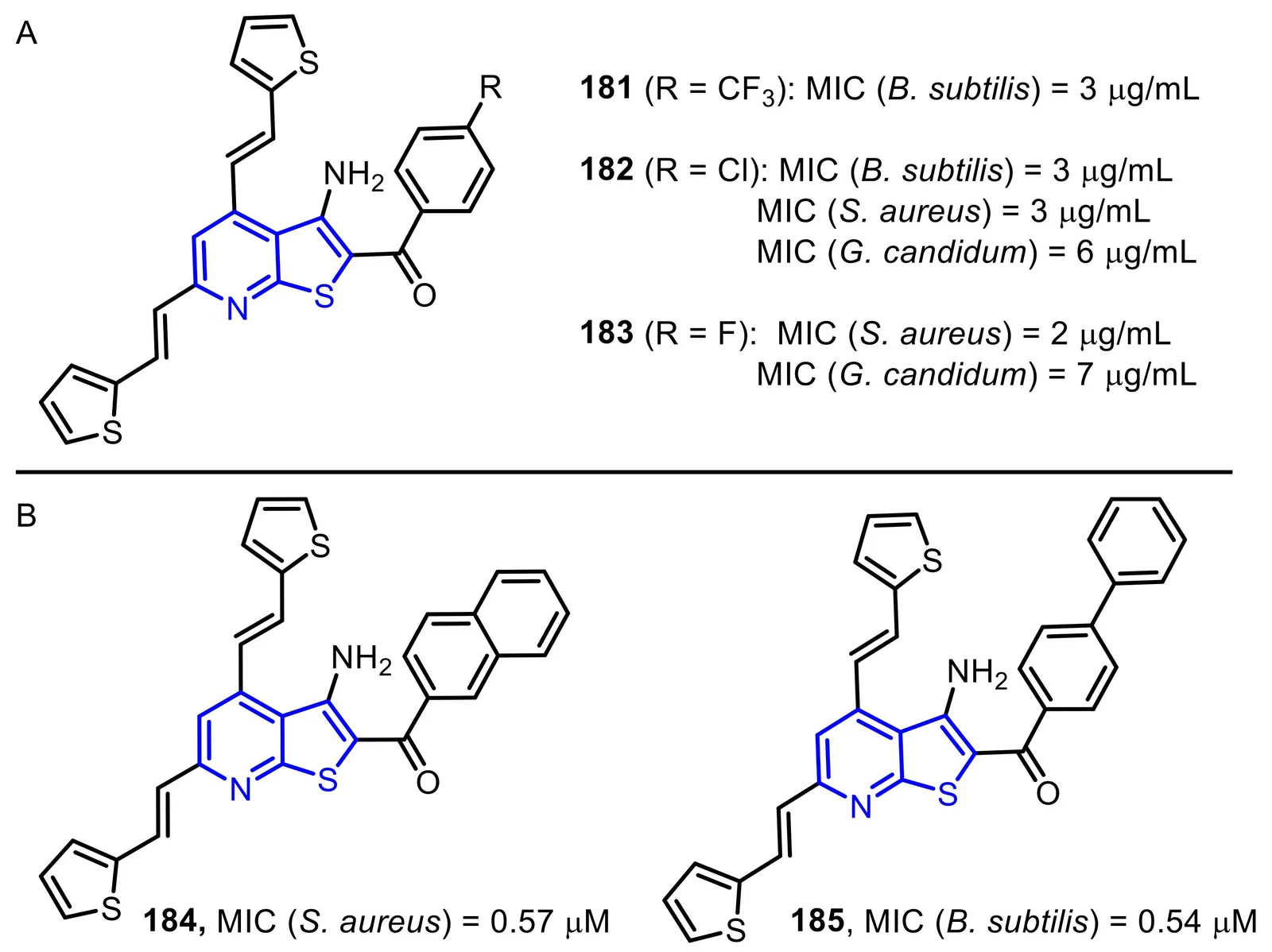
Antimicrobial and antifungal activity of thieno [2,3-*b*]pyridine-thiophene hybrids: (**A**) structures and MIC values of compounds **181**–**183**; (**B**) structures and MIC values of compounds **184** and **185**.

Sayed and colleagues performed two studies of antimicrobial and antifungal activity of thieno [2,3-*b*]pyridine-thiophene hybrids [123,124]. In the first work [123], among all synthesized thienopyridines, compounds **182** and **183** were most potent in antimicrobial experiments (Figure 3A). Their activity against *S. aureus* of was comparable to the control drug ciprofloxacin (minimum inhibitory concentrations (MICs) values are 3 µg/mL and 2 µg/mL, respectively). Their structures are distinguished by the presence of a halophenylketone at the 2-position of thieno [2,3-*b*]pyridine ring. Compound **181** showed potent activity against another Gram-positive strain—*B. subtilis*—with an MIC of 3 µg/mL. At the same time, compound **182** also showed significant activity against *B. subtilis*, indicating its broad-spectrum action against Gram-positive bacteria. The antifungal study revealed that compounds **182** and **183** inhibit *G. candidum*, with MIC values ranging from 6 to 7 µg/mL. In addition, compound **182** showed the highest activity among the series against other fungi. Molecular docking of the binding of hit compounds to phosphotransferase-IIa (APH(2”)-IIa)—a common bacterial resistance factor [125]—was performed. Compound **183** showed high binding affinity to APH(2”)-IIa through the formation of hydrogen bonds with Asp213 and Tyr272. For compound **182**, a study of binding with aspartate proteinases (Saps) was performed. π-Alkyl hydrophobic noncovalent interactions with residues Ile12, Arg120, and Trp51 were detected. Structure–activity analysis (SAR) allowed the authors to conclude that the presence of halogens in the structure of phenylketones at the 2-position of the thieno [2,3-*b*]pyridine ring is of high importance, providing binding to the target enzyme and high activity indicators.

In the second work [124], thienopyridines bearing bulky aromatic groups (biphenyl and naphthalene) were investigated (Figure 3B). Compound **184** demonstrated high activity against *S. aureus* with a minimum inhibitory concentration (MIC) of 0.57 μM, comparable to the control ciprofloxacin (MIC 1.2 μM). Compound **185** demonstrated activity against *B. subtilis* with an MIC of 0.54 μM. Molecular docking revealed strong binding of compounds **184** and **185** with the DNA gyrase enzyme, which is an important enzyme involved in bacterial DNA replication [126]. It was shown that compounds **184** and **185** form favorable π-alkyl and π-sulfur noncovalent interactions with amino acid residues Phe95 and Val109.

**Figure 4:**
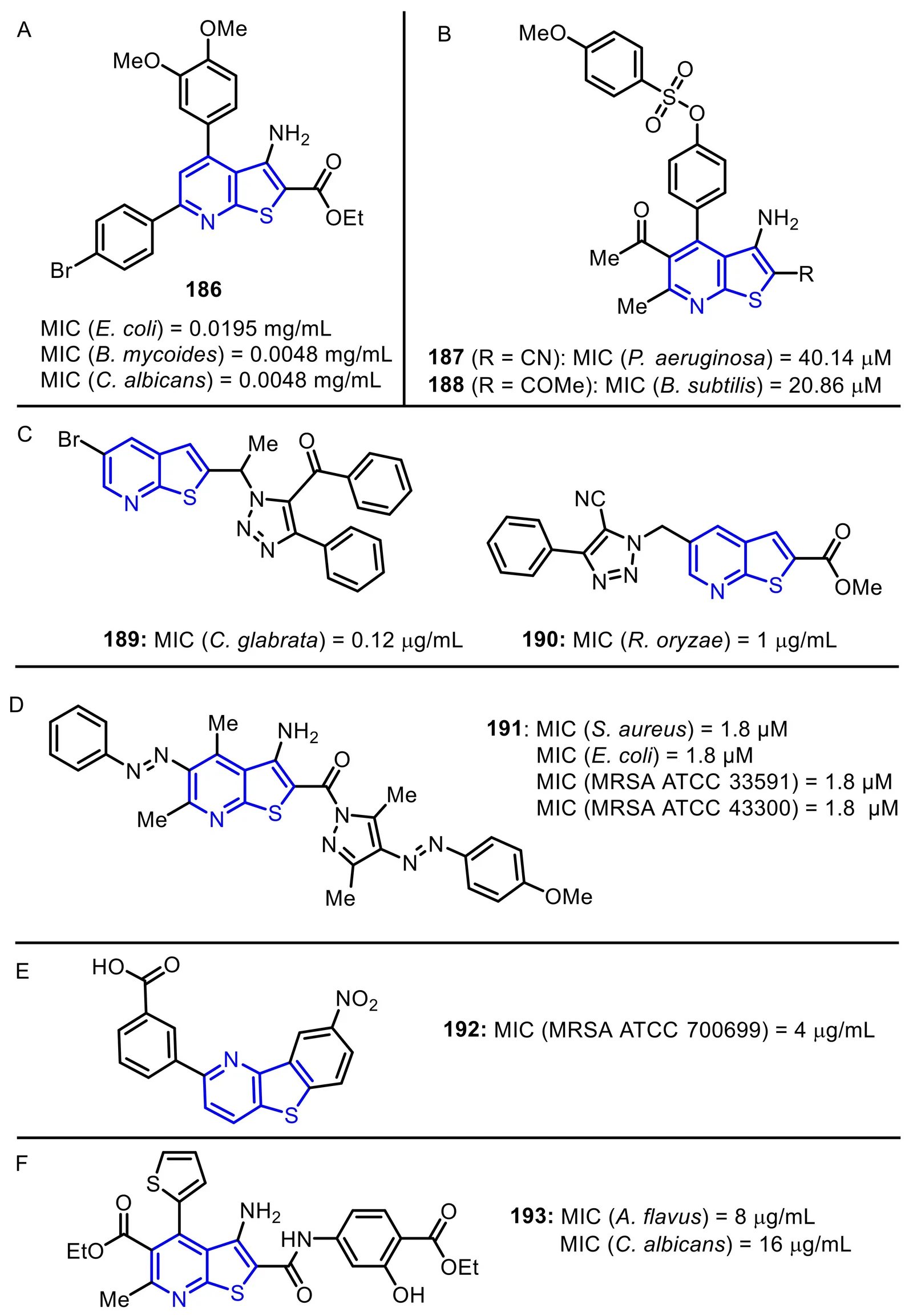
Diverse thienopyridines demonstrating antibacterial and antifungal activity: (**A**) structure and MIC values of compound **186**; (**B**) structures and MIC values of compounds **187** and **188**; (**C**) structures and MIC values of compounds **189** and **190**; (**D**) structure and MIC values of compound **191**; (**E**) structure and MIC value of compound **192**; (**F**) structure and MIC values of compound **193**.

Elsayed and colleagues designed a new series of diaryl-substituted 3-aminothienopyridines as antimicrobial agents (Figure 4A) [127]. Antibacterial activity studies were performed on strains of *E. coli*, *B. mycoides*, and *C. albicans*. Compound **186** inhibited the growth of *B. mycoides* and *E. coli* at an MIC of 0.0048 mg/mL and 0.0195 mg/mL, respectively. Moreover, most of the synthesized compounds showed activity against *C. albicans* that exceeded the activity values of the control compounds (ampicillin, gentamicin, and tobramycin). Structure-activity analysis showed strong antimicrobial activity of thienopyridines, with the highest inhibition activity than that observed in the pyridine derivatives.

Altwaijry and colleagues [128] synthesized, via a one-pot procedure, number of 4-arylthieno [2,3-*b*]pyridin-3-amines and evaluated their antimicrobial activity (Figure 4B). Among the studied thienopyridines, compound **187** displayed moderate activity against *P. aeruginosa* (MIC = 40.14 µM), while **188** showed selective activity against *B. subtilis* (MIC = 20.86 µM), outperforming tetracycline (62.40 µM) against this strain. However, compounds containing 2--thioxopyridine and pyridopyrimidine demonstrated higher activity against the studied microorganisms.

Antifungal agents based on thienopyridines with a 1,2,3-triazole moiety at the C2 (series 1) or C5 (series 2) positions were developed by Ramírez-Villalva and colleagues (Figure 4C) [129]. Among the studied molecules, compound **189** with phenyl and benzoyl groups in the triazole ring showed the best activity against the yeast fungus *C. glabrata*, with an inhibitory concentration of MIC = 0.12 μg/mL, which is lower than that of the reference compound itraconazole at 1 μg/mL. Compound **190** with phenyl and cyano substituents in the triazole ring demonstrated activity comparable to the control against strain of the filamentous mycelial fungi *R. oryzae*—with an MIC of 1 μg/mL.

Ahmed and colleagues [130] synthesized a series of thieno [2,3-*b*]pyridine-based 1*H*-pyrazoles bearing arylazo units as antibacterial agents targeting drug-resistant pathogens, including methicillin-resistant *S. aureus* (MRSA) (Figure 4D). Among the synthesized compounds, derivative **191**, containing phenyldiazenyl and (4-methoxyphenyl)diazenyl substituents at the thienopyridine-C5 and pyrazole-C4 positions, respectively, exhibited the highest antibacterial activity, surpassing ciprofloxacin against *S. aureus* and *E. coli* (MIC = 1.8/1.8 µM). Moreover, **191** also outperformed linezolid against MRSA (MIC = 1.8 µM), proving its potential for combating drug-resistant bacterial infections. SAR analysis revealed that simultaneous incorporation of arylazo substituents at the thienopyridine C5 and pyrazole C4 positions, particularly a combination of phenyldiazenyl and (4-methoxyphenyl)diazenyl groups, gave the highest antibacterial potency, indicating that both the number and electronic nature of the appended aromatic azo groups substantially affect activity against MRSA.

Roy and colleagues [131] developed a domino protocol for the synthesis of 3-amino--2-formylbenzothiophenes from 2--fluorobenzonitriles and 2,5-dihydroxy-1,4-dithiane. These amino aldehydes were then converted into benzothieno [3,2-*b*]pyridine derivatives via Friedländer reaction with various ketones. The screening of antibacterial activity of synthesized compounds revealed benzothieno [3,2-b]pyridine **192** (Figure 4E) as a potent agent against MRSA.

Bakhite and colleagues [132] prepared a series of 6,7-dihydrothieno [2,3-*c*]isoquinolines and assessed their activity against bacterial and fungal pathogens. Although most compounds exhibited only weak to moderate antibacterial effects, several derivatives showed potent antifungal activity, with MIC values close to the reference drug. Sulfanylpyridine derivatives and pyridinium chloride showed the highest activity among the compounds in the series. At the same time, the imidazoline derivative **193** demonstrated pronounced biological activity (MIC = 8 and 16 µg/mL against *A. flavus* and *C. albicans*, respectively) (Figure 4F). A similar trend was observed by Qahtan and colleagues [133], who synthesized a series of thienopyridine-substituted methyl 4-aminosalicylates. When evaluated against the same panel of bacterial and fungal strains, these compounds likewise showed negligible antibacterial activity but exhibited pronounced antifungal effects. The thieno [3,2-*b*]pyridine fused with a pyrano- moiety and its precursors were evaluated for antimicrobial activity against Gram-positive (*S. aureus, S. pneumoniae*), Gram-negative (*S. typhimurium, E. coli*) bacteria, as well as fungi *C. albicans* and *A. fumigatus* by Althagafi and Abdel-Rhman [134]; the obtained activity values were weak.

A serious challenge currently facing medicinal chemistry is the development of effective anti-tuberculosis therapy [135]. In recent years, important efforts in this field have been associated with the thienopyridine scaffold.

**Figure 5:**
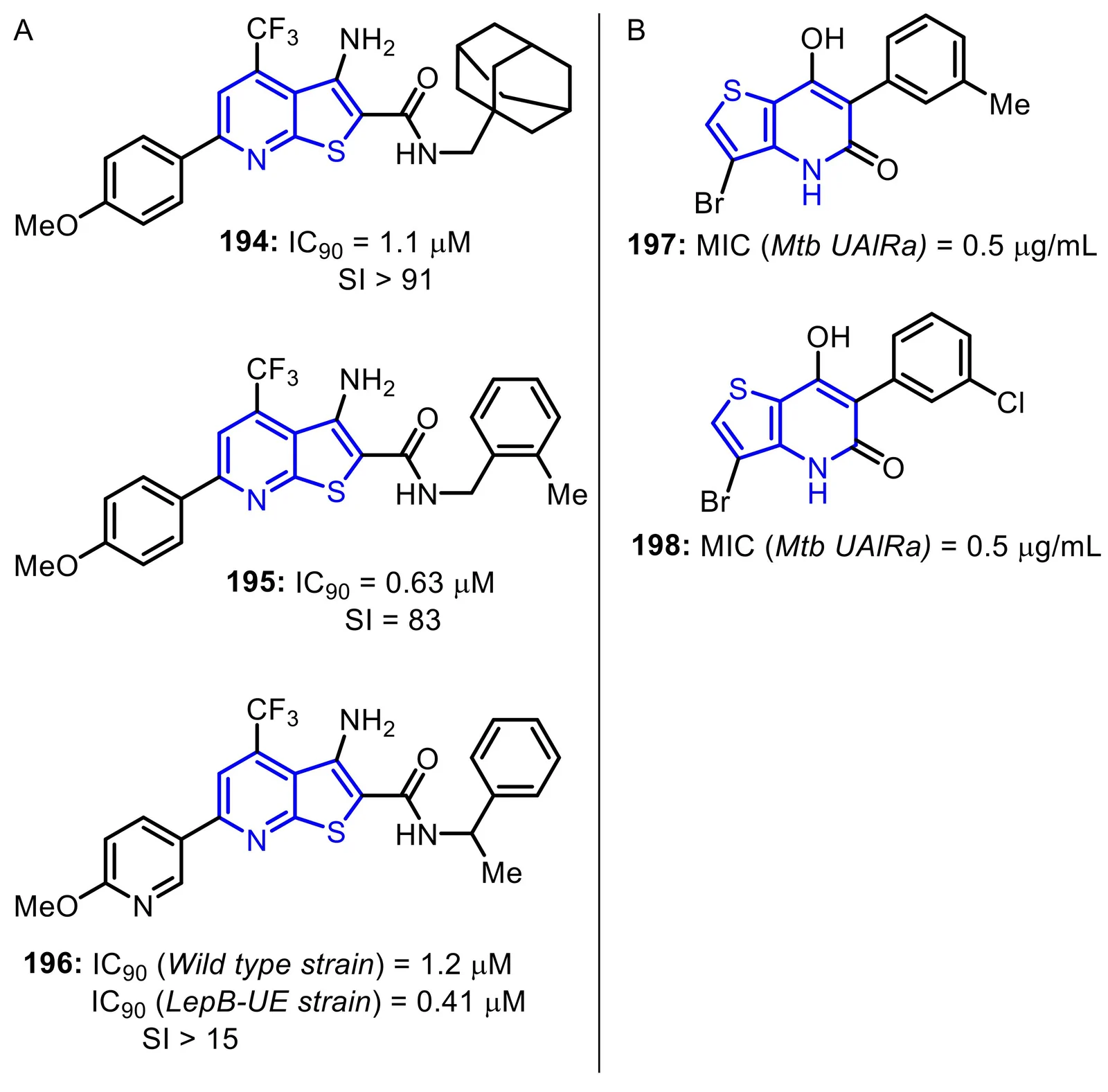
Anti-tubercular agents based on thienopyridine scaffold: (**A**) structures and IC_50_ values of compounds **194**–**196**; (**B**) structures and IC_50_ values of compounds **197** and **198**.

Parish and colleagues [136] investigated a series of 3-aminothieno [2,3-*b*]pyridine-2-carboxamides as inhibitors of type I signal peptidase (LepB), a promising target for anti-tuberculosis drug development [137]. Initial hits active against a LepB-hypomorphic *M. tuberculosis* strain were inactive against the wild-type strain, prompting structural optimization. SAR analysis revealed that simultaneous substitution at both the 4- and 6-positions of the thienopyridine core is essential for activity against the wild-type strain, with the trifluoromethyl group at C4 and aromatic substituents bearing hydrogen bond acceptors at C6 being most favorable. Further optimization of the amide fragment identified secondary amides with bulky groups, particularly 1-adamantylamine (**194**, IC_90_ = 1.2 µM, SI > 91) and 1-phenylethylamine (**195**, IC_90_ = 0.63 µM, SI = 83), as the most promising. The optimized compound **196** combined potent activity against both wild-type (IC_90_ = 1.2 µM) and LepB-hypomorphic (IC_90_ = 0.41 µM) strains, confirming target engagement (Figure 5A). However, the lack of correlation between activity and LepB expression for some analogues suggested the possible involvement of additional, unidentified targets.

Liang and colleagues [138] employed a scaffold-hopping strategy to develop thieno [3,2-*b*]pyridin-5(4*H*)-one derivatives as direct inhibitors of InhA, a NADH-dependent enoyl-acyl carrier protein reductase essential for mycolic acid biosynthesis in *Mycobacterium tuberculosis* [139]. SAR analysis revealed that *ortho-* and *meta*-substitution of the 6-aryl ring (MIC = 0.5–1 µg/mL) is strongly favored, while *para*-substitution abolishes activity; the bromine atom at C3 and the phenolic hydroxyl and lactam groups were identified as critical for potency (Figure 5B). The most promising compounds, **197** (3-MeC_6_H_4_) and **198** (3-ClC_6_H_4_), exhibited potent antimycobacterial activity with no cytotoxicity against normal hepatocytes and no antibacterial or antifungal cross-activity, indicating high target specificity. Compound **197** demonstrated favorable oral pharmacokinetics in rats (T_1_/_2_ = 47.99 h, F = 16.4%) and dose-dependent in vivo efficacy in an acute TB mouse model, including synergistic effects when combined with rifampicin.

**Figure 6:**
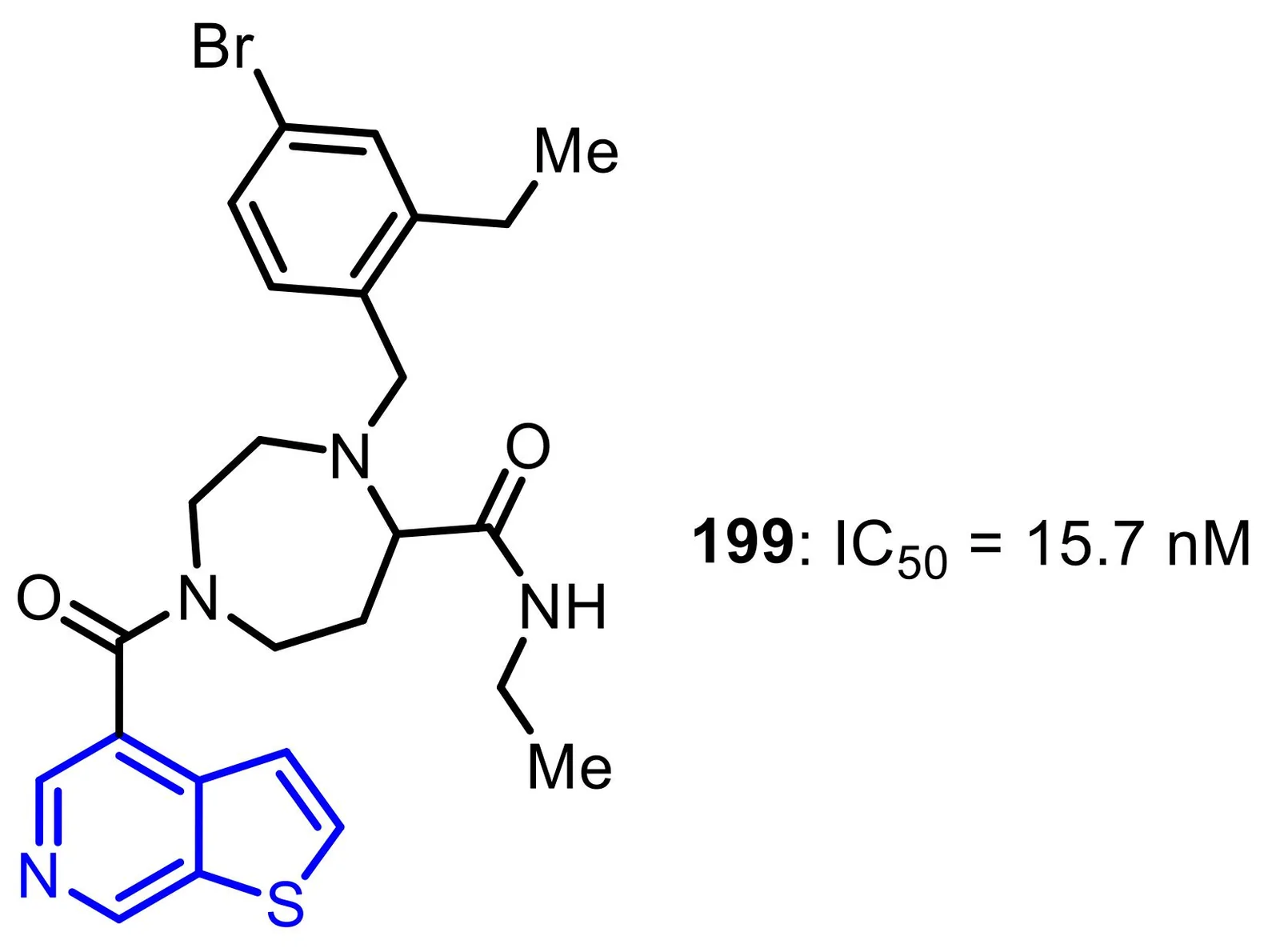
SARS-CoV--2 Mpro inhibitor based on thienopyridine scaffold.

In addition to antibacterial and antifungal applications, the thienopyridine scaffold has found utility in the development of antiviral agents, as demonstrated by Hazemann and colleagues in their hit-to-lead optimization of a SARS-CoV-2 Mpro inhibitor [140,141]. Starting from a computationally identified diazepane-based hit (IC_50_ = 14 µM), the authors combined high-throughput medicinal chemistry with structure-based design to systematically optimize binding interactions across the S1, S2, S1′, and S4 pockets of the Mpro active site. This strategy rapidly yielded compound **199** based on thienopyridine scaffold, a potent and selective Mpro inhibitor (IC_50_ = 15.7 nM) with a binding mode confirmed by X-ray crystallography, which revealed an extensive network of water-mediated interactions (Figure 6).

### 3.2. Utility of Thienopyridines in Cancer Therapy

In recent years, a large number of polyfunctionalized pyridines exhibiting significant cytotoxic activity against cancer cells or antiproliferative activity through inhibition of tumor-associated enzymes have been investigated [42,142,143,144,145]. The thienopyridine scaffold fits this trend as well [146].

**Figure 7:**
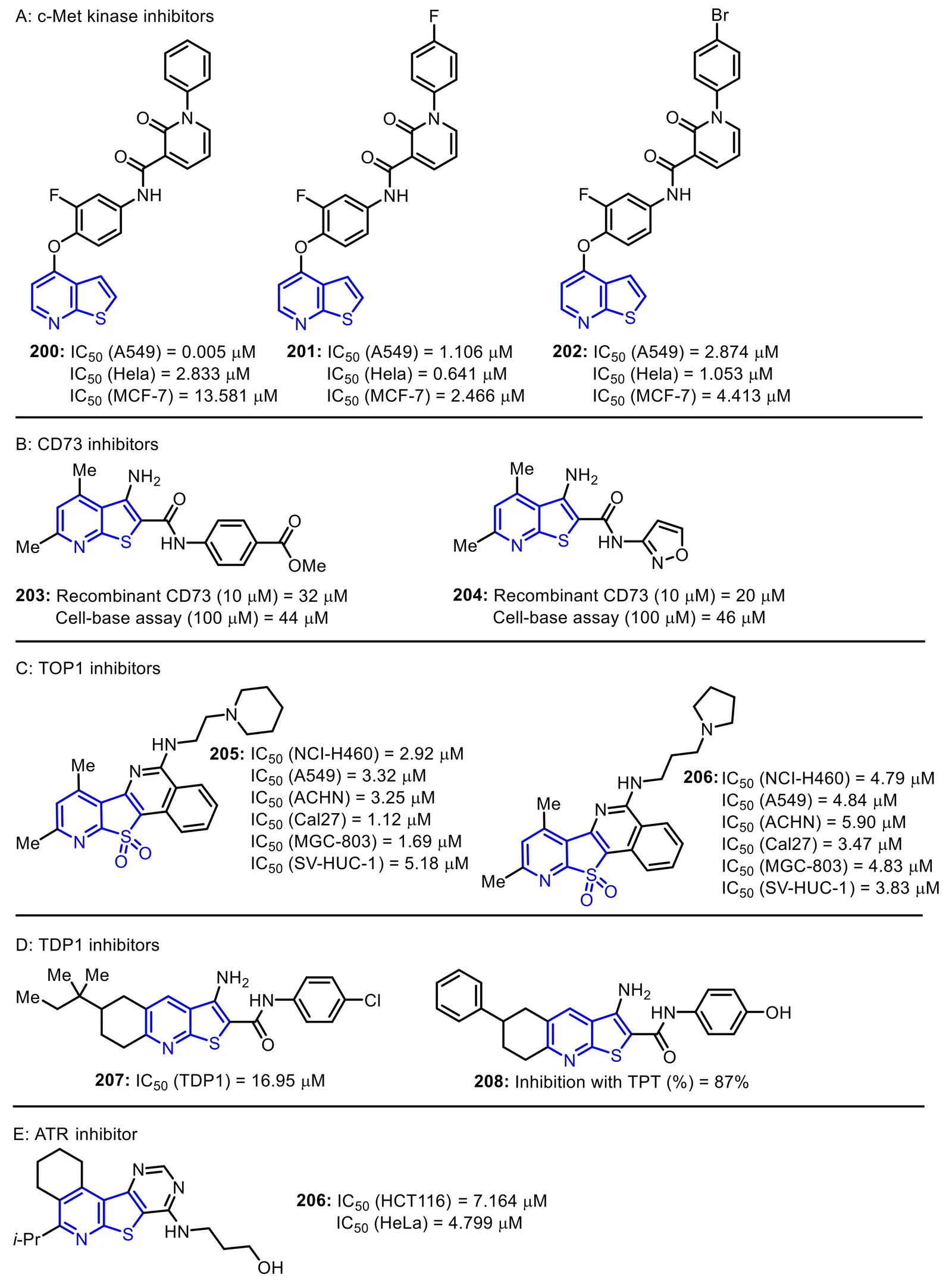
Thienopyridine-based inhibitors of tumor-associated enzymes: (**A**) c-Met kinase inhibitors; (**B**) CD73 inhibitors; (**C**) TOP 1 inhibitors; (**D**) TDP1 inhibitors; (**E**) ATR inhibitors.

The cellular–mesenchymal–epithelial transition factor (c-Met) tyrosine kinase promotes cell proliferation, migration, and survival, playing a key role in developmental morphogenesis, wound healing, and organ maintenance, whereas its dysregulated activation is associated with cancer progression, metastasis, and therapy resistance [147,148]. Wang and colleagues [67] reported c-Met kinase inhibitors based on thieno [2,3-*b*]pyridine substituted at position 4. The thienopyridine ring was synthesized according to the Gould–Jacobs method and then sequentially modified via oxidation, chlorination, alkylation, and amidation reactions to afford the target compounds. The cytotoxic study was carried out on three cell lines overexpressing c-Met kinases (A549, MCF-7, and HeLa cells). Compounds **200**, **201**, and **202** (Figure 7A) showed significant antiproliferative activity. Compound **200** demonstrated the best inhibitory activity with IC_50_ values of 0.005, 2.833, and 13.581 µM, respectively. Through computer modeling, the authors established that the key interaction of compound **200** in the kinase pocket is provided by the F atom of the aryl group; in its absence, the antiproliferative activity decreases. Furthermore, SAR analysis showed that replacement of the quinoline moiety with the more electron-rich thienopyridine scaffold was intended to strengthen target interactions, while retention of the pyridine nitrogen was critical for hydrogen bonding with Met1160. The introduction of a fluorine atom served to modulate hydrophobic contacts, whereas incorporation of a rigid pyridone-containing linker and electronic tuning of the terminal phenyl ring further optimized cellular and kinase activity.

5′-Ectonucleotidase (CD73) is a membrane glycoprotein that catalyzes the conversion of adenosine monophosphate (AMP) to adenosine and inorganic phosphate [149]. Free adenosine is known to possess immunosuppressive properties in the tumor microenvironment, promoting tumor growth and development [150]. In this regard, CD73 inhibition as a potential immunotherapeutic strategy has been widely investigated in recent years [151,152]. Grosjean and colleagues [153] obtained a new series of 4,6-disubstituted thienopyridines as CD73 inhibitors and evaluated their inhibitory activity in an enzymatic assay. The bicyclic compounds in this series displayed moderate inhibitory activity, with 4,6-dimethylthienopyridines **203** and **204** (Figure 7B)—bearing arylamide and oxazole-amide groups at the C2 position, respectively—exhibiting the highest potency. The therapeutic potential of these compounds was also confirmed by cytotoxic experiments on the MDA-MB-231 breast cancer cell line expressing CD73.

Topoisomerase I (TOP1) is a ubiquitous and essential enzyme involved in multiple DNA transactions, including replication, transcription, and chromosome dynamics, whose overexpression is associated with tumor proliferation, making it a popular target for anticancer therapy development [154,155]. Zheng and colleagues designed a series of isoquinoline-fused 1,1-dioxide-thieno [2,3-*b*]pyridines [156]. The most attractive results were obtained for compounds bearing alkylpiperidine **205** and alkylpyrrolidine **206** moieties (Figure 7C), which exhibited complete inhibition of TOP1 activity at 20 µM and 40 µM, respectively. The cytotoxic properties of the compounds were investigated on five human cancer cell lines (NCI-H460, A549, ACHN, Cal27, and TPT). Compounds demonstrating inhibitory activity against TOP1 also exhibited strong antiproliferative activity in cell tests and very low cytotoxicity against the normal cell line SV-HUC-1. Molecular docking performed for the most active compound (**205**) revealed that the studied molecule associates with TOP1 via four hydrogen bonds. The sulfonyl group forms two bonds with ARG364, whereas the other two are formed between the side-chain atoms of the molecule and ASP533. Deeper biological studies of molecule **205** demonstrated that the compound also suppresses c-MYC inducing autophagy and apoptosis of cancer cells. In vivo studies on human Cal27 oral cancer xenografts confirmed that **205** exhibits promising anticancer efficacy, achieving 64.7% tumor growth inhibition (TGI) at 20 mg/kg without significant toxicity.

Despite the cytotoxic efficacy of TOP1 inhibitors, cases of tumor cell resistance to drugs working through this mechanism are known. One possible way to overcome resistance may be the inhibition of TDP1–tyrosyl-DNA phosphodiesterase 1, an enzyme involved in DNA repair [157,158]. In search of new antitumor agents that enhance the activity of existing chemotherapy, Dobbs with colleagues synthesized a series of amides of 3-amino-5,6,7,8-tetrahydrothieno [2,3-*b*]quinoline-2-carboxylic acids [159]. Five compounds exhibited good suppression of enzyme activity with IC_50_ values below 50 µM, with compound **207** (Figure 7D) being the most promising (IC_50_ = 16.95 µM). Although these carboxamides showed no antiproliferative activity against colorectal cancer (HCT-116) and triple-negative breast cancer (MDA-MB-231) cell lines, evaluation of their synergistic action with Topotecan revealed that most compounds were capable of potentiating its effect. Compound **208** (Figure 7D) exhibited the greatest enhancement, achieving 87% inhibition compared to 70% for Topotecan alone. Thus, the possibility of using thieno [2,3-*b*]pyridines as adjuvants for the antitumor therapy is confirmed.

Ataxia-telangiectasia and Rad3-related protein (ATR) is a kinase belonging to the PIKK family and is involved in DNA repair pathways. Elevated ATR levels have been noted as a biomarker in various types of cancer, making it a therapeutic target for anticancer therapy [160]. An international group of medicinal chemists designed ATR kinase inhibitors based on thieno [2,3-*b*]pyridines fused with a pyrimidine ring [161]. The anticancer potency of compounds selected after the kinase inhibition assay and molecular modeling was evaluated against HCT116 and HeLa cancer cell lines, for which the ATR plays a key role. All investigated compounds demonstrated cytotoxic activity in the micromolar range for both cell lines. Among series, compound **209** (Figure 7E) was the most potent against both cell lines, with IC_50_ of 7.164 µM and 4.799 µM for the HCT116 and HeLa cell lines, respectively. The results obtained in biological tests correlate with the results of molecular docking, in which **206** had the best binding to ATR.

**Figure 8:**
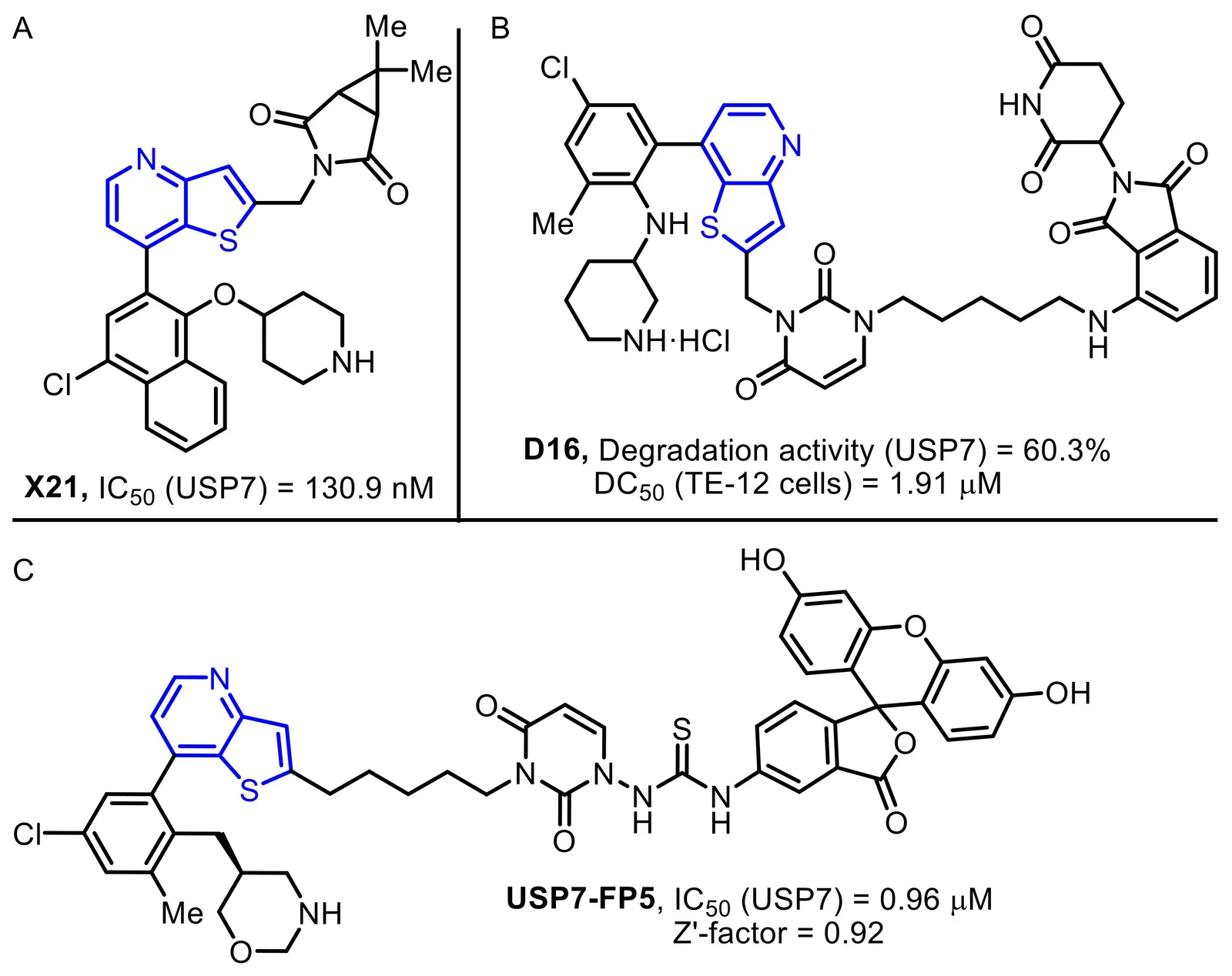
USP7-targeted thienopyridines: (**A**) structure and IC_50_ value of compound **X21**; (**B**) structure and DC_50_ value of compound **D16**; (**C**) structure and IC_50_ value of compound **USP7-FP5**.

Ubiquitin-specific protease 7 (USP7) is a deubiquitinating enzyme that plays a crucial role in regulating protein homeostasis by removing ubiquitin tags from target proteins, thereby preventing their proteasomal degradation [162]. Overexpression of USP7 has been frequently observed in various cancers, including upper gastrointestinal (UGI) cancer, where it promotes cancer cell survival, growth, and metastasis through stabilization of oncoproteins such as p53, FOXM1, and others [163,164,165]. The known USP7 inhibitor CP41 [166] has been utilized as a versatile starting point for several independent medicinal chemistry campaigns. First, Zhang and colleagues [167] employed a scaffold-hopping strategy to generate a series of novel USP7 inhibitors derived from CP41, identifying compound **X21** as potent and selective USP7 inhibitors (Figure 8A). It not only modulated established USP7 substrates (MDM2, p53, and TRIM27) but also reduced the level of PCLAF, a key factor in translesion synthesis, and demonstrated in vivo antitumor efficacy in colon cancer models. Second, Ma and colleagues [168] went a different way, modified CP41 to a series of proteolysis-targeting chimeras (PROTACs [169]) by conjugating it with CRBN ligands via linkers of varying length and structure. This effort led to compound **D16**, a potent and selective USP7 degrader with DC_50_ values of 1.91 µM in TE-12 cells, which functions through the ubiquitin–proteasome system by forming a ternary complex with USP7 and CRBN. Chen and colleagues [170] focused on assay development, utilizing CP41 as a scaffold to construct two fluorescence polarization (FP) probes by conjugating its solvent-exposed succinimide moiety with fluorescein 5-isothiocyanate (FITC) via various linkers. **USP7-FP5** was identified as the optimal probe due to its superior signal window and excellent Z′-factor (0.92), providing a cost-effective, robust, and HTS-compatible method for USP7-targeted drug discovery.

**Figure 9:**
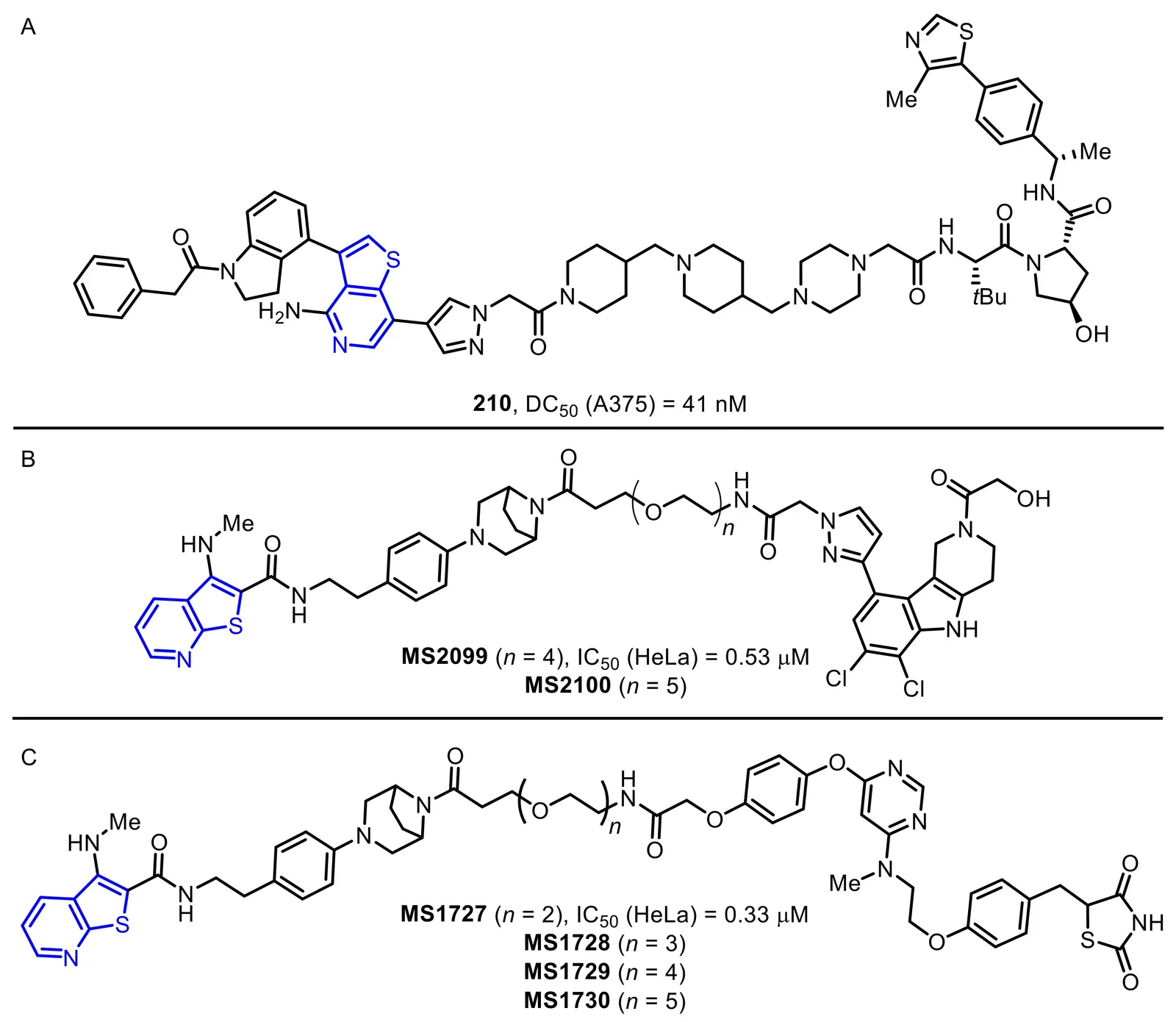
Utilization of thienopyridines in PROTACS and DUBTACs developments: (**A**) structure and DC_50_ value of compound **210**; (**B**) structures of compounds **MS2099** and **MS2100**; (**C**) structures of compounds **MS1727**, **MS1728**, **MS1729**, **MS1730**.

Zhang and colleagues reported another example of PROTAC development based on a thienopyridine scaffold, specifically targeting receptor-interacting protein kinase 1 (RIPK1) [171]. Utilizing their Rapid-TAC platform, the authors rapidly identified the optimal E3 ligase (VHL) and linker length, leading to the development of compound **210**, a potent and selective RIPK1 degrader featuring a rigid piperidine-containing linker (Figure 9A). Compound **210** achieved remarkable degradation efficacy with a DC_50_ of 41 nM and a Dmax of 97% in human A375 melanoma cells, and exhibited excellent selectivity in global proteomic analysis with no other proteins significantly affected. In vivo, **210** demonstrated favorable pharmacokinetics and effectively degraded RIPK1 in both B16F10 syngeneic and A375 humanized mouse models. Crucially, combination of **210** with X-ray radiotherapy (XRT) significantly suppressed tumor growth and extended median survival in both models (from 19 to 24 days in B16F10; from 36 to 51 days in A375 humanized mice), whereas the literature RIPK1 inhibitor GSK074 showed no therapeutic benefit. This study represents the first demonstration of in vivo antitumor efficacy of a RIPK1-targeting PROTAC in combination with radiotherapy in a human cancer model and the advantage of targeting RIPK1′s nonenzymatic scaffolding function through degradation over kinase inhibition alone.

In addition to the PROTAC technique, in recent years deubiquitinase-targeting chimeras (DUBTACs) have been in the significant spotlight of medicinal chemistry [172]. In contrast with PROTACs, which eliminate disease-causing proteins by recruiting E3 ubiquitin ligases to tag them for proteasomal destruction, DUBTACs operate via the opposite mechanism: they recruit deubiquitinases (DUBs) to remove ubiquitin chains from polyubiquitinated proteins, thereby preventing their degradation and enhancing their steady-state levels. This approach is particularly attractive for targeting tumor suppressors and other proteins whose loss of function contributes to disease pathogenesis. Wang and colleagues [173] made an important contribution in this field by exploiting the thienopyridine-bearing USP28 ligand CT1073 [174] to develop a novel class of DUBTACs. By conjugation CT1073/KL9 to ligands for CFTR, cGAS, or PPARγ via various linkers, the authors generated the first-in-class USP28-recruiting DUBTACs that effectively stabilized these respective target proteins. Moreover, cGAS-targeting DUBTACs (**MS2099** and **MS2100**) activated the cGAS-STING pathway and exhibited antiproliferative effects in HeLa cells (Figure 9B). Similarly, the PPARγ-targeting DUBTACs (MS1727–MS1730) potently stabilized PPARγ and suppressed HeLa cell proliferation with IC_50_ values as low as 0.33 µM (Figure 9C).

**Figure 10:**
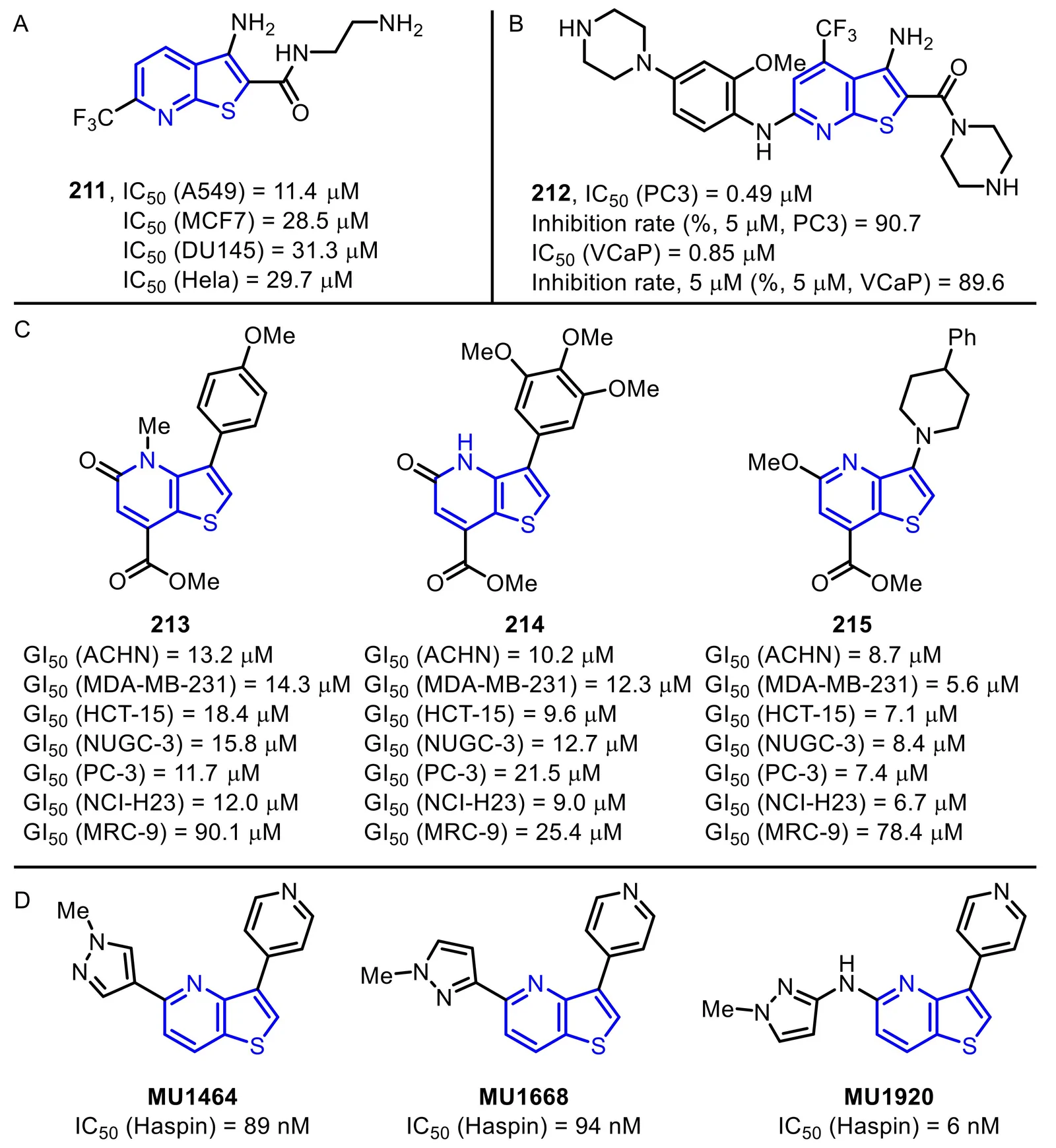
Anti-cancer activity of thienopyridine derivatives: (**A**) structure and IC_50_ values of compound **211**; (**B**) structure and IC_50_ values of compound **212**; (**C**) structures and GI_50_ values of compounds **213**–**215**; (**D**) structures and IC_50_ values of compounds **MU1463**, **MU1688**, and **MU1920**.

Trifluoromethyl-substituted 3-amino-thieno [2,3-*b*]pyridine-2-carboxamides and pyrido [3′,2′:4,5]thieno [3,2-*d*]pyrimidin-4(3*H*)-ones were synthesized and evaluated for anticancer activity against four human cancer cell lines (A549, MCF7, DU145, and HeLa) using MTT assays [175]. It was found that bicyclic structures show better activity in cell tests than tricyclic ones, which the authors attribute to the presence of free amino and amide groups. Compound **211** (Figure 10A) showed the most pronounced activity, amounting to 11.4 µg/mL on the lung cancer cell line A549 (CCL-185).

Tu and colleagues [176] designed and synthesized a series of twenty-four novel thieno [2,3-*b*]pyridine derivatives and evaluated their in vitro antiproliferative activity against PC3 and VCaP prostate cancer cell lines, without prior assumption of a specific molecular target. Among the synthesized compounds, agent **212** (Figure 10B) emerged as the most potent candidate (IC_50_ = 0.49 µM and 0.85 µM, respectively). Mechanistic studies revealed that **209** concentration-dependently induced apoptosis and G0/G1 cell cycle arrest and significantly suppressed PCa cell migration and invasion. Subsequent target identification and validation studies demonstrated that **212** efficiently inhibited BLM helicase-mediated DNA unwinding (IC_50_ = 3.17 µM), outperforming the lead compound TC1, and exhibited higher binding affinity to BLM protein (Kd = 0.74 µM) than to DNA, competitively interfering with BLM-DNA substrate interaction. Molecular docking revealed stable binding of **212** to key residues in the BLM RQC domain through multiple hydrogen bonds. Overall, compound **212** represents a promising lead compound for the development of novel BLM-targeted anti-prostate cancer agents.

Sung and colleagues [177] synthesized a series of 3-aryl-substituted thieno [3,2-*b*]pyridin-5(4*H*)-ones via a regioselective BOP-promoted aza-[3 + 3] cycloaddition between 3-aminothiophenes and *α*,*β*-unsaturated carboxylic acids, followed by base-induced dehydrogenation. The compounds were evaluated for antiproliferative activity against six human cancer cell lines (ACHN, MDA-MB-231, HCT-15, NUGC-3, PC-3, and NCI-H23). SAR analysis revealed that electron-rich *para*-aryl substituents at the C3 position and compact basic heterocycles significantly enhance cytotoxicity, with the most potent compounds, **214** (3,4,5-trimethoxyphenyl) and **215** (4-piperidinophenyl) (Figure 10C), exhibiting GI_50_ values below 10 µM. Notably, several active compounds, including **213** and **215**, demonstrated favorable selectivity with low cytotoxicity against normal MRC-9 lung fibroblasts.

Finally, Moyano and colleagues [178] identified the thieno [3,2-*b*]pyridine scaffold as a versatile platform for the development of highly selective kinase inhibitors targeting underexplored protein kinases. Through systematic exploration of substitution patterns around this core, the authors discovered that the weakly interacting thieno [3,2-*b*]pyridine moiety allows for adoption of profoundly different binding modes while maintaining excellent kinome-wide selectivity, as demonstrated by the isomeric compounds **MU1464** and **MU1668** (Figure 10D). Optimization of the series led to identification of **MU1920**, a highly potent and selective inhibitor of the mitotic kinase Haspin (IC_50_ = 6 nM), which fulfills the criteria for a quality chemical biology probe. X-ray crystallography revealed that **MU1920** binds in the ATP pocket with a binding mode analogous to that of **MU1668**, anchored via interactions with the kinase back pocket rather than the hinge region. Cellular studies confirmed efficient Haspin inhibition (IC_50_ = 94 nM in HT29 cells) and high selectivity in kinobeads assays across 262 quantified kinases. Despite its excellent selectivity and favorable pharmacokinetic properties (oral bioavailability 53% in mice), **MU1920** did not exhibit significant cytotoxicity in cancer cells, suggesting that Haspin inhibition alone is insufficient for antiproliferative effects and that the activity of previously reported Haspin inhibitors may be partly due to off-target effects. Importantly, the thieno [3,2-*b*]pyridine scaffold also showed potential for targeting other underexplored kinases, including CDKL family members.

### 3.3. Utility of Thienopyridines in Medication of Neurological and Neuropsychiatric Diseases

**Figure 11:**
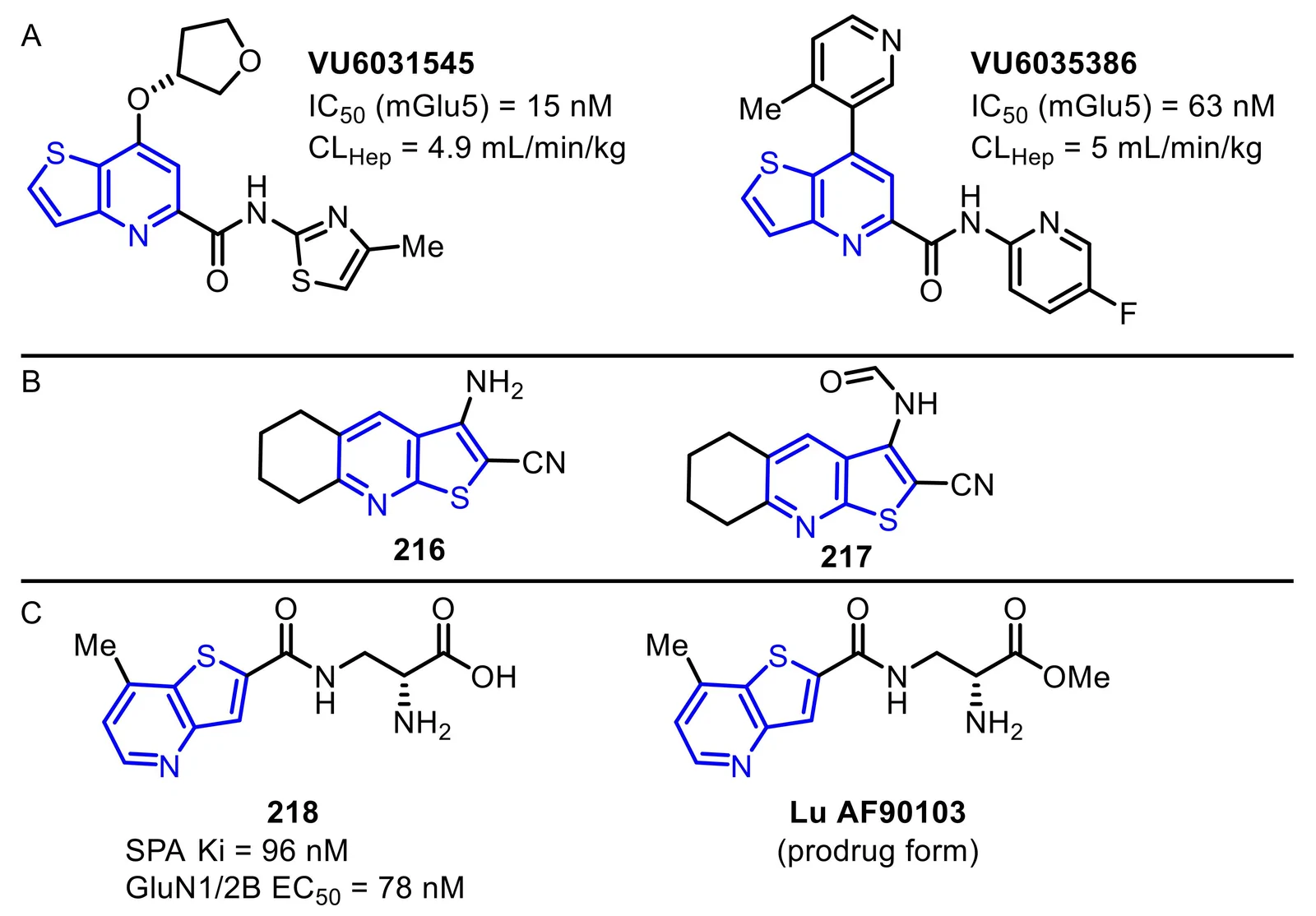
Structures of thienopyridines designed for the medication of neurological and neuropsychiatric diseases: (**A**) structures and IC_50_ values of compounds **VU6031545** and **VU6035386**; (**B**) structures of compound **216** and **217**; (**C**) structures of compound **218** and its prodrug form.

The utility of the thienopyridine scaffold in the design of agents acting on central nervous system (CNS) targets was demonstrated in several works of the Lindsley group [179,180,181]. In the first [179], 3-aminothieno [2,3-*b*]pyridine-2-carboxamides were systematically screened as M4 muscarinic receptor positive allosteric modulators (PAMs); however recognized lead compound (**VU6008677**) was a furo [2,3-*b*]pyridine derivative. In the second work [180], thieno [3,2-*b*]pyridine-5-carboxamides were investigated as negative allosteric modulators (NAMs) of the metabotropic glutamate receptor mGlu5. Compound **VU6031545** (Figure 11A) exhibited nanomolar activity against human mGlu5 (IC_50_ = 15 nM), good in vitro/in vivo clearance agreement, and high CNS penetration. It was mentioned that replacing the thienopyridine with another heterocycle also made it possible to obtain active compounds; however, their hepatic clearance in rats and/or binding to plasma proteins in humans were high. In the third study [181], a scaffold-hopping strategy was employed to replace the ether-linked tetrahydrofuran moiety of **VU6031545** with carbon-linked biaryl/heteroaryl groups, leading to the discovery of 7-(pyridin-3-yl)thieno [3,2-*b*]pyridine-5-carboxamide **VU6035386** (Figure 11A). This compound displayed low nanomolar potency against human mGlu5 (IC_50_ = 63 nM), high brain penetration (rat Kp = 10.6, Kp,uu = 1.11), and a significantly improved predicted human hepatic clearance (5 mL/min/kg) compared to the predecessor **VU6031545**. Alternative cores, such as 1-methyl-1*H*-pyrrolo [2,3-*b*]pyridine or quinoline, were also investigated. Nevertheless, as in the previous study, such compounds exhibited higher clearance and plasma protein-binding values compared to thieno [3,2-*b*]pyridine analogues.

In addition to the Lindsley’s researchers, Asao and colleagues [182] reported the ability of thienopyridines to induce neuronal differentiation of PC12 cells. During screening of a library of 12,000 aromatic compounds for neuritogenic activity, two hits with a thienopyridine scaffold were identified (**216**, **217**), and one of them—3-amino-5,6,7,8-tetrahydrothieno [2,3-*b*]quinoline-2-carbonitrile (compound **216**; Figure 11B)—was selected for detailed study. SAR analysis revealed that both the fused cyclohexyl ring and the free cyano group at C2 are essential for activity. Compound **216** induced Tuj-1 expression in PC12 cells comparable to NGF but retained activity in NGF-insensitive PC12 IS cells, indicating a TrkA-independent mechanism involving direct activation of MAPK/ERK and PI3K/Akt pathways, consistent with its ability to penetrate the cell membrane. Additionally, **216** exhibited strong intrinsic fluorescence with a large Stokes shift and solvatochromic properties, leading the authors to propose it as a bifunctional tool—both a small-molecule NGF substitute for neuronal differentiation and a fluorescent probe for bioimaging.

Building upon the antidepressant potential of D-cycloserine, a partial agonist at the glycine site of NMDA receptors [183], Ascic and colleagues [184] employed a structure-based drug design strategy to discover a novel series of aminopropanoic acid derivatives as GluN2B-selective partial agonists. Among these, the pyridothiophene-containing compound **218** (Figure 11C) was recognized as a potent partial agonist at GluN1/GluN2B (EC_50_ = 78 nM, 24% efficacy) with a favorable in vitro profile. Due to poor blood–brain barrier penetration of **218**, the authors developed the methyl ester prodrug (**Lu AF90103**), which effectively delivered **218** into the rat brain as confirmed by microdialysis studies. In rodent models, **Lu AF90103** demonstrated acute pharmacodynamic effects in seizure and EEG tests, inducing high-frequency oscillations similar to ketamine but without affecting locomotor activity, suggesting a reduced psychotomimetic liability. A single dose of **Lu AF90103** produced a sustained antidepressant-like effect for 24 h after administration in the Porsolt swim test and increased basal synaptic transmission in the stress-sensitive hippocampal pathway.

### 3.4. Utility of Thienopyridines in Medication of Metabolic Disorders

**Figure 12:**
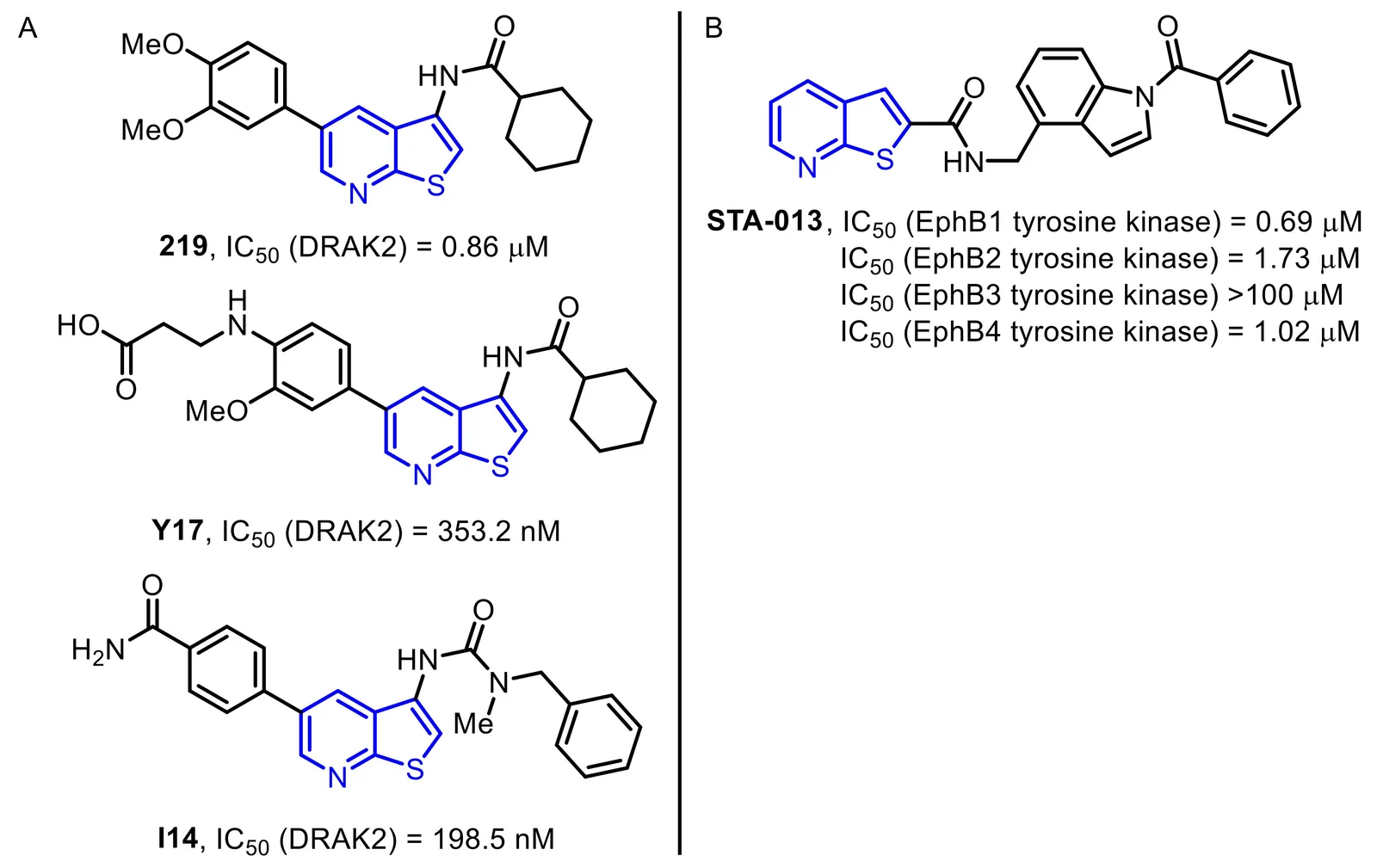
Structures of thienopyridines designed for metabolic disorder medication: (**A**) structures and IC_50_ values of compounds **219, Y17,** and **I14**; (**B**) structure and IC_50_ values of compound **STA-013**.

One more therapeutic area where the thienopyridine scaffold has been discussed as a desired structural motif is the treatment of metabolic disorders and associated diseases. In this context, thienopyridine derivatives have been primarily investigated as inhibitors of death-associated protein-related apoptosis-inducing protein kinase (commonly known as DRAK, or STK17), which plays a critical role in pancreatic β-cell function and insulin signaling [185]. A previously reported thieno [2,3-*b*]pyridine-based dual DRAK1/DRAK2 inhibitor (**219**; IC_50_ = 0.86 μM; Figure 12A) [186] showed poor oral bioavailability and lacked tissue selectivity. To address these limitations, Lian and colleagues [187] performed systematic modification of the 3,4-dimethoxyphenyl moiety of the thieno [2,3-*b*]pyridine scaffold, demonstrating that peripheral aryl substitution strongly influenced both DRAK2 inhibitory potency and pharmacokinetic behavior. This optimization led to compound **Y17** (Figure 12A), a carboxyl-containing derivative with improved DRAK2 potency (IC_50_ = 353 nM), moderately enhanced oral absorption, and markedly increased pancreatic tissue selectivity relative to **219**. In vitro and in vivo, **Y17** restored mitochondrial function, protected *β*-cells from apoptosis, reduced macrophage IL-1β secretion, and improved glucose tolerance at a lower dose than the reference compound (**219**). Shu and colleagues also used the previously reported DRAK2 inhibitor **219** [186] as a starting point but pursued a distinct optimization strategy, replacing the amide moiety with a ureido group to extend molecular length and improve binding complementarity within the kinase pocket [188]. This modification led to the lead candidate **I14** (Figure 12A), which demonstrated potent DRAK2 inhibition (IC_50_ = 198.5 nM) and dose-dependently enhanced glucose-stimulated insulin secretion (GSIS) in primary mouse pancreatic islets. In vitro, **I14** restored mitochondrial membrane potential (MMP), protected INS-1E cells from palmitic acid-induced apoptosis, and promoted autophagic flux through modulation of the DRAK2–ULK1 signaling axis. Notably, oral administration of I14 significantly improved glucose tolerance in an OGTT in mice, marking it as the first urea-containing thieno [2,3-*b*]pyridine-based DRAK2 inhibitor with in vivo glucose-lowering efficacy.

Moreover, Tareq and colleagues [189] developed a series of thienopyridine-based pan-EphB tyrosine kinase inhibitors (STA analogs) for the treatment of metabolic syndrome, leveraging the resolved X-ray crystal structure of the EphB1 kinase domain. Through structure-based drug design and optimization, compound **STA-013** (Figure 12B) was identified as a potent and selective inhibitor of EphB1, EphB2, and EphB4 (IC_50_ = 0.69, 1.73, and 1.02 µM, respectively), with no activity against EphB3 or the majority of 140 kinases profiled. In high-fat diet-induced obese mice, intraperitoneal administration of **STA-013** (25 mg/kg/day) significantly reduced body weight and fat mass while preserving lean muscle mass, improved glucose clearance and insulin sensitivity, and decreased liver fat deposition. Mechanistically, **STA-013** inhibited EphB phosphorylation, increased p-AKT/AKT signaling, and elevated InsR-β protein levels in both liver and brown adipose tissue, reversing insulin-induced insulin receptor degradation and restoring insulin signaling. Transcriptomic analysis revealed upregulation of genes involved in glucose metabolism and insulin regulation (Ins2, Lepr, Stat3) and downregulation of lipogenic genes (Fasn, CD36, Acat1). This study demonstrates that pharmacological inhibition of EphB1/2/4 tyrosine kinases represents a promising therapeutic strategy for metabolic syndrome, offering an alternative to GLP-1 agonists with the advantage of preserving lean muscle mass.

Taken together, the available SAR data indicate that the thienopyridine moiety should be regarded less as an activity-determining pharmacophore in isolation and more as a chemically adaptable heteroaromatic platform whose biological profile is strongly modulated by peripheral substitution and scaffold elaboration. This interpretation is consistent with the broad structural diversity and therapeutic applications reported for thienopyridines and their derivatives, as well as with studies showing that thienopyridine core substitution can markedly alter potency, selectivity, pharmacokinetic properties, and even the ligand-binding mode [32,178].

### 3.5. Agrochemical Applications of Thienopyridines

Beyond medicinal chemistry, thienopyridines, along with other polyfunctionalized pyridines [190,191,192,193], have found use in the development of insecticidal agrochemicals, which considerably broadens the biological activity profile of this heterocyclic scaffold.

**Figure 13:**
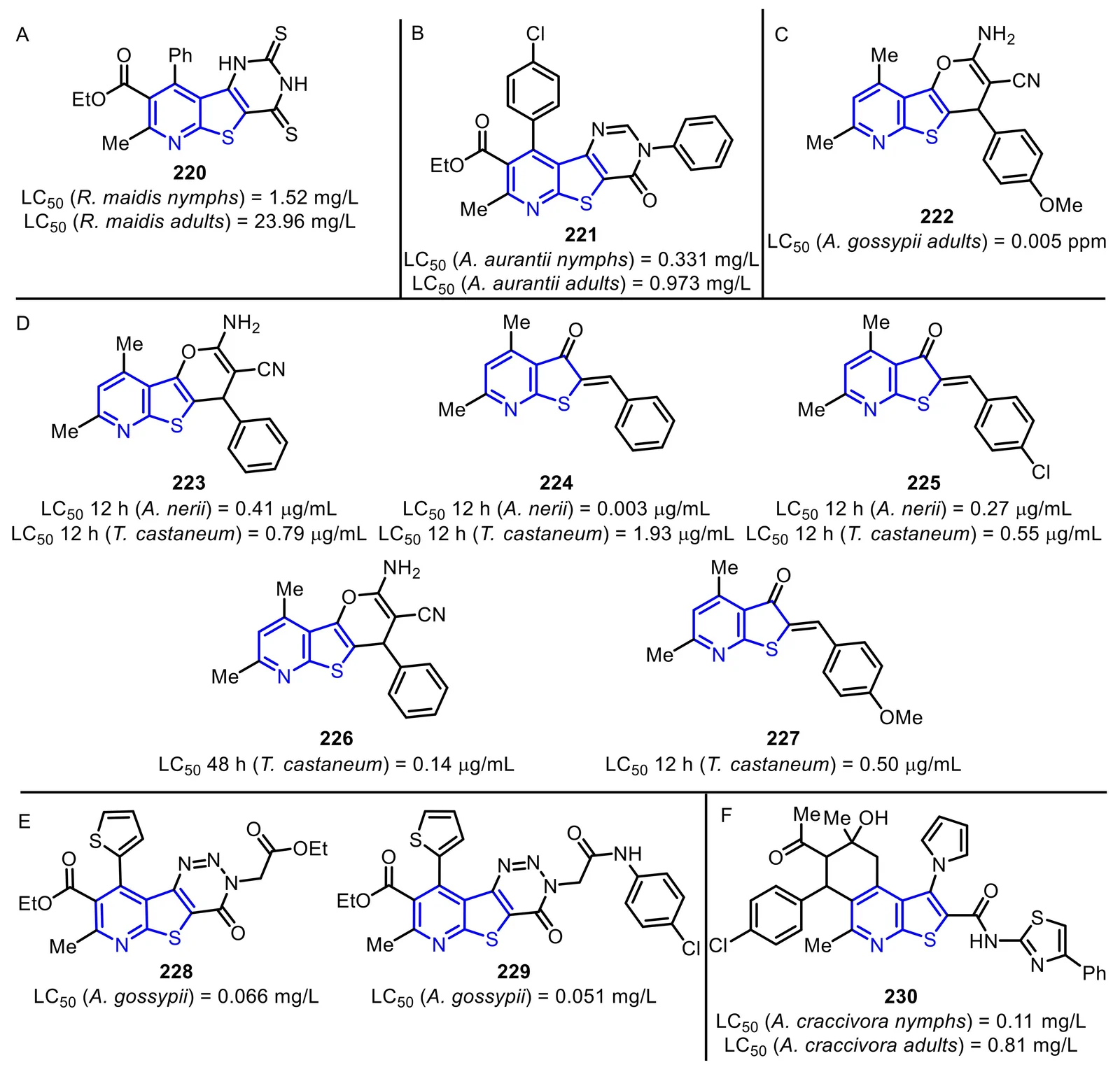
Insecticidal activity of thienopyridine derivatives: (**A**) structure and LC_50_ values of compound **220**; (**B**) structure and LC_50_ values of compound **221**; (**C**) structure and LI_50_ value of compound **222**; (**D**) structures and LC_50_ values of compounds **223**–**227**; (**E**) structures and LC_50_ values of compounds **228** and **229**; (**C**) structure and LI_50_ value of compound **222**; (**F**) structure and LC_50_ values of compound **230.**

Thabet and coauthors synthesized a series of polyfunctionalized thienopyridines and tested them as insecticides against nymphs and adults of the corn aphid (*R. maidis*) [194]. Investigated pyridine derivatives showed significant insecticide activity with LC_50_ values ranging from 1.52 to 18.45 mg/L for nymphs and from 23.96 to 80.73 mg/L for imagoes, with thieno [2,3-*b*]pyridine-containing compound **220** (Figure 13A) showing the highest activity (LC_50_ values = 1.52 and 23.96 mg/L, respectively). Its molecular docking with acetylcholinesterase revealed high binding affinity to the target enzyme.

Bakhite and colleagues obtained a series of potential insecticides targeting the California red scale (*A. aurantii*), a major pest of citrus crops [195]. Candidates were evaluated on nymphs and adults of *A. aurantii*. One of the highest activities against both developmental stages was exhibited by thienopyridine compound **221** (LC_50_ 0.331 and 0.973 mg/L, respectively; Figure 13B). Even higher activity was shown by 2-methyl-thioxo-1,6-dihydropyridine-3-carboxylate with LC_50_ (nymphs *A. aurantii*) = 0.202 mg/L and LC_50_ (adults *A. aurantii*) = 0.818 mg/L. These values were comparable to those of the reference insecticide acetamiprid (LC_50_ = 0.187 and 0.746 mg/L). Notably, the two most active compounds bear a 4-chlorophenyl fragment, a structural feature also present in the widely used insecticide dichlorodiphenyltrichloroethane (DDT).

Abdul-Malik and colleagues performed two studies in this area [196,197]. In the first [196], they synthesized a series of polyfunctionalized thieno [2,3-*b*]pyridine derivatives and evaluated their insecticidal activity against the cotton aphid *Aphis gossypii* using the leaf-dip technique. Among the tested compounds, pyrano-thienopyridines exhibited the highest potency, with compound **222** (Figure 13C) showing the most remarkable efficacy (LC_50_ = 0.005 ppm), significantly outperforming the reference insecticide acetamiprid (LC_50_ = 0.045 ppm). DFT calculations revealed that the most active compounds possessed the highest positive and negative electrostatic potentials, while molecular docking against acetylcholinesterase (AChE) demonstrated strong binding affinities (Vina scores up to −9.8 kcal/mol), correlating well with the observed insecticidal activity. In the second study [197], the insecticidal activity of a series of thienopyridine derivatives was evaluated against *A. nerii* and *T. castaneum* using leaf-dip and residue-film bioassays over 12, 24, and 48 h. Against *A. nerii*, compounds **223**, **224**, and **225** (Figure 13D) exhibited exceptionally high potency with LC_50_ values of 0.41, 0.003, and 0.27 µg/mL at 12 h, respectively, while against *T. castaneum*, compounds **223**, **224**, and **227** showed LC_50_ values of 0.79, 0.55, and 0.50 µg/cm^2^ at 12 h. At 48 h, compounds **224**, **225**, **227** reached 100% mortality against *T. castaneum*, precluding LC_50_ calculation, while compound **226** demonstrated the lowest LC_50_ (0.14 µg/cm^2^). SAR analysis revealed that the presence of amino, cyano, phenyl, and methoxyphenyl substituents significantly enhances insecticidal potency.

Khamies and colleagues screened the insecticidal activity of ethyl nicotinate derivatives against nymphs and adults of the cotton aphid (*A. gossypii*) [28]. The most pronounced effect was shown by compounds **228** and **229** (Figure 13E), whose LC_50_ values (0.66 and 0.051 mg/L for nymphs, respectively) were close to those of acetamiprid (0.045 mg/L).

The same research group synthesized 1-amino-6,7,8,9-tetrahydrothieno- [2,3-*c*]isoquinoline-2-carboxamides and their 1-(1-pyrrolyl)-substituted derivatives [198]. The insecticidal activity of all synthesized compounds was evaluated against nymphs and adults of the aphid *A. craccivora* in comparison with acetamiprid. In general, the 1-(1-pyrrolyl)-substituted derivatives exhibited higher activity than the parent amines. The most potent compound was **230** (Figure 13F), with LC_50_ values of 0.11 and 0.81 mg/L against nymphs and adults, respectively, which are comparable to those of acetamiprid.

**Figure 14:**
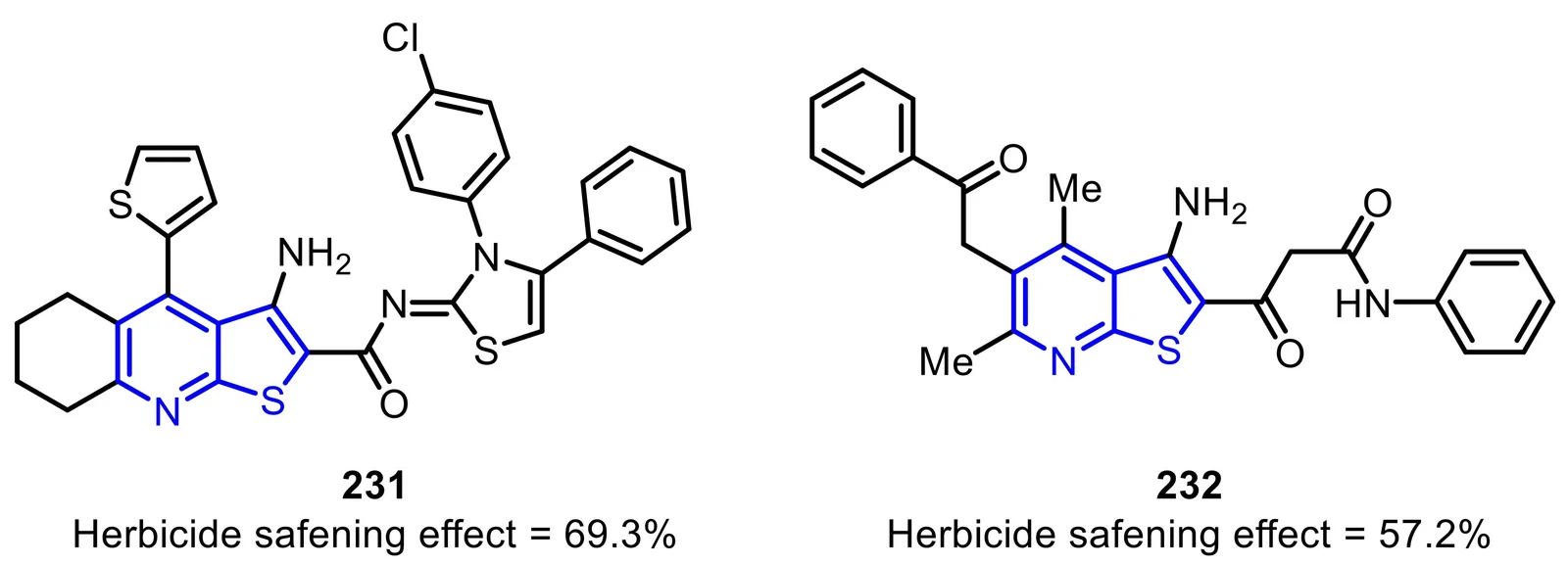
Thienopyridine moiety with herbicide antidote properties.

In addition to direct insecticidal action, thienopyridine derivatives can exhibit herbicide antidote properties, which is of independent interest for the protection of agricultural crops. Two recent studies have explored structurally distinct thienopyridine-based scaffolds (thieno [2,3-*b*]quinoline/2-iminothiazoline hybrids [199] and thieno [2,3-*b*]pyridine-substituted *β*-ketoamides [200]) as safeners against the widely used herbicide 2,4-dichlorophenoxyacetic acid (2,4-D). Compounds from both series were evaluated for antidote activity against 2,4-D in laboratory germination tests and subsequently in field trials on sunflower crops with yields from 50.4% to 70.9%. In the field, the 2-iminothiazoline/thienoquinoline hybrid **231** (tested on sunflower cv. Flagman; Figure 14) provided yield increases of 69.3% relative to the herbicide-only control, surpassing the reference antidote sulfalene (29.9%) [199]. Meanwhile, the thienopyridine β-ketoamide **232** (tested on sunflower cv. Master) achieved yield gains of 57.2% under similar field conditions [200]. Despite these promising results, complete neutralization of 2,4-D phytotoxicity was not achieved in either series, indicating that further structural optimization is required.

## 4. Conclusions

The developments reported from 2024 to the present underscore a significant transition in thienopyridine chemistry. Synthetically, while the Thorpe–Ziegler cyclization remains a foundational method for thieno [2,3-*b*]pyridine construction, its evolution is characterized by incremental improvements in substrate scope and one-pot efficiency rather than fundamental mechanistic shifts. In contrast, the field is experiencing a transformative surge in pyridine ring annulation, driven by the integration of photocatalysis, electrochemistry, and advanced metal catalysis. The rising prominence of multicomponent reactions and the strategic functionalization of preformed scaffolds into complex tri- and poly-heterocyclic systems (such as pyrimidine- and indole-fused derivatives) have fundamentally expanded the accessible chemical space of this privileged scaffold.

At the same time, many of these methods—particularly those based on pyridine ring annulation—have not yet been validated for the preparation of a broad structural diversity of thienopyridines. In most cases, thienopyridines appear only as minor representative examples within a wider reaction scope. Furthermore, although modern green chemistry approaches such as electro- and photocatalytic processes are being actively applied to their synthesis, we found no examples of mechanochemistry being used in the synthesis of thienopyridines. Given the success of this concept specifically in the context of green chemistry, such studies are likely to appear in the very near future.

This expanded structural diversity has directly fueled a broader biological profile. The thienopyridine scaffold has evolved from a traditional antimicrobial and anticancer pharmacophore into a complex tool for cutting-edge therapeutic approaches, most notably in the development of PROTACs and DUBTACs. Furthermore, its emerging roles in metabolic regulation (via DRAK2 and EphB inhibition) and CNS modulation further illustrate this transition. When coupled with the high potency observed in recent agrochemical discoveries, these trends confirm that thienopyridines are not merely established heterocycles, but a versatile platform poised to drive advances in both medicinal chemistry and crop protection.

Despite these advances, several challenges remain. Many studies are fragmentary and lack robust, large-scale SAR data, particularly in the search for antimicrobial agents. Systematic comparisons of all possible thienopyridine isomers are also scarce. Moreover, experiments are often performed under different conditions, which hampers comparison of the results obtained by different research groups in different studies. Finally, molecular modeling is still used predominantly to rationalize known trends rather than to predict promising candidates. An additional challenge concerns pharmacokinetic considerations: although the planar polycyclic structure of the thienopyridine fragment is favorable for stacking interactions and, consequently, for improved binding to target biomacromolecules, this very planarity is one of the main reasons for the poor solubility of these compounds. Accordingly, the search for new solutions to improve the pharmacokinetics of thienopyridines represents an important direction for the design of medicinal agents based on this scaffold. Nevertheless, the great interest of the scientific community in thienopyridines—which we hope this review has helped to demonstrate—is evident, and there is therefore every reason to expect that these challenges will be successfully addressed in the near future.

## Data Availability

No new data were created or analyzed in this study. Data sharing is not applicable to this article.
